# Water-soluble imidazolium-functionalized Cu(ii) complex as a recyclable catalyst for allylic and benzylic oxidation

**DOI:** 10.1039/d5ra09017b

**Published:** 2026-01-05

**Authors:** X. Huo, M. Guagliardo, X. Duke, B. Bouley, A. E. V. Gorden

**Affiliations:** a Department of Chemistry and Biochemistry, Texas Tech University Lubbock Texas 79430 USA anne.gorden@ttu.edu

## Abstract

A water-soluble imidazolium-functionalized Cu(ii) catalyst, used in combination with *tert*-butyl hydroperoxide (TBHP), enables benzylic and allylic oxidations in H_2_O. The reaction proceeds under green conditions, open to air, at moderate temperatures, yielding moderate to high conversion across a broad range of substrates. The catalyst is easily recovered and can be used in subsequent reactions up to 8 times without a significant decrease in yield.

## Abstract 2

A water-soluble imidazolium-functionalized Cu(ii) catalyst, used in combination with *tert*-butyl hydroperoxide (TBHP), enables benzylic and allylic oxidations in H_2_O.

## Graphical abstract

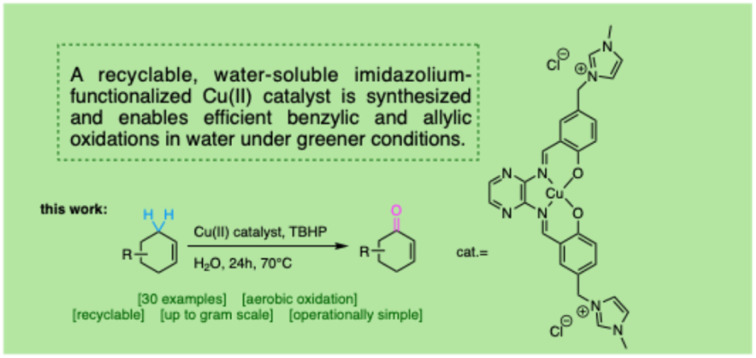

## Introduction

**Scheme 1:**
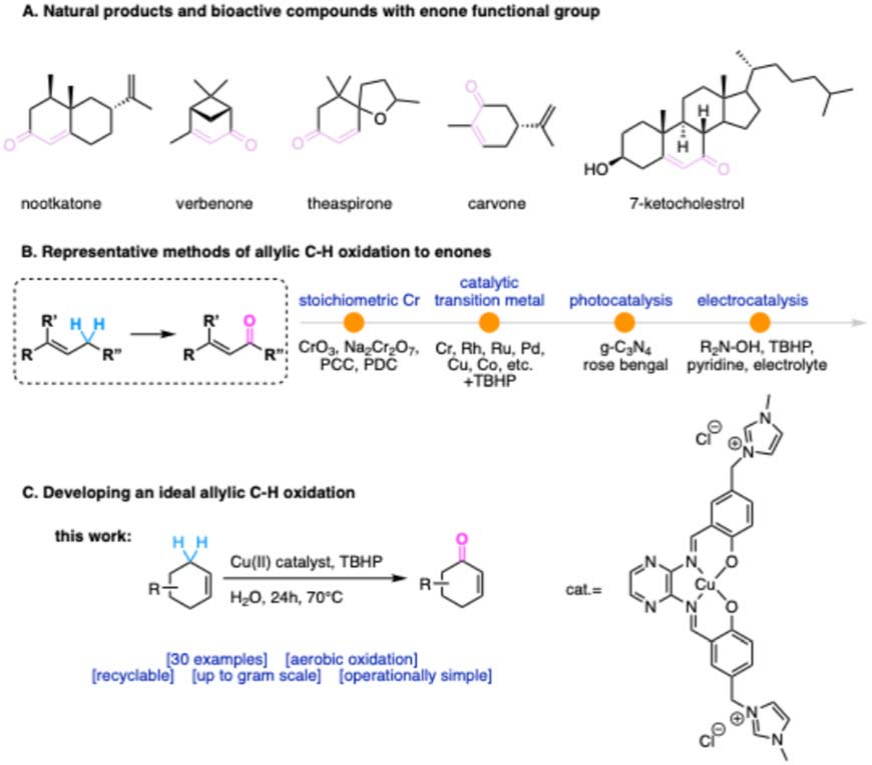
Allylic C–H oxidation to enones.

It has been estimated that roughly 90% of industrial chemical processes involve at least one catalytic step during their synthesis.^1^ The oxidation of an organic substrate, particularly through activation of C–H bonds, accounts for 25% of all the catalytic industrial reactions and is a fundamental transformation in both organic synthesis and biological systems, enabling the conversion of simple hydrocarbons into oxygenated, desaturated, or halogenated compounds.^2–5^ It is crucial to transform readily available bulk materials into value-added products, that serve as necessary feedstocks for the production of everyday goods.^6^ Among these, allylic C–H oxidation to enones has garnered significant attention, for instance, abundant natural products such as steroids and terpenoids feature alkene moieties, and their allylic oxidation products are of great economic and biological importance,^7^ with applications in pharmaceuticals, dietary supplements, and flavorings.^8,9^ (Scheme 1) Traditionally, allylic C–H oxidation to enones has relied on stoichiometric oxidants like CrO_3_ or SeO_2_,^10^ which pose environmental and safety concerns due to the generation of toxic waste during workup.^11–13^ To tackle these challenges, greener alternatives have emerged, such as the use of *tert*-butyl hydroperoxide(TBHP) in combination with transition metal catalysts (*e.g.*, rhodium, ruthenium, palladium, copper, or cobalt).^14–16^ In 2004, Doyle and coworkers reported a catalytic system using dirhodium(ii) caprolactamate, found to be very effective in C–H activation of allylic substrates when coupled with TBHP.^17,18^ Although organocatalytic methods for allylic and benzylic oxidation have been reported,^10,19–22^ metal-catalyzed variants generally exhibit much higher efficiency with cost savings and resource conservation.^23–26^

Similarly, benzyl ketones can also be obtained through C–H oxidation with ubiquitous structures. A recent study by the Lin group (2024) demonstrated that ruthenium carbonyl complexes can efficiently oxidize benzylic compounds using TBHP, offering a broad substrate scope and excellent functional group tolerance.^25^ Moreover, methods that employ allylic or benzylic C–H oxidation as a key step in the synthesis of N-containing heterocycles such as quinoxalines, imidazoles, and oxazoles, *etc.*^27–29^ are widely applied across medicinal, synthetic, and inorganic chemistry.^30^ In 2017, Jeena and co-workers documented the C–H oxidation-mediated synthesis of 2,4,5-trisubstituted imidazoles from α-methylene ketones, aldehydes, and ammonium acetate.^28,29^ However, these C–H activation reactions often require harsh reaction conditions, challenging work-up and purification, and the management of hazardous waste.^30,31^

Previously, in the Gorden group, a complex of Cu(ii) with the 2-quinoxalinol Schiff base ligand has been employed with TBHP for the oxidation of several C–H type bonds, including propargylic,^32^ allylic^33,34^ and benzylic groups.^35^ The extended conjugation in the heteroaromatic backbone stabilizes the high valence metal-oxo species generated during the radical reaction mechanism,^34^ allowing performance comparable to that achievable with the more expensive, toxic, and air-sensitive Ru or Rh catalysts.^10,36–38^ More recently, Cu(ii)-Schiff base complexes have been immobilized on various solid supports to enhance the recyclability and reusability of the catalytic species, such as magnetic-Fe_3_O_4_ nanoparticles for selective oxidation of Anthracene,^38^ carbon nanotubes for oxidation of olefins,^39^ or anchoring of Cu(ii) Schiff base complex in SBA-15 matrix (mesoporous silica) as an efficient processor in alkene oxidation or as a biomimetic catalyst.^40^ In a new report from 2024, Tang *et al.* demonstrated a method of sustainable aerobic allylic C–H bond oxidation with a heterogeneous iron catalyst.^41^ Overall, the use of Cu(ii) or other metals on solid supports offers notable advantages, but they still face challenges,^42^ such as the synthesis and proper characterization of supported catalysts, which often require multistep procedures, making it technically challenging to ensure uniform distribution and characterize active-site integrity.^43,44^

**Scheme 2:**
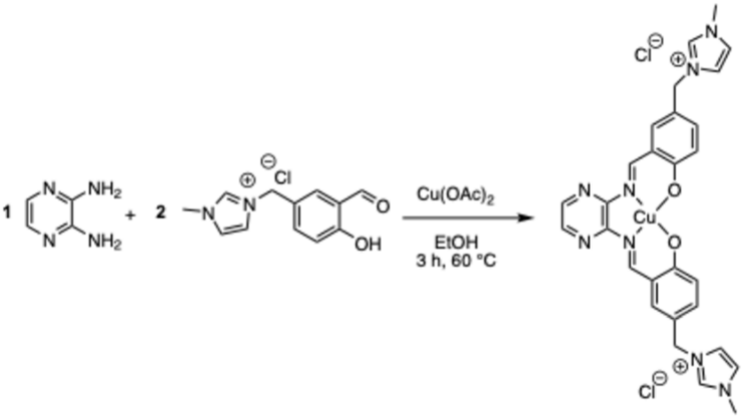
One-pot synthesis of the Cu(ii) complex.

Based on the above, the ideal allylic C–H oxidation process should fulfill several key criteria: (1) utilize a green, inexpensive oxidant that is environmentally benign in both its preparation and application. (2) leave minimal metal residues in the final products, particularly critical for pharmaceuticals and food additives; (3) employ cost-effective and reusable catalytic systems; and (4) proceed under mild conditions with a simple operational setup. In response to these demands, here we report a highly sustainable allylic and benzylic C–H oxidation protocol utilizing a stable and recyclable Cu(ii) catalyst, a novel complex of Cu(ii) with an imidazolium-functionalized pyrazine Schiff base analogous to salophen.^43^ Following the principles of green chemistry,^44–50^ this system achieves efficient synthesis of enones and benzylic ketones using TBHP as the terminal oxidant. The method exhibits a broad substrate scope and is supported by detailed recycling studies (Scheme 2).

## Experimental section

### Material and instruments

All commercially available reagents and solvents were used as received and without further purification. Chromatographic purifications were performed using Fischer (60 Å, 70–230 mesh) silica gel. NMR spectra were recorded on a Jeol 400 mHz instrument. GC-MS analysis was performed on an Agilent Intuvo 9000 GC/5977 MS system.

### Synthesis of imidazolium-functionalized salicylaldehyde (compound 1)

To 150 mL of concentrated hydrochloric acid was added 15 mL (141 mmol) of salicylaldehyde and 12 mL (161 mmol) of formaldehyde. The resulting solution was stirred at room temperature for 24 hours. The precipitate obtained was filtered and washed with a saturated Na_2_CO_3_ solution until the pH of the filtrate reached 8, then with distilled water. After recrystallization with hexanes, the obtained 5-chloromethyl salicylaldehyde (2 g, 11.72 mmol) was dissolved in acetonitrile until a clear solution was formed, and then, 1.15 g (14 mmol, 1.2 eq.) of 1-methyl imidazole was added dropwise under an argon atmosphere. After stirring at room temperature for 3 hours, the solids were filtered, washed with acetonitrile, and dried in a vacuum oven (yield 91%, 2.7 g).

^1^H NMR (400 MHz, D2O) *δ* 9.77 (d, *J* = 2.1 Hz, 1H), 7.61 (t, *J* = 2.7 Hz, 1H), 7.44 (dd, *J* = 8.5, 2.1 Hz, 1H), 7.33–7.25 (m, 2H), 6.93–6.86 (m, 1H), 5.21 (s, 2H), 3.71 (s, 4H). 13C NMR (101 MHz, D_2_O) *δ* 196.77, 160.33, 137.47, 136.05, 133.64, 125.59, 123.92, 122.13, 121.15, 118.17, 51.79, 35.79.

### Synthesis of Cu-IL-pyrasal

To a round-bottom flask containing 10 mL of ethanol were added 8.75 mmol of compound 1 (2.25 g), 4.37 mmol of 2,3-diaminopyrazine (481 mg), and 4.81 mmol of copper(ii) acetate (960 mg). The mixture was stirred for 4 hours at reflux temperature. The dark solid formed was filtered, washed with ice-cold ethanol, then dried in a vacuum oven to afford the metal complex with 72% yield (2.02 g) as a brown solid.

HRMS: *m*/*z* calc. for C_28_H_26_CuN_8_O_2_^2+^ 284.57, found 284.5742.

### X-ray crystallography

Data were collected on a Rigaku XtaLAB Synergy-*i* Kappa diffractometer equipped with a PhotonJet-*i* X-ray source operated at 50 W (50 kV, 1 mA) to generate Cu Kα radiation (*λ* = 1.54178 Å) and a HyPix-6000HE HPC detector. Crystals were transferred from the vial onto a glass slide using Cargille type NVH immersion oil. A Zeiss Stemi 305 microscope was used to identify a suitable specimen for X-ray diffraction from a representative sample of the material. The crystal and a small amount of the oil were collected on a 100-micron MiTeGen CryoLoop and transferred to the instrument, where they were placed under a cold nitrogen stream (Oxford 700 series) maintained at 100 K throughout the duration of the experiment. The sample was optically centered with the aid of a video camera to ensure no translations were observed as the crystal was rotated through all positions. The file CCDC 2480695 contains the crystallographic data for this paper. This data can be accessed free of charge on www.ccdc.cam.ac.ul/data_request/cif, or by emailing data_request@ccdc.cam.ac.uk, or by contacting The Cambridge Crystallographic Data Centre, 12 Union Road, Cambridge CB2 1EZ, UK; fax: +44 1223 336033.

### Refinement details (SQUEEZE)

After data collection, the unit cell was re-determined using a subset of the full data collection. Intensity data were corrected for Lorentz, polarization, and background effects using the *CrysAlis*^*Pro*^.^51,52^ A numerical absorption correction was applied based on a Gaussian integration over a multifaceted crystal and followed by a semi-empirical correction for adsorption applied using the program *SCALE3 ABSPACK*.^53^ The programs *SHELXT*^54^ was used for the initial structure solution and *SHELXL*^55^ was used for refinement of the structure. All these programs were utilized within the OLEX2 software.^56^ Hydrogen atoms bound to the carbon and nitrogen atoms were in the difference Fourier map, where possible, and were geometrically constrained using the appropriate AFIX commands.

### General procedure for allylic oxidation in water

In a screw-cap vial, Cu-IL-pyrasal (2 mol%, 12 mg) was dissolved in 1 mL of distilled water. Then, 1 mmol of the allylic substrate and 3 equivalents of *t*-butyl hydroperoxide (70% aqueous solution, 3 mmol) were added, and the mixture was stirred at 70 °C for 24 hours. The organic products were extracted with ethyl acetate, dried with anhydrous MgSO_4_, and then the solvent was removed under vacuum, and the mixture was analyzed *via* GC-MS.

### General procedure for benzylic oxidation in water

In a screw-cap vial, Cu-IL-pyrasal (2 mol%, 12 mg) was dissolved in 1 mL of distilled water. Then, 1 mmol of benzylic substrate and 6 equivalents of *t*-butyl hydroperoxide (70% aqueous solution, 6 mmol) were added to the mixture. Then, 1 mmol of benzylic substrate and 6 equivalents of *t*-butyl hydroperoxide (70% aqueous solution, 6 mmol) were added, and the mixture was stirred at 70 °C for 24 hours. The organic products were extracted with ethyl acetate, dried with anhydrous MgSO_4_, then the solvent removed under vacuum. The crude mixture was analyzed *via* GC-MS.

### General procedure for gram-scale benzylic oxidation

To a solution of Cu-IL-pyrasal (2 mol%, 72 mg), was added 1 gram of diphenylmethane (6 mmol) and 6 equivalents of TBHP. After stirring for 24 hours at 70 °C, the organic products were extracted with ethyl acetate and dried with anhydrous MgSO_4_. The crude mixture was purified *via* silica gel column chromatography (hexanes/ethyl acetate 30 : 1, 10 : 1), to afford 907 mg (83% yield) of benzophenone as an off-white solid. *R*_f_ = 0.71 (hexanes/ethyl acetate, 30 : 1)

^1^H NMR (400 MHz, chloroform-*d*) *δ* 7.87–7.73 (m, 2H), 7.64–7.50 (m, 1H), 7.47 (dd, *J* = 8.2, 6.8 Hz, 2H).

^13^C NMR (101 MHz, chloroform-*d*) *δ* 196.87, 137.67, 132.54, 130.17, 128.39.

### General procedure for catalyst recycling

In a screw-cap vial, Cu-IL-pyrasal (2 mol%, 12 mg) was dissolved in 1 mL of distilled water, then 1 mmol of diphenylmethane (168 µL) and 6 equivalents of *t*-butyl hydroperoxide (70% aq. solution, 6 mmol) were added, and the mixture was stirred at 70 °C for 24 hours. The organic phase was extracted with ethyl acetate, dried over anhydrous MgSO4, and the solvent evaporated under vacuum. The resulting extract was then analyzed *via* GC-MS. The aqueous phase containing the Cu(ii) catalyst was used in successive reactions by adding 1 mmol of diphenylmethane and 6 mmol of *t*-butyl hydroperoxide.

## Results and discussion

### Synthesis of ligand and Cu(ii)-complex

The ionic imidazolium-functionalized aldehyde required for the synthesis of the Schiff base was prepared according to a previously published procedure.^43,57^ The partner 2,3-diaminopyrazine was chosen to mimic the heteroatomic nature of highly active, similar complexes while not requiring additional synthetic steps. The condensation reaction between the aldehyde and the diamine did not afford the desired ligand product. The imine or other intermediate formed may be unstable in solution and prone to hydrolysis. The positive charge on the imidazolium aldehyde makes ligand formation reversible and unfavorable unless a metal ion coordinates to it. Therefore, we opted for a templated one-pot synthesis of the Cu(ii) complex. Mixing and heating in ethanol, the 2,3-diaminopyrazine with two equivalents of imidazolium-functionalized aldehyde, and Cu(ii) acetate formed a precipitate, which was filtered and washed (Refer to 54 above).

### X-ray structure of Cu(ii)-IL-pyrasal complex

**Fig. 1:**
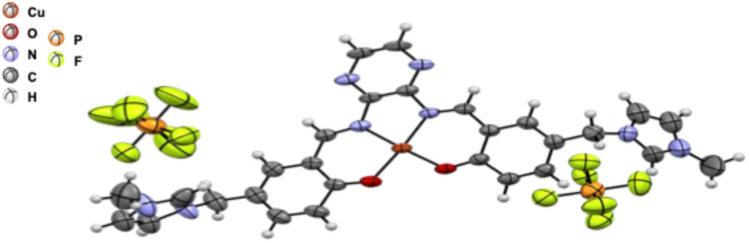
The single crystal XRD of Cu(ii)-IL-pyrasal complex as (C_28_H_26_CuN_8_O_2_^+^·2PF_6_^−^); details: the central Cu(ii) metal ion is shown as dark orange, and the others are shown as above. The F^−^ atoms are bright yellow and surround the orange P_5_^+^ atom in the stabilizing PF_6_^−^ counter ions.

The X-ray crystal structure in Fig. 1 revealed a Cu(ii)-IL-pyrasal complex with the molecular formula C_28_H_26_CuN_8_O_2_^2+^·2PF_6_^−^, in which the Cu(ii) center is coordinated by two nitrogen-containing ligands and two oxygen donors, creating a nearly square planar geometry. Such N, N, O, O coordination stabilizes the Cu(ii) oxidation state while allowing for facile redox cycling between Cu(ii) and Cu(i), a key feature for catalytic activity during the allylic oxidation. The oxygen donors further modulate the electron density around the Cu center, enhancing its ability to bind TBHP (as a co-oxidant) and favoring the selective activation of allylic C–H bonds while suppressing over-oxidation, thereby strengthening the observed recyclable catalytic activity and selectivity. However, –Cl ions have poor crystallization behavior because Cu salts with small, strongly coordinating anions (–Cl, –Br, –NO_3_) often pack tightly and form poorly diffracting or microcrystalline solids. selectivity. In this work, replacing –Cl with –PF_6_, the bulky, weakly coordinating anion does not bind strongly to the Cu center. By contrast, two –PF_6_ moieties stay far from the coordination sphere, preserving the true cationic structure intact and making it easier to precipitate out of its solution form.

The view of the coordination geometry of the complex shown in Table 1. The four O–Cu–O, O–Cu–N, and N–Cu–N angles differ only slightly from 90° and the two O–Cu–N angles are close to 175°, which suggests that the N_2_O_2_ set of donor atoms binds the copper in a nearly square-planar geometry. The two Cu–O distance are 1.894 Å and 1.887 Å and the Cu–N distance are 1.943 Å and 1.941 Å, respectively. These bond distances of this complex are comparable (±0.003 Å) to the similar Cu(ii) salen or salophen complex reported previously.^58–61^ The metal–metal distance between the neighboring complex unit is significant, 4.248 Å, such that intermolecular interaction can be excluded. Overall, the Cu(ii) center sits nearly planar and approximately equidistant from the four donor atoms, consistent with a stable square-planar N_2_O_2_ coordination and a complementary coordination pocket size.^62–64^

**Table 1:** Selected bond distance and angles for the complex of Cu-IL-pyrasal

Bond length (Å)		Bond angles(°)	
Cu(1)–O(1)	1.893(3)	O(1)–Cu(1)–O(2)	88.2(1)
Cu(1)–O(2)	1.887(3)	O(1)–Cu(1)–N(3)	93.6(1)
Cu(1)–N(3)	1.943(3)	O(2)–Cu(1)–N(6)	93.6(1)
Cu(1)–N(6)	1.941(3)	N(3)–Cu(1)–N(6)	84.9(1)
		O(1)–Cu(1)–N(6)	174.1(1)
		O(2)–Cu(1)–N(3)	176.7(1)

**Scheme 3:**
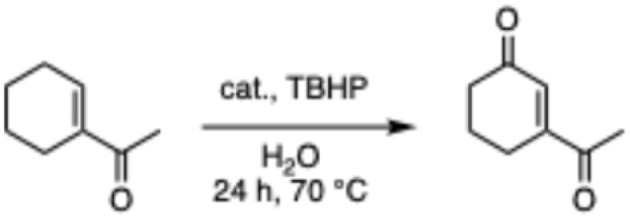
Oxidation of allylic substrate in H_2_O.

The screening of the catalytic activity of the copper complex began with testing the optimal loading of the metal complex. The oxidation of the acetyl cyclohexene substrate was carried out on a millimole scale, using 1 mL of water as the solvent and TBHP (3 equivalents) as the oxidizing agent (Scheme 3) to initiate the optimization.

**Fig. 2:**
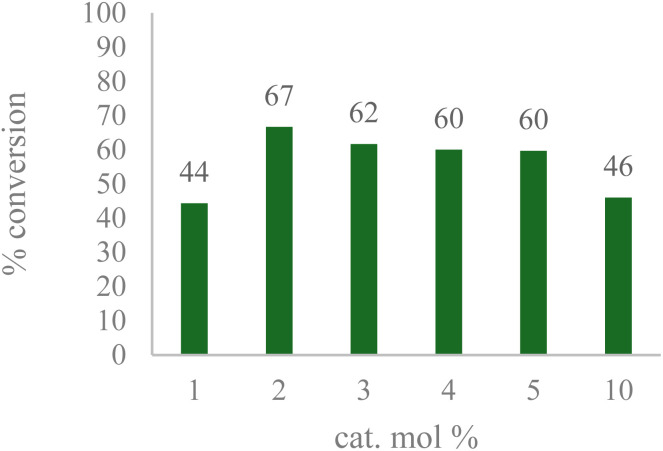
Catalyst optimization; reaction conditions: 1 mmol 1-acetyl-1-cyclohexene, three eq. TBHP, 1 mL H_2_O, 70 °C, 24 h; conversion determined *via* GC-MS based on remaining starting material.

**Fig. 3:**
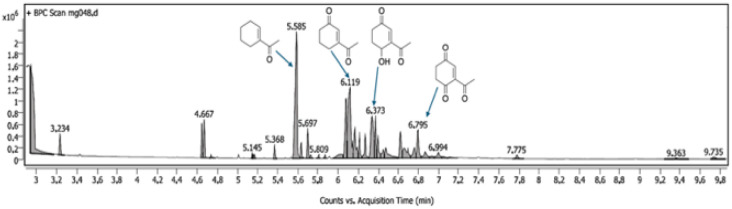
GC analysis of the crude reaction mixture showing secondary oxidation products.

The results shown in Fig. 2 indicate a maximum conversion of 67% when 2 mol% of the metal complex was employed. In contrast, the amount of the desired ketone product decreased as more catalyst was introduced. This can be explained by GC-MS analysis of the crude reaction mixture, in which the peaks of byproducts can be observed with masses matching those of secondary oxidation products (Fig. 3).

**Fig. 4:**
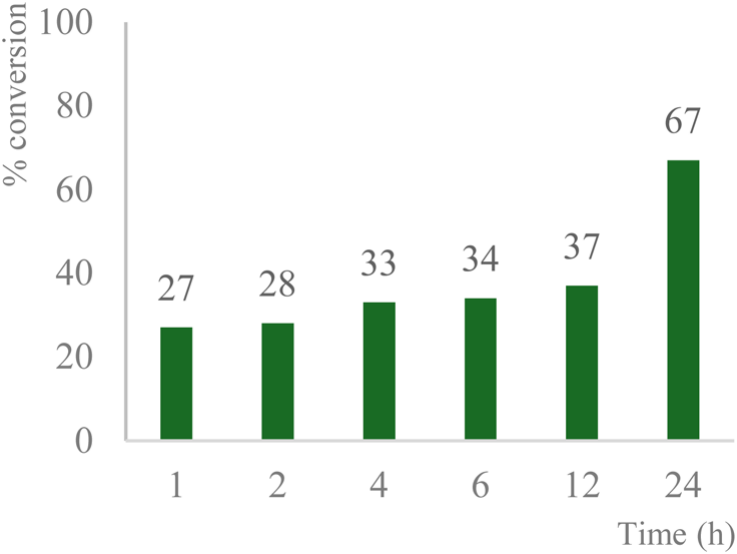
Effect of time on product formation; reaction conditions: 1 mmol 1-acetyl-1-cyclohexene, 2 mol% catalyst, 3 eq. TBHP, 1 mL H_2_O, 70 °C, 24 h; conversion determined *via* GC-MS based on the remaining starting material.

**Scheme 4:**
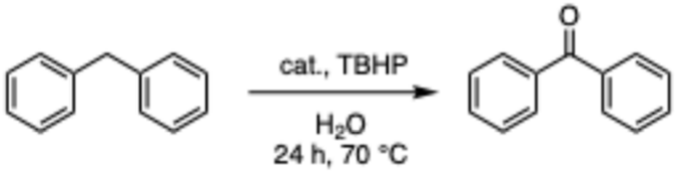
Oxidation of diphenylmethane to benzophenone in H_2_O.

**Fig. 5:**
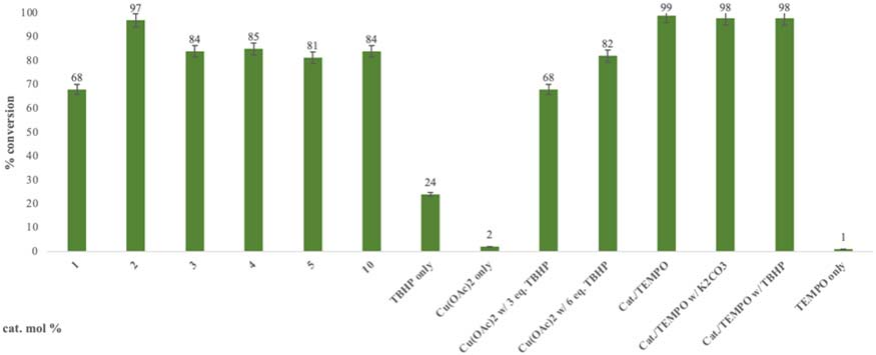
Catalyst optimization; reaction conditions: 1 mmol diphenylmethane, 6 eq. TBHP, 1 mL H_2_O, 70 °C, 24 h; compared to Cu(OAc)2 salt and TEMPO oxidations (5 mol% TEMPO, 2 mol% Catalyst), conversion determined *via* GC-MS based on the remaining starting material.

The 24-hour reaction time, in conjunction with vigorous stirring, was initially chosen to maximize contact between the immiscible catalyst/TBHP water solution and the organic substrates. This was confirmed by performing the oxidation at different times (Fig. 4). Comparing this to previous and work in acetonitriles with a similar catalyst^57^ in aqueous solution the reaction takes longer as the system is non-homogenous and the product can only be formed at the intersection of the two media. In the case of benzylic oxidation, the same conditions were used, but the amount of TBHP added was doubled (6 equivalents). The optimization of the reaction showed that diphenylmethane (Scheme 4) afforded the maximum amount of product with only 2 mol% catalyst loading. These results were compared to Cu(OAc)_2_ salt and TEMPO (2,2,6,6-tetramethylpiperidine 1-oxyl), another free radical scavenger commonly used in benzylic oxidations^65^ (Fig. 5).

The scope of the catalyst was initially tested in the oxidation of allylic substrates (Table 2). A quantitative transformation was obtained with simple cyclohexene (entry 1), and the yield was high. A quantitative transformation was achieved with simple cyclohexene (entry 1), and high yields were observed for compounds containing electron-donating groups, such as 1-methyl and 1-phenyl substituents (entries 2 and 4). At the same time, the 3-methyl equivalent showed a decrease in product (entry 3). On the other hand, substrates carrying electron-withdrawing groups exhibited poor reactivity, affording only trace amounts of products in the case of nitro and nitrile-substituted cyclohexenes (entries 8 and 9). No product formation was observed for indene (entry 10). At the same time, while 1-cyclohexenyl acetate underwent hydrolysis instead of oxidation due to the presence of excess water as the solvent and the slightly acidic reaction environment, the catalyst was found to promote hydrolysis affording an alcohol as the product (entry 6). Structures with larger and smaller rings were also tested (entries 11–13) and yielded high conversions, demonstrating the overall applicability of the catalytic system to various substrate types.

**Table 2:** Substrate scope for allylic oxidation; reaction conditions: 1 mmol substrate, 2 mol% Cu-IL-pyrasal, 3 eq. TBHP, 1 mL H_2_O, 70 °C, 24 h; conversion determined *via* GC-MS based on the remaining starting material

Entry	Substrate	Product	Conversion (%)
1	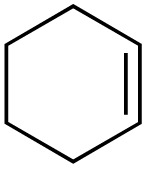	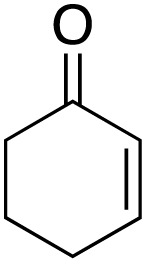	99
2	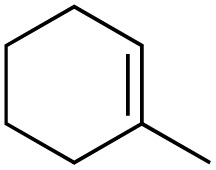	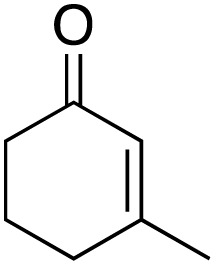	89
3	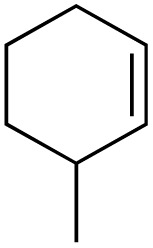	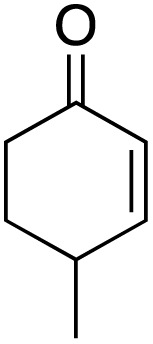	56
4	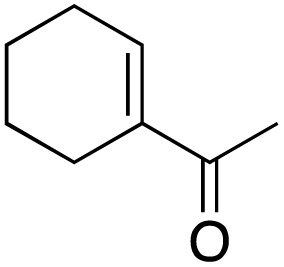	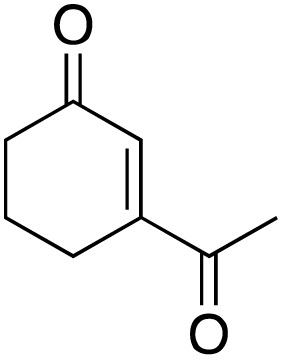	67
5	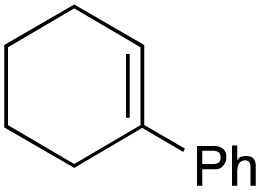	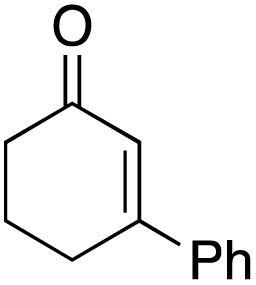	76
6	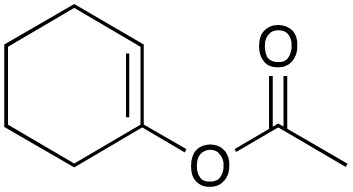	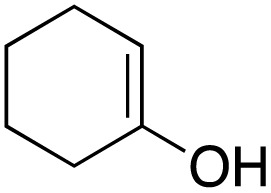	21
7	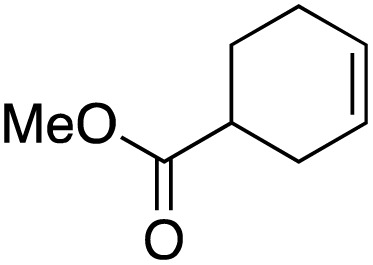	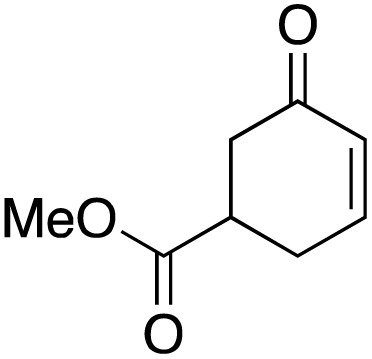	65
8	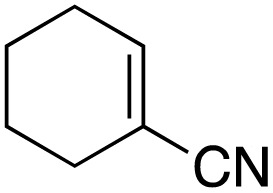	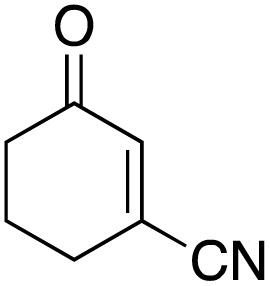	Trace
9	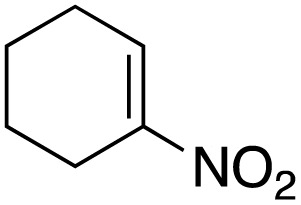	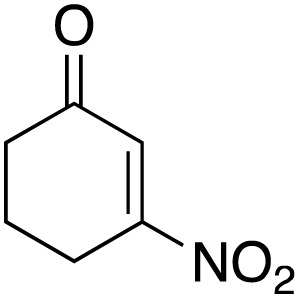	Trace
10	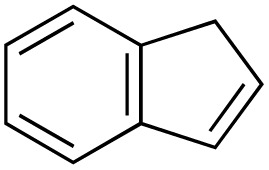	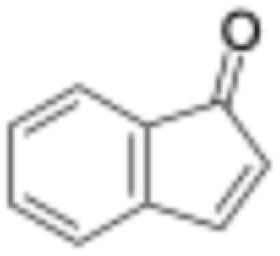	Not detected
11	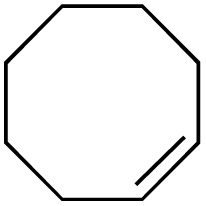	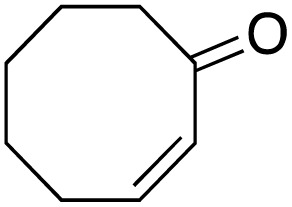	73
12	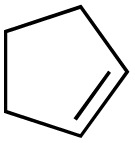	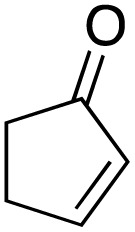	99
13	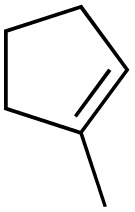	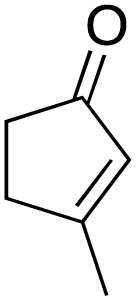	60

A diverse range of compounds was employed in the oxidation of benzylic substrates, displaying high to moderate activity and tolerance for a variety of functional groups (Table 3). For molecules only containing aliphatic chains, the amount of desired product decreased when increasing the chain length (entries 1–3), and there was no over-oxidation reaction detected even up to the gram scale. The conversion appears to increase in the presence of halogen (entries 4–7) or electron-withdrawing substituents on the benzene (entry 10). High conversion is also observed with fully aromatic substrates (diphenylmethane and 2-ethyl naphthalene, entries 8 and 11), whereas it decreases for the ester product derived from the methoxy substrate (entry 9). An interesting result was obtained in the reaction with ethyl phenylacetate, where, in addition to the oxidation, the ester substrate underwent hydrolysis to afford the alpha-keto acid (entry 12). Because of their importance as building blocks^65^ for drug synthesis, a series of nitrogen-containing compounds was explored: good results were obtained with benzylamine and pyridine derivatives (entries 13, 14, 16), and an exceptional 98% conversion was observed for 2-picolylamine (entry 19), while no product was detected for its *N*,*N*-dimethyl derivative, and N-benzyl acetamide (entries 20 and entry 15). Complete aromatization was observed with 1,2,3,4-tetrahydroquinoline (entry 23). Almost complete conversion was observed using xanthene (entry 21), while dihydroanthracene was oxidized to a diketone (entry 22).

**Table 3:** Substrate scope for benzylic oxidation; reaction conditions: 1 mmol substrate, 2 mol% Cu-IL-pyrasal, 6 eq. TBHP, 1 mL H_2_O, 70 °C, 24 h; conversion determined *via* GC-MS based on the remaining starting material

Entry	Substrate	Product	Conversion(%)
1	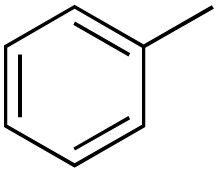	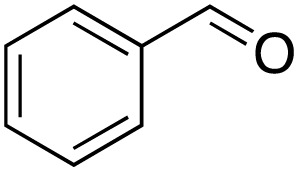	99
2	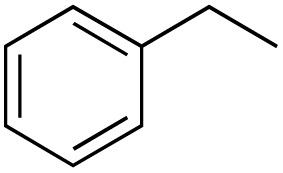	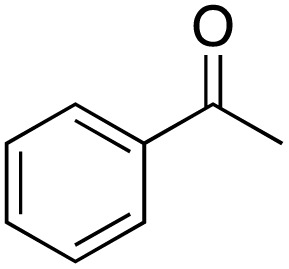	81
3	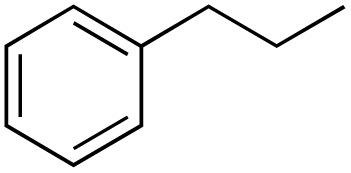	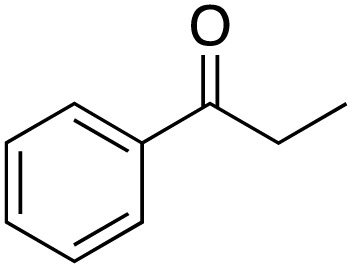	47
4	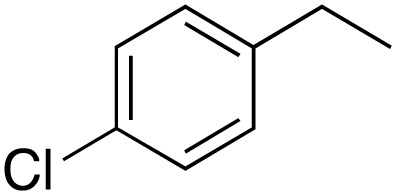	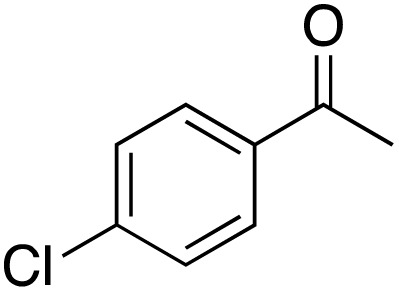	88
5	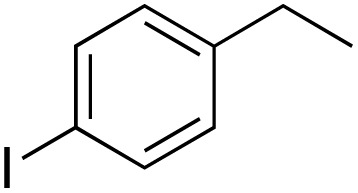	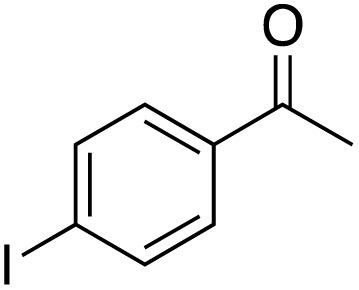	89
6	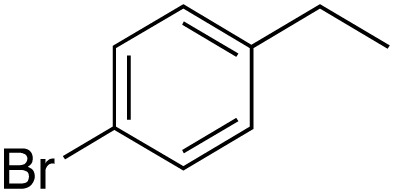	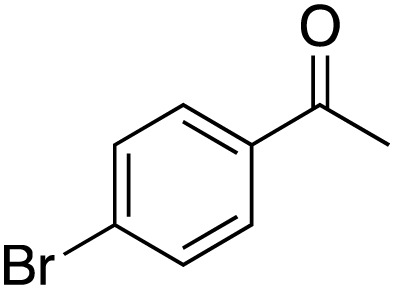	92
7	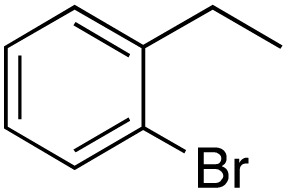	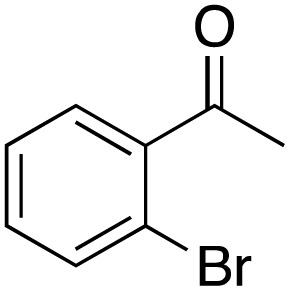	80
8	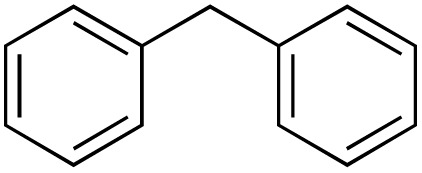	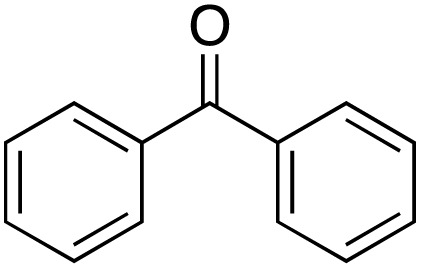	88
9	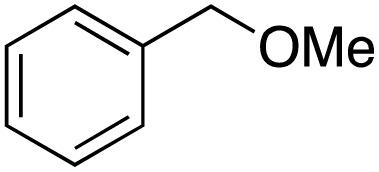	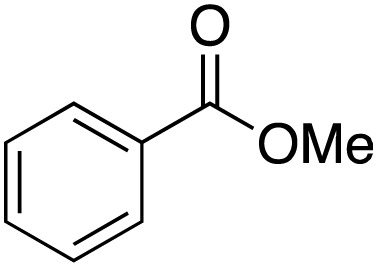	38
10	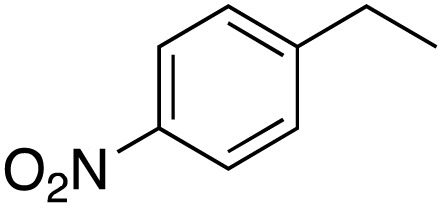	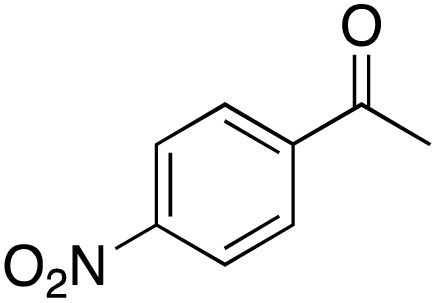	88
11	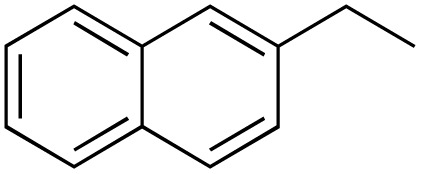	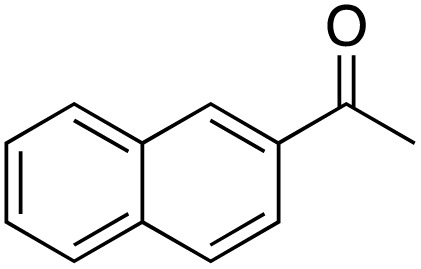	86
12	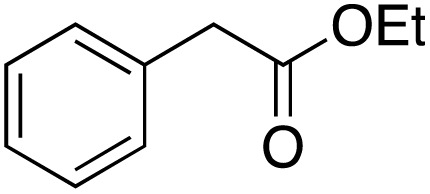	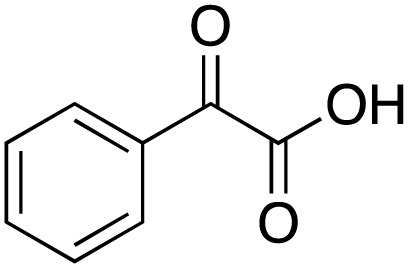	60
13	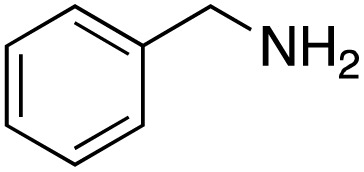	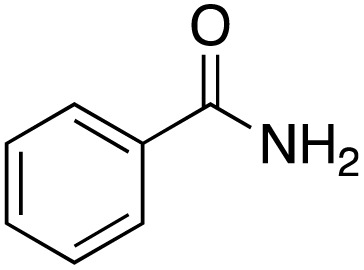	91
14	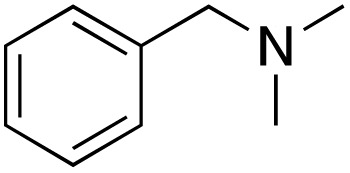	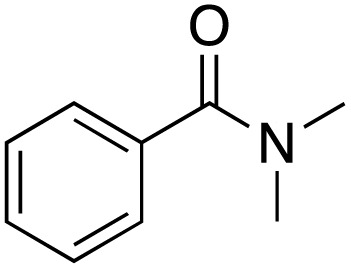	73
15	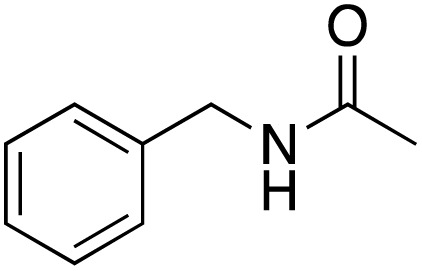	No product	**—**
16	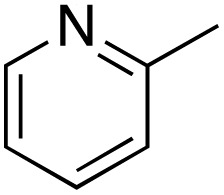	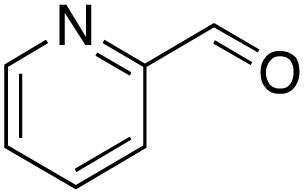	97
17	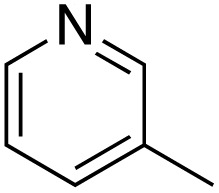	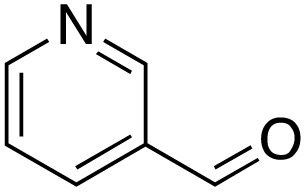	>1%
18	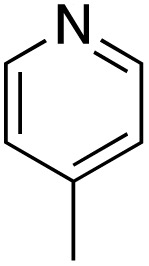	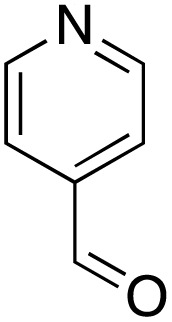	28
19	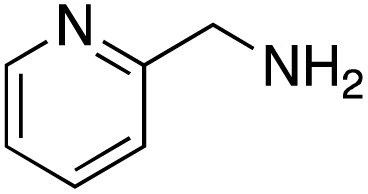	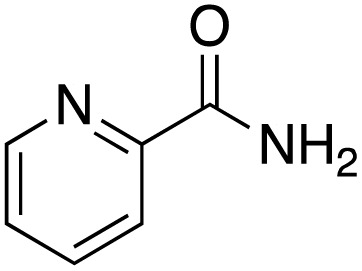	98
20	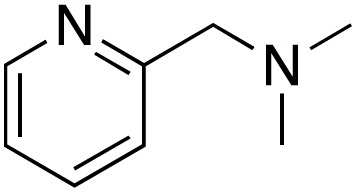	No product	**—**
21	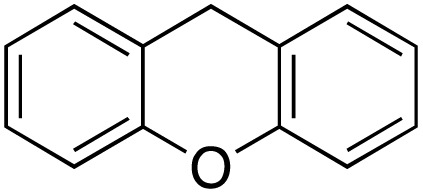	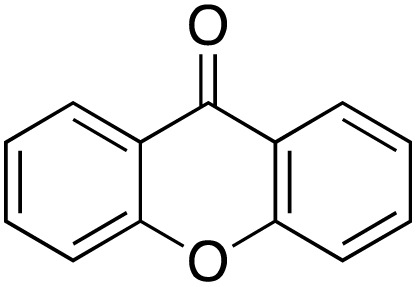	98
22	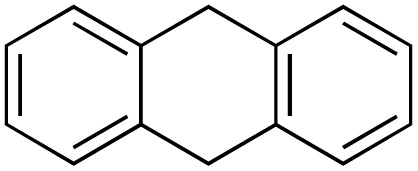	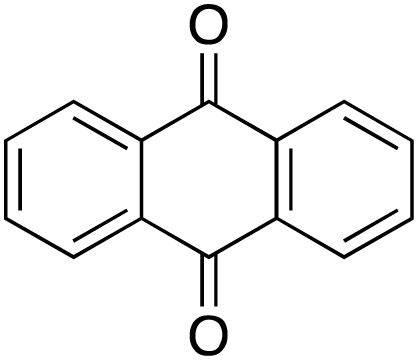	75
23	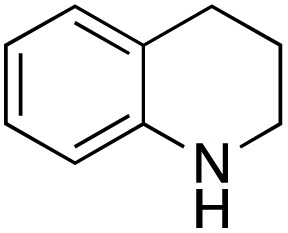	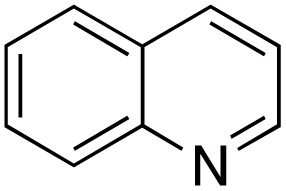	99

**Fig. 6:**
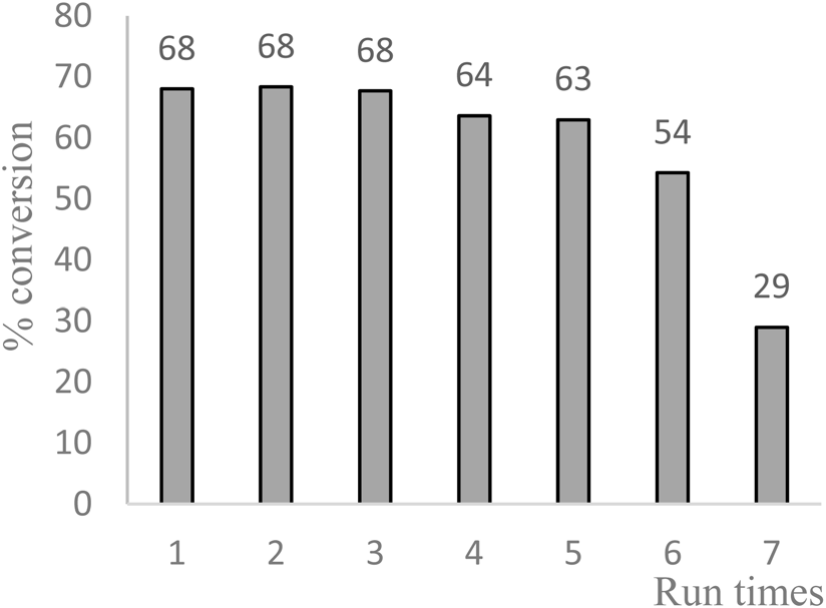
Recycling of Cu-IL-pyrasal in allylic oxidation; reaction conditions: 1 mmol acetyl cyclohexene, 2 mol% Cu-IL-pyrasal, 3 eq., TBHP, 1 mL water, 70 °C, 24 hours, conversion determined by GC-MS.

**Fig. 7:**
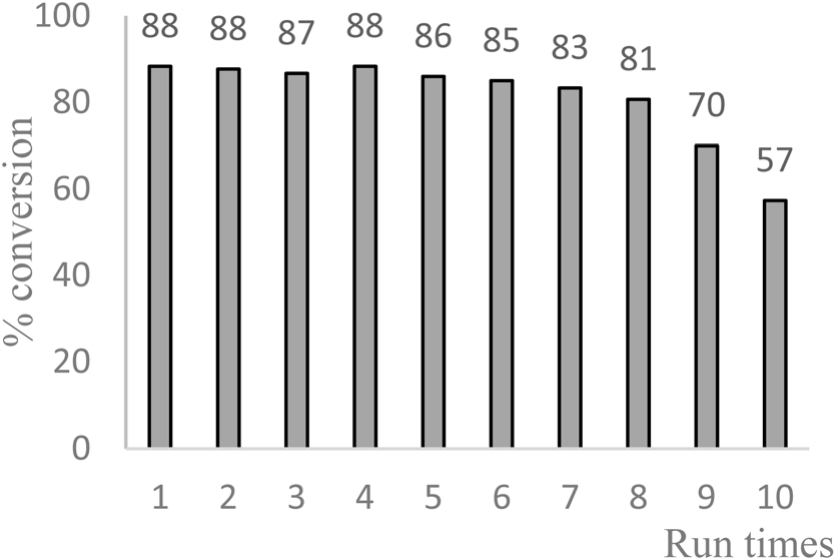
Recycling of Cu-IL-pyrasal in benzylic oxidation: reaction conditions: 1 mmol diphenylmethane, 2 mol% Cu-IL-pyrasal, 6 eq., TBHP, 1 mL water, 70 °C, 24 hours. Conversion was determined by GC-MS.

Benzylic oxidation with Cu-IL-pyrasal was also carried out at the gram scale. Complete product recovery was observed from the reaction of diphenylmethane in water, and the yield of 83% is comparable to that determined by GC analysis for micro-scale reactions. Finally, the recyclability of the catalytic system was explored. After a typical reaction, the products are separated from the metal complex by liquid–liquid extraction. To the aqueous phase containing the catalyst, fresh TBHP and substrate are added. The aqueous phase containing the catalyst is then treated with fresh TBHP and substrate, and the reaction is repeated. Fig. 6 shows that the activity of the Cu complex starts decreasing after 5 consecutive reactions when used for allylic oxidation; in contrast, up to 8 repetitions are sustained in the benzylic oxidation (Fig. 7).

## Conclusions

In conclusion, we report the synthesis of a water-soluble Cu(ii) complex derived from an imidazolium-functionalized salen ligand and its application in oxidative reactions. The complex was obtained by a one-pot template reaction and isolated by filtration, reducing synthetic steps, energy input, and waste generation. After optimizing the reaction conditions in water, it was found that a 2 mol% catalyst loading is effective in the C–H activation of a variety of allylic and benzylic substrates using TBHP as the oxidant. The system represents a valid method for introducing oxygen functionalities into otherwise non-reactive molecules, affording moderate to high conversion of aldehydes, ketones, esters, and N-containing products in one step, even at the gram scale. Additionally, the Cu(ii) complex can be reused up to eight times without significant loss of activity, improving the sustainability of the process.

From a green chemistry perspective, the use of water as a solvent, recyclable catalyst design, and mild reaction conditions align well with the principles of the recently proposed ETR framework, which emphasizes resource efficiency and environmental compatibility in catalytic processes.^66^ In our system, copper serves as an abundant and benign source of copper, TBHP provides a clean driving force, and the oxygenated products obtained in high yields reflect an efficient reaction output. These characteristics highlight the potential of this system for industrial-scale sustainable oxidation, where controllable selectivity and catalyst recyclability are essential. Looking forward, integrating ETR-guided design principles with rational ligand development could further advance the creation of eco-efficient oxidation catalysts for large-scale green synthesis. Furthermore, TEMPO testing in benzylic oxidations proved effective and may provide a better alternative to TBHP in this reaction for future work.

## Conflicts of interest

The authors have no financial interests to declare.

## Supplementary Material

RA-016-D5RA09017B-s001

RA-016-D5RA09017B-s002

## Data Availability

The datasets such as NMR data and catalyst complex characterization supporting this article have been uploaded as part of the supplementary information (SI). Other data – such as specific mass spectroscopy data – generated during and/or analyzed during the current study are not publicly available as they are interpreted in the catalytic yields, but are available from the authors on reasonable request. CCDC 2480695 contains the supplementary crystallographic data for this paper.^67^ Supplementary information (SI): characterization of the catalyst complex, including ^1^H NMR, UV-Vis, and HRMS; ^1^H NMR characterization of isolated gram-scale oxidation product and GC-MS data for all of the oxidation products. See DOI: https://doi.org/10.1039/d5ra09017b.
