# Supplementary material for: Water-soluble imidazolium-functionalized Cu(ii) complex as a recyclable catalyst for allylic and benzylic oxidation
Source: RSC Adv. 2026 Jan 5;16(2):1335–43. doi: 10.1039/d5ra09017b (PMC12768404; doi:10.1039/d5ra09017b)
Supplement: RA-016-D5RA09017B-s001 [file RA-016-D5RA09017B-s001.pdf]

Supplementary Information (SI) for RSC Advances.
This journal is © The Royal Society of Chemistry 2025

Water-soluble imidazolium-functionalized Cu(II) complex as a recyclable catalyst for allylic and benzylic oxidation

[1]Xiaoyu Huo, [1]Mariano Guagliardo, Xander Duke, Bailey Bouley and Anne E.V. Gorden*

Department of Chemistry and Biochemistry,
Texas Tech University,
Lubbock, Texas 79415, United State

Supporting Information

Contents

Characterization of new Cu(II)-IL-pyrasal complex

a) ^{1}H NMR of IL-Tagged Salicylaldehyde
b) HR-MS: **Figure 1**
c) IR Spectra: **Figure 2**
d) UV-Vis
➢ **Figure 3**
➢ **Figure 4**

Description of the crystallization of Cu(II)-IL-pyrasal crystal

a) **Figure 6.** The X-Ray of the complex molecule $C_{28}H_{26}CuN_8O_2^+ \cdot 2PF_6^-$
b) Synthesis method and Crystal growing steps
c) Refinement details: **Table 1**
d) References
e) Bond length and angel

^{1}H NMR technique for characterization of oxidation products

a) Characterization of isolated gram-scale oxidation products
b) Scanned copies of ^{1}H NMR

GC-MS Data for characterization of oxidation products

a) Scanned copies of Benzylic oxidation GC/MS reports
b) Scanned copies of Allylic Oxidation GC/MS reports

Instrumentation:

^{1}H-NMR and ^{13}C-NMR were recorded on JEOL ECS 400 MHz and 500MHz NMR spectrometer operating at 298 K. ^{1}H NMR spectra were referenced internally to the solvent resonances. NMR samples were dissolved in DMSO-d_6 as noted. The detection of Copper(II) complexes is hindered by Cu(II) paramagnetic properties.

Solid-state IR spectra were collected on a Thermo Scientific Nicolet iS10 instrument equipped with a diamond ATR.

Solution-phase UV-vis data were obtained in 20 μM solutions using an Agilent Technologies Cary Series UV-vis Spectrophotometer. The samples were dissolved in methanol and placed in a 1 cm quartz cell. Data were collected from 200 to 800 nm.

GC–MS analyses were performed on an Agilent Intuvo 9000 GC/5977 MS system.

1H NMR (400 MHz, D2O) δ 9.77 (d, J = 2.1 Hz, 1H), 7.61 (t, J = 2.7 Hz, 1H), 7.44 (dd, J = 8.5, 2.1 Hz, 1H), 7.33 – 7.25 (m, 2H), 6.93 – 6.86 (m, 1H), 5.21 (s, 2H), 3.71 (s, 4H).

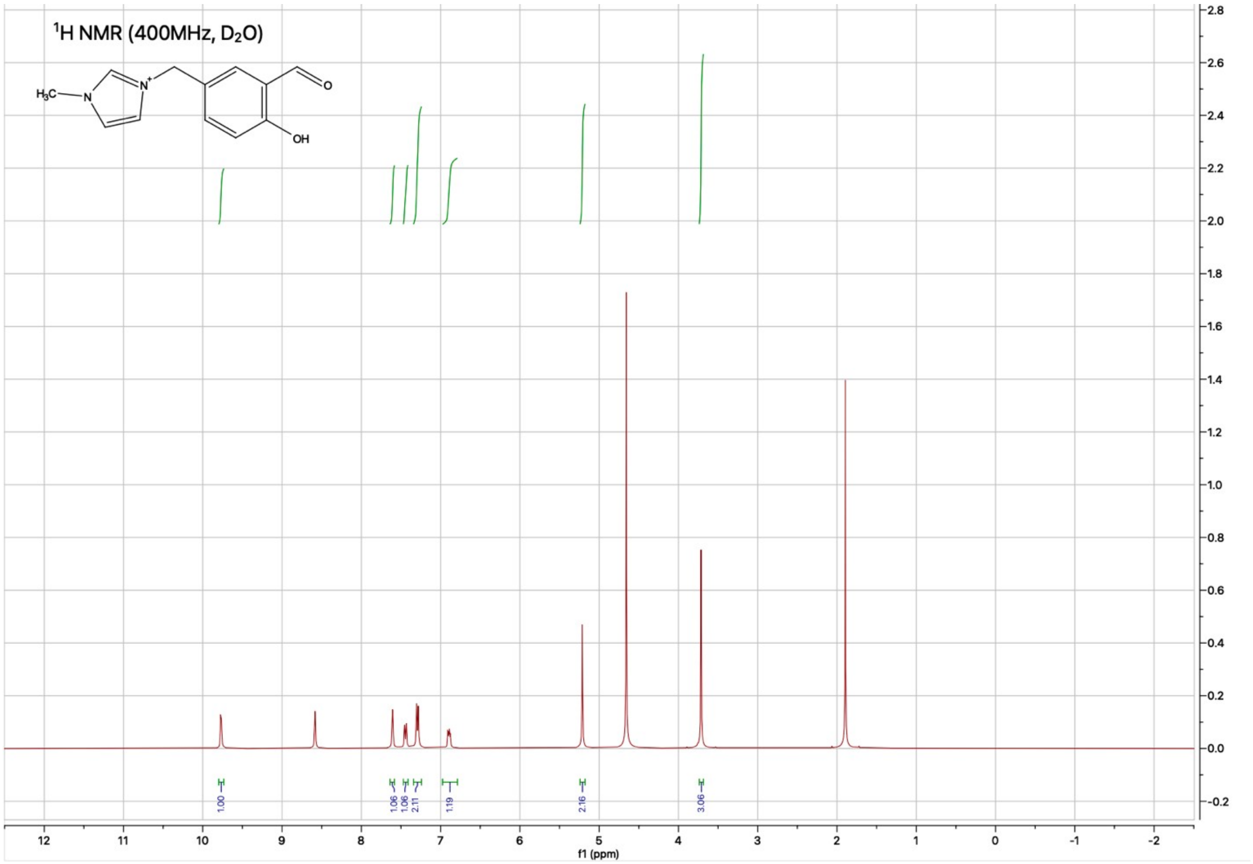

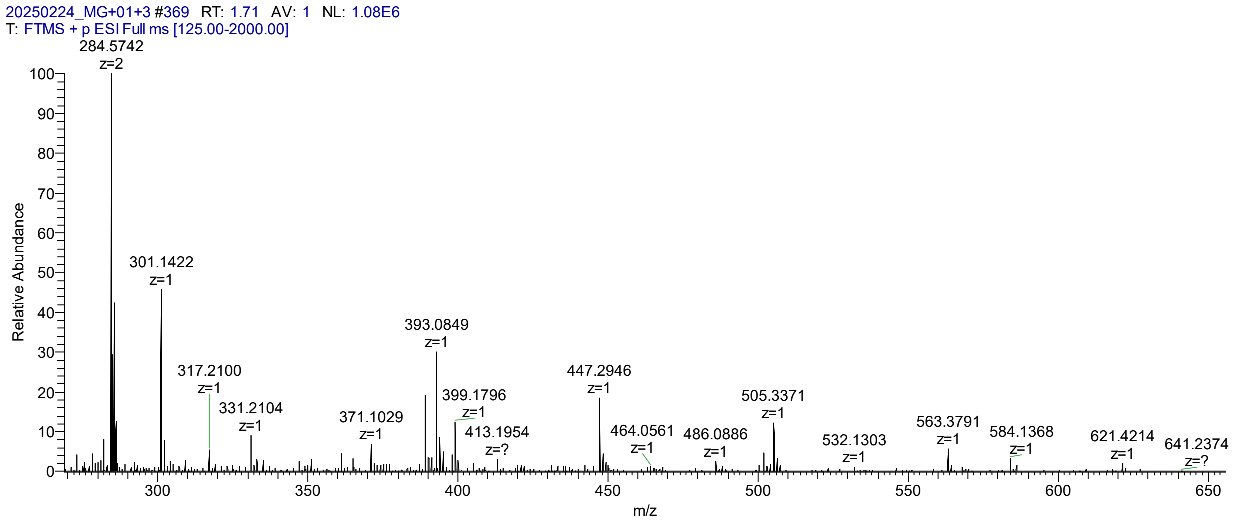

Figure 1. High resolution mass spectrum of Cu(II)-IL-Pyrasal: m/z calc. for $C_{28}H_{26}CuN_8O_2{}^{2+}$ 284.57, found at 284.5742.

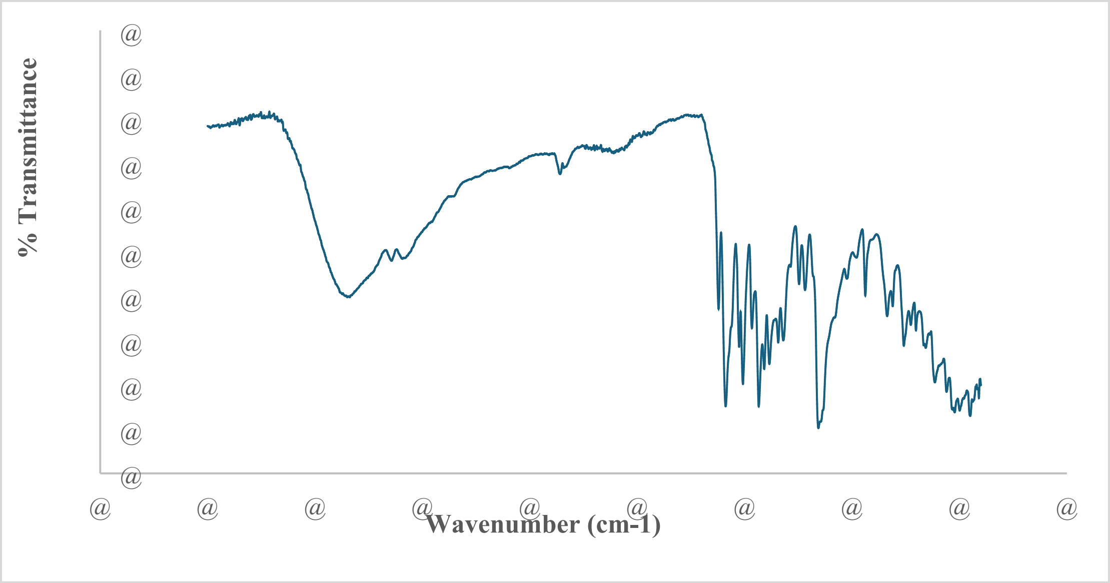

Figure 2. Infrared spectrum of Cu(II)-IL-Pyrasal complex

Figure 3. Ultraviolet-Visible spectroscopy of the catalyst with 20 µM solutions in CH_3CN

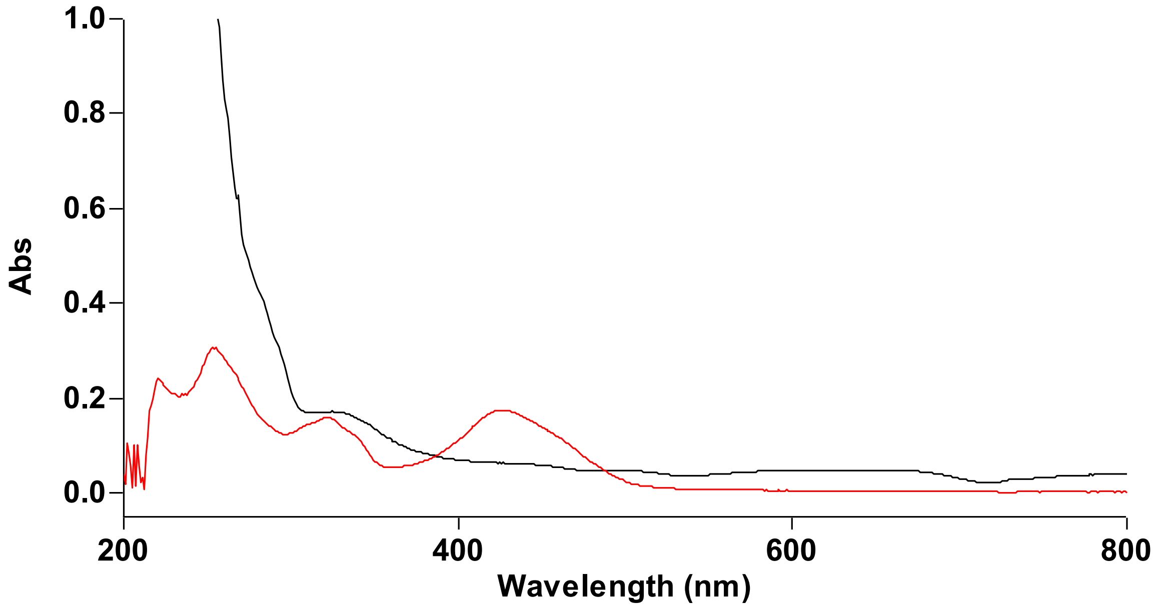

Sample Name: CH_3CN-Cu(II)-IL-Pyrasal

Peak Table	
Peak Style	Peaks
Peak Threshold	0.0100
Range	800.0nm to 200.0nm
Wavelength (nm)	Abs
426.0	0.175
323.0	0.159
255.0	0.306
220.0	0.241
208.0	0.103
206.0	0.101
202.0	0.106

Instrument Parameters

Instrument	Cary 60
Instrument Version	2.00
Start (nm)	800.0
Stop (nm)	200.0
X Mode	Nanometers
Y Mode	Abs
UV-Vis Scan Rate (nm/min)	600.00
UV-Vis Data Interval (nm)	1.00
UV-Vis Ave. Time (sec)	0.1000
Beam Mode	Dual Beam
Baseline Correction	On
Baseline Type	Baseline correction
Baseline Description	CH_3CN shown as black line
Cycle Mode	Off

Figure 4. Ultraviolet-Visible spectroscopy of the catalyst with 25 μM solutions in H_2O

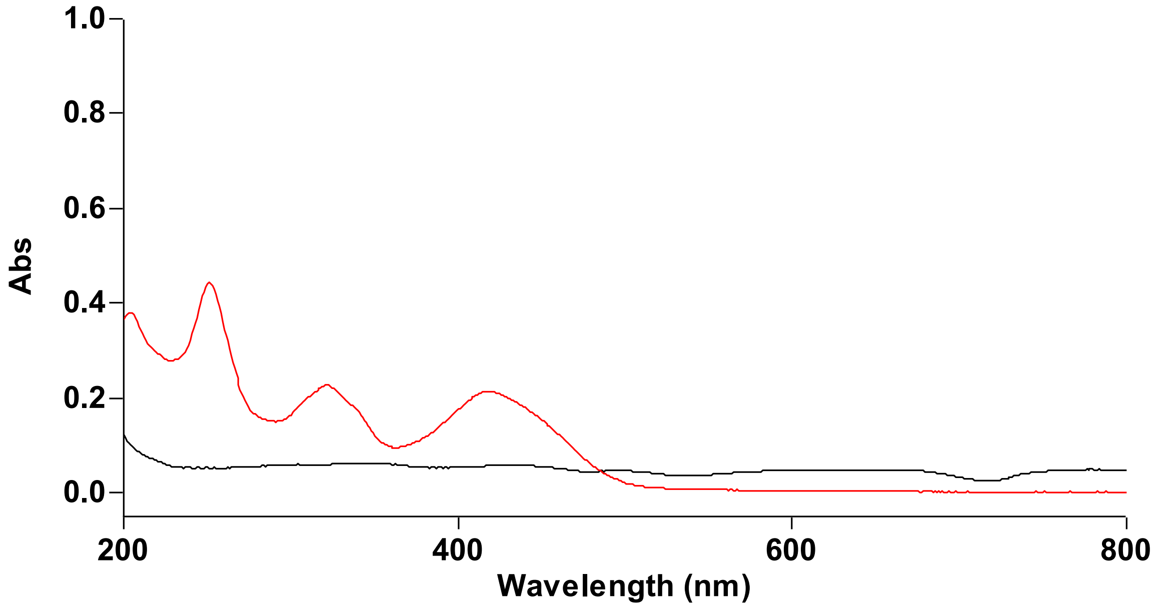

Sample Name: H_2O-Cu(II)-IL-Pyrasal

Peak Table	
Peak Style	Peaks
Peak Threshold	0.0100
Range	800.0nm to 200.0nm
Wavelength (nm)	Abs
419.0	0.213
322.0	0.227
251.0	0.443
204.0	0.379

Instrument Parameters

Instrument	Cary 60
Instrument Version	2.00
Start (nm)	800.0
Stop (nm)	200.0
X Mode	Nanometers
Y Mode	Abs
UV-Vis Scan Rate (nm/min)	600.00
UV-Vis Data Interval (nm)	1.00
UV-Vis Ave. Time (sec)	0.1000
Beam Mode	Dual Beam
Baseline Correction	On
Baseline Type	Baseline correction
Baseline Description	H_2O shown as black line
Cycle Mode	Off

Synthesis Method and Crystal growing steps:

To a round-bottom flask containing 10 mL of ethanol were added 8.75 mmol of compound 1 (2.25 g), 4.37 mmol of 2,3-diaminopyrazine (481 mg), and 4.81 mmol of copper(II) acetate (960 mg). The mixture was stirred for 4 hours at reflux temperature. The dark solid formed was filtered, washed with ice-cold ethanol, then dried in a vacuum oven to afford the metal complex with 72% yield (2.02 g) as a brown solid.

To obtain the PF_6^--substituted Cu-IL-pyrasal complex, the Cl^- counterion is first exchanged by dissolving the complex in water and mixing it with an aqueous solution of 2 equivalents KPF_6 (since 1 mol of catalyst contains 2 mol of IL). The resulting PF_6^- complex, which is poorly soluble in water, precipitates and can be isolated by filtration and drying steps.

For crystal growth, a small amount of the dried complex (1-3 mg) is dissolved in ~500 µL of acetonitrile inside a small shell vial, which is then placed inside a 20 ml screw-cap scintillation vial, filled with 1/3 hexanes or diethyl ether (both work as a co-solvents). After sealing the vial with cap and electrical tape to control evaporation. Slow diffusion of the nonpolar solvent into the acetonitrile solution promotes gradual crystallization of the complex as ($C_{28}H_{26}CuN_8O_2^+ \cdot 2\,PF_6^-$).

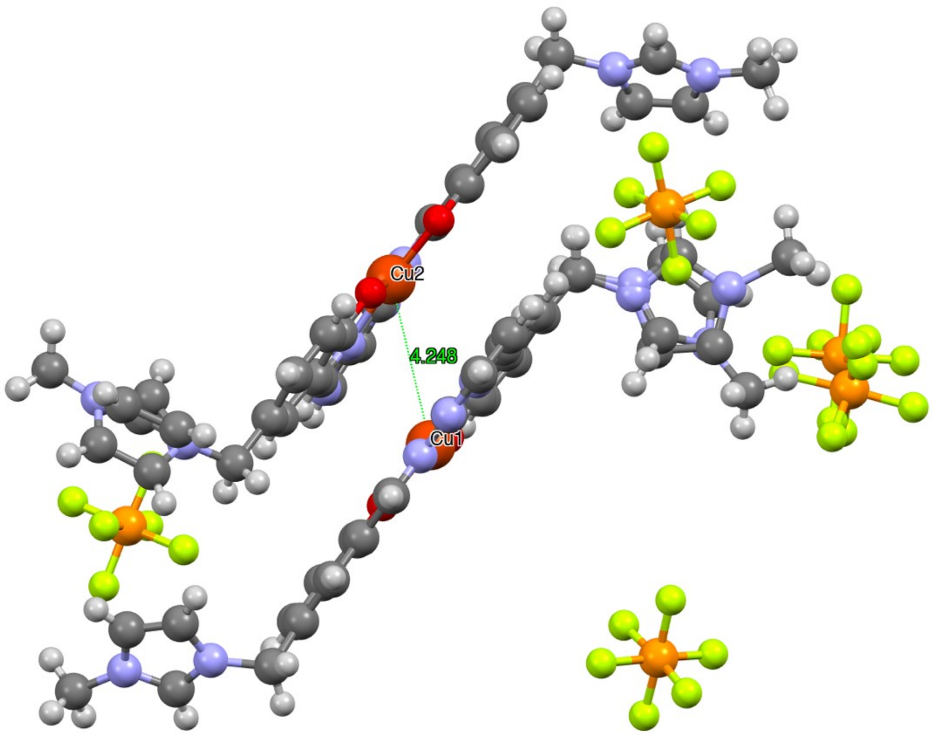

Figure 5. Twinning view of the coordination geometry in the Cu(II) complex

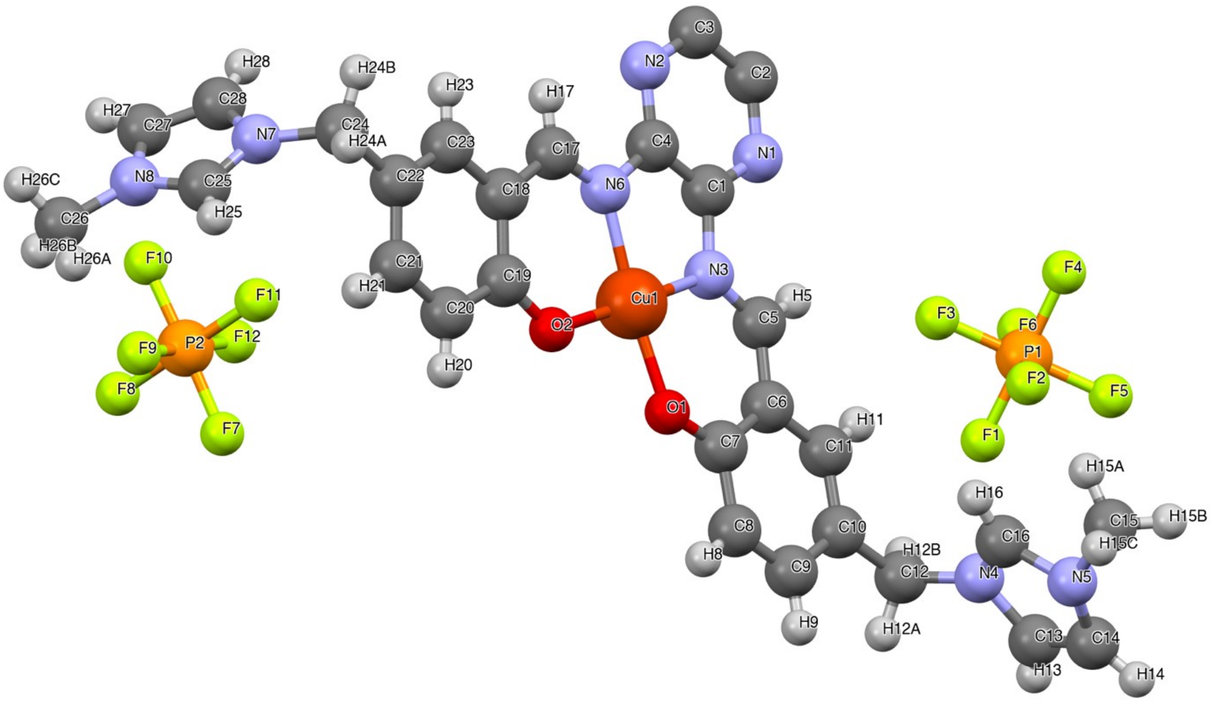

Figure 6. The X-Ray of the complex molecule $C_{28}H_{26}CuN_8O_2^+ \cdot 2PF_6^-$

Table 1. Crystal data and structure refinement for Cu(II)-IL-Pyrasal

Identification code	Agor25_06
Empirical formula	$C_{29}H_{27.16}CuF_{12}N_{8.5}O_2P_2$
Formula weight	880.24
Temperature/K	100.00(10)
Crystal system	monoclinic
Space group	$P2_1/c$
a/Å	8.8055(2)
b/Å	46.2813(10)
c/Å	18.7210(3)
α/°	90
β/°	95.683(2)
γ/°	90
Volume/Å^3	7591.9(3)
Z	8
ϱ_{calc}g/cm^3	1.540
μ/mm^{-1}	2.519
F(000)	3549.0
Crystal size/mm^3	0.175 × 0.086 × 0.044
Radiation	Cu Kα (λ = 1.54184)
2Θ range for data collection/°	5.114 to 136.496
Index ranges	-10 ≤ h ≤ 10, -47 ≤ k ≤ 55, -15 ≤ l ≤ 22
Reflections collected	59178
Independent reflections	13889 [R_{int} = 0.0644, R_{sigma} = 0.0450]
Data/restraints/parameters	13889/480/1070
Goodness-of-fit on F^2	1.062
Final R indexes [I>=2σ (I)]	R_1 = 0.0769, wR_2 = 0.2159
Final R indexes [all data]	R_1 = 0.0969, wR_2 = 0.2327
Largest diff. peak/hole / e Å^{-3}	0.73/-0.74

General Data Collection

Data were collected on a Rigaku XtaLAB Synergy-*i* Kappa diffractometer equipped with a PhotonJet-*i* X-ray source operated at 50 W (50kV, 1 mA) to generate Cu Kα radiation (λ = 1.54178 Å) and a HyPix-6000HE HPC detector. Crystals were transferred from the vial and placed on a glass slide in type NVH immersion oil by Cargille. A Zeiss Stemi 305 microscope was used to identify a suitable specimen for X-ray diffraction from a representative sample of the material. The crystal and a small amount of the oil were collected on a 100-micron MiTeGen CryoLoop and transferred to the instrument where it was placed under a cold nitrogen stream (Oxford 700 series) maintained at 100K throughout the duration of the experiment. The sample was optically centered with the aid of a video camera to insure that no translations were observed as the crystal was rotated through all positions.

Refinement Details (SQUEEZE)

After data collection, the unit cell was re-determined using a subset of the full data collection. Intensity data were corrected for Lorentz, polarization, and background effects using the *CrysAlis^Pro^*.[1] A numerical absorption correction was applied based on a Gaussian integration over a multifaceted crystal and followed by a semi-empirical correction for adsorption applied using the program *SCALE3 ABSPACK*.[2] The programs *SHELXT*[3] was used for the initial structure solution and *SHELXL*[4] was used for refinement of the structure. All these programs were utilized within the OLEX2 software.[5] Hydrogen atoms bound to the carbon and nitrogen atoms were in the difference Fourier map where possible and were geometrically constrained using the appropriate AFIX commands.

References:

(1) Rigaku, O. CrysAlisPro software system. *Version* **2018**, *1* (38.41), l.
(2) ABSPACK, S. v1. 0.7: an Oxford Diffraction program. Oxford Diffraction Ltd: Abingdon, UK: 2005.
(3) Sheldrick, G. M. Crystal structure refinement with SHELXL. *Crystal Structure Communications* **2015**, *71* (1), 3-8.
(4) Sheldrick, G. M. Crystal structure solution with ShelXT. *Acta Crystallogr. A* **2015**, *71*, 3-8.
(5) Dolomanov, O. V.; Bourhis, L. J.; Gildea, R. J.; Howard, J. A.; Puschmann, H. OLEX2: a complete structure solution, refinement and analysis program. *Applied Crystallography* **2009**, *42* (2), 339-341.

Bond Length and Angle

Number	Atom 1	Atom 2	Type	Polymeric	Cyclicity	Length	SybylType
**1**	Cu1	O1	Unknown	no	cyclic	1.894(3)	1
**2**	Cu1	O2	Unknown	no	cyclic	1.887(3)	1
**3**	Cu1	N3	Unknown	no	cyclic	1.943(3)	un
**4**	Cu1	N6	Unknown	no	cyclic	1.941(3)	un
**5**	O1	C7	Unknown	no	cyclic	1.302(5)	1
**6**	O2	C19	Unknown	no	cyclic	1.293(5)	1
**7**	N1	C1	Unknown	no	cyclic	1.328(5)	un
**8**	N1	C2	Unknown	no	cyclic	1.341(5)	un
**9**	N2	C3	Unknown	no	cyclic	1.336(5)	un
**10**	N2	C4	Unknown	no	cyclic	1.340(5)	un
**11**	N3	C1	Unknown	no	cyclic	1.415(5)	un
**12**	N3	C5	Unknown	no	cyclic	1.299(6)	un
**13**	N6	C4	Unknown	no	cyclic	1.407(5)	un
**14**	N6	C17	Unknown	no	cyclic	1.306(6)	un
**15**	N7	C24	Unknown	no	acyclic	1.469(7)	1
**16**	N7	C25	Unknown	no	cyclic	1.313(7)	un
**17**	N7	C28	Unknown	no	cyclic	1.356(8)	un
**18**	N8	C25	Unknown	no	cyclic	1.328(8)	un
**19**	N8	C26	Unknown	no	acyclic	1.45(1)	1
**20**	N8	C27	Unknown	no	cyclic	1.35(1)	un
**21**	C1	C4	Unknown	no	cyclic	1.390(6)	un
**22**	C2	H2	Unknown	no	acyclic	0.951	1
**23**	C2	C3	Unknown	no	cyclic	1.389(7)	un
**24**	C3	H3	Unknown	no	acyclic	0.951	1

**25**	C5	H5	Unknown	no	acyclic	0.951	1
**26**	C5	C6	Unknown	no	cyclic	1.423(6)	un
**27**	C6	C7	Unknown	no	cyclic	1.435(5)	un
**28**	C6	C11	Unknown	no	cyclic	1.416(6)	un
**29**	C7	C8	Unknown	no	cyclic	1.416(6)	un
**30**	C8	H8	Unknown	no	acyclic	0.949	1
**31**	C8	C9	Unknown	no	cyclic	1.362(8)	un
**32**	C9	H9	Unknown	no	acyclic	0.95	1
**33**	C9	C10	Unknown	no	cyclic	1.418(6)	un
**34**	C10	C11	Unknown	no	cyclic	1.369(6)	un
**35**	C10	C12	Unknown	no	acyclic	1.484(7)	1
**36**	C11	H11	Unknown	no	acyclic	0.95	1
**37**	C12	H12C	Unknown	no	acyclic	0.99	1
**38**	C12	H12D	Unknown	no	acyclic	0.99	1
**39**	C12	N4A	Unknown	no	acyclic	1.428(9)	1
**40**	C14A	C13A	Unknown	no	cyclic	1.42(1)	1
**41**	C14A	N5A	Unknown	no	cyclic	1.42(1)	1
**42**	C13A	H13A	Unknown	no	acyclic	0.949	1
**43**	C13A	N4A	Unknown	no	cyclic	1.42(1)	un
**44**	N4A	C16A	Unknown	no	cyclic	1.42(1)	un
**45**	C16A	H16A	Unknown	no	acyclic	0.95	1
**46**	C16A	N5A	Unknown	no	cyclic	1.42(1)	un
**47**	N5A	C15A	Unknown	no	acyclic	1.64(2)	1
**48**	C15A	H15D	Unknown	no	acyclic	0.98	1
**49**	C15A	H15E	Unknown	no	acyclic	0.98	1
**50**	C15A	H15F	Unknown	no	acyclic	0.98	1
**51**	C17	H17	Unknown	no	acyclic	0.95	1
**52**	C17	C18	Unknown	no	cyclic	1.424(5)	un
**53**	C18	C19	Unknown	no	cyclic	1.436(6)	un
**54**	C18	C23	Unknown	no	cyclic	1.431(6	un

						)	
**55**	C19	C20	Unknown	no	cyclic	1.424(6<br>)	un
**56**	C20	H20	Unknown	no	acyclic	0.95	1
**57**	C20	C21	Unknown	no	cyclic	1.354(7<br>)	un
**58**	C21	H21	Unknown	no	acyclic	0.95	1
**59**	C21	C22	Unknown	no	cyclic	1.416(8<br>)	un
**60**	C22	C23	Unknown	no	cyclic	1.367(6<br>)	un
**61**	C22	C24	Unknown	no	acyclic	1.505(7<br>)	1
**62**	C23	H23	Unknown	no	acyclic	0.95	1
**63**	C24	H24A	Unknown	no	acyclic	0.99	1
**64**	C24	H24B	Unknown	no	acyclic	0.99	1
**65**	C25	H25	Unknown	no	acyclic	0.95	1
**66**	C26	H26A	Unknown	no	acyclic	0.98	1
**67**	C26	H26B	Unknown	no	acyclic	0.98	1
**68**	C26	H26C	Unknown	no	acyclic	0.98	1
**69**	C27	H27	Unknown	no	acyclic	0.95	1
**70**	C27	C28	Unknown	no	cyclic	1.32(1)	un
**71**	C28	H28	Unknown	no	acyclic	0.951	1
**72**	Cu2	O3	Unknown	no	cyclic	1.902(3<br>)	1
**73**	Cu2	O4	Unknown	no	cyclic	1.895(3<br>)	1
**74**	Cu2	N11	Unknown	no	cyclic	1.940(3<br>)	un
**75**	Cu2	N12	Unknown	no	cyclic	1.940(3<br>)	un
**76**	O3	C35	Unknown	no	cyclic	1.293(5<br>)	1
**77**	O4	C46	Unknown	no	cyclic	1.288(5<br>)	1
**78**	N9	C29	Unknown	no	cyclic	1.324(5<br>)	un
**79**	N9	C30	Unknown	no	cyclic	1.333(6<br>)	un
**80**	N10	C31	Unknown	no	cyclic	1.339(5<br>)	un
**81**	N10	C32	Unknown	no	cyclic	1.326(6<br>)	un
**82**	N11	C29	Unknown	no	cyclic	1.404(5<br>)	un

**83**	N11	C33	Unknown	no	cyclic	1.306(6)	un
**84**	N12	C32	Unknown	no	cyclic	1.412(5)	un
**85**	N12	C45	Unknown	no	cyclic	1.303(6)	un
**86**	N15	C52	Unknown	no	acyclic	1.460(7)	1
**87**	N15	C53	Unknown	no	cyclic	1.358(9)	un
**88**	N15	C56	Unknown	no	cyclic	1.308(9)	un
**89**	N16	C54	Unknown	no	cyclic	1.36(1)	un
**90**	N16	C55	Unknown	no	acyclic	1.47(1)	1
**91**	N16	C56	Unknown	no	cyclic	1.27(1)	un
**92**	C29	C32	Unknown	no	cyclic	1.398(6)	un
**93**	C30	H30	Unknown	no	acyclic	0.949	1
**94**	C30	C31	Unknown	no	cyclic	1.391(7)	un
**95**	C31	H31	Unknown	no	acyclic	0.949	1
**96**	C33	H33	Unknown	no	acyclic	0.95	1
**97**	C33	C34	Unknown	no	cyclic	1.416(6)	un
**98**	C34	C35	Unknown	no	cyclic	1.435(5)	un
**99**	C34	C39	Unknown	no	cyclic	1.417(6)	un
**100**	C35	C36	Unknown	no	cyclic	1.423(7)	un
**101**	C36	H36	Unknown	no	acyclic	0.95	1
**102**	C36	C37	Unknown	no	cyclic	1.364(7)	un
**103**	C37	H37	Unknown	no	acyclic	0.951	1
**104**	C37	C38	Unknown	no	cyclic	1.407(8)	un
**105**	C38	C39	Unknown	no	cyclic	1.370(8)	un
**106**	C38	C40	Unknown	no	acyclic	1.501(7)	1
**107**	C39	H39	Unknown	no	acyclic	0.95	1
**108**	C40	H40A	Unknown	no	acyclic	0.99	1
**109**	C40	H40B	Unknown	no	acyclic	0.99	1
**110**	C40	N14	Unknown	no	acyclic	1.420(7)	1
**111**	C41A	H41A	Unknown	no	acyclic	0.95	1

**112**	C41A	N14	Unknown	no	cyclic	1.420(6<br>)	un
**113**	C41A	N13	Unknown	no	cyclic	1.420(7<br>)	un
**114**	N14	C44A	Unknown	no	cyclic	1.419(7<br>)	un
**115**	C44A	H44A	Unknown	no	acyclic	0.95	1
**116**	C44A	C43A	Unknown	no	cyclic	1.420(7<br>)	un
**117**	C43A	H43A	Unknown	no	acyclic	0.95	1
**118**	C43A	N13	Unknown	no	cyclic	1.420(8<br>)	un
**119**	N13	C42	Unknown	no	acyclic	1.37(1)	1
**120**	C42	H42A	Unknown	no	acyclic	0.98	1
**121**	C42	H42B	Unknown	no	acyclic	0.98	1
**122**	C42	H42C	Unknown	no	acyclic	0.98	1
**123**	C45	H45	Unknown	no	acyclic	0.95	1
**124**	C45	C51	Unknown	no	cyclic	1.423(5<br>)	un
**125**	C46	C47	Unknown	no	cyclic	1.422(6<br>)	un
**126**	C46	C51	Unknown	no	cyclic	1.437(6<br>)	un
**127**	C47	H47	Unknown	no	acyclic	0.951	1
**128**	C47	C48	Unknown	no	cyclic	1.366(6<br>)	un
**129**	C48	H48	Unknown	no	acyclic	0.949	1
**130**	C48	C49	Unknown	no	cyclic	1.413(7<br>)	un
**131**	C49	C50	Unknown	no	cyclic	1.365(7<br>)	un
**132**	C49	C52	Unknown	no	acyclic	1.509(7<br>)	1
**133**	C50	H50	Unknown	no	acyclic	0.949	1
**134**	C50	C51	Unknown	no	cyclic	1.418(6<br>)	un
**135**	C52	H52A	Unknown	no	acyclic	0.99	1
**136**	C52	H52B	Unknown	no	acyclic	0.99	1
**137**	C53	H53	Unknown	no	acyclic	0.95	1
**138**	C53	C54	Unknown	no	cyclic	1.33(1)	un
**139**	C54	H54	Unknown	no	acyclic	0.95	1
**140**	C55	H55A	Unknown	no	acyclic	0.98	1

**141**	C55	H55B	Unknown	no	acyclic	0.98	1
**142**	C55	H55C	Unknown	no	acyclic	0.98	1
**143**	C56	H56	Unknown	no	acyclic	0.951	1
**144**	P1	F1	Unknown	no	acyclic	1.583(4)	1
**145**	P1	F2	Unknown	no	acyclic	1.604(4)	1
**146**	P1	F3	Unknown	no	acyclic	1.602(4)	1
**147**	P1	F4	Unknown	no	acyclic	1.596(5)	1
**148**	P1	F5	Unknown	no	acyclic	1.591(5)	1
**149**	P1	F6	Unknown	no	acyclic	1.597(4)	1
**150**	P2	F7	Unknown	no	acyclic	1.562(9)	1
**151**	P2	F8	Unknown	no	acyclic	1.524(8)	1
**152**	P2	F9	Unknown	no	acyclic	1.539(9)	1
**153**	P2	F10	Unknown	no	acyclic	1.573(6)	1
**154**	P2	F11	Unknown	no	acyclic	1.585(8)	1
**155**	P2	F12	Unknown	no	acyclic	1.542(6)	1
**156**	P3	F13	Unknown	no	acyclic	1.565(6)	1
**157**	P3	F14	Unknown	no	acyclic	1.601(4)	1
**158**	P3	F15	Unknown	no	acyclic	1.527(7)	1
**159**	P3	F16	Unknown	no	acyclic	1.571(6)	1
**160**	P3	F23	Unknown	no	acyclic	1.582(4)	1
**161**	P3	F24	Unknown	no	acyclic	1.593(5)	1
**162**	P4	F17	Unknown	no	acyclic	1.58(2)	1
**163**	P4	F18	Unknown	no	acyclic	1.61(1)	1
**164**	P4	F19	Unknown	no	acyclic	1.58(2)	1
**165**	P4	F20	Unknown	no	acyclic	1.60(2)	1
**166**	P4	F21	Unknown	no	acyclic	1.60(2)	1
**167**	P4	F22	Unknown	no	acyclic	1.60(1)	1
**168**	N17	C57	Unknown	no	acyclic	1.146(8)	un

**169**	C57	C58	Unknown	no	acyclic	1.461(9)	1
**170**	C58	H58A	Unknown	no	acyclic	0.98	1
**171**	C58	H58B	Unknown	no	acyclic	0.98	1
**172**	C58	H58C	Unknown	no	acyclic	0.98	1

Number	Atom1	Atom2	Atom3	Angle
1	O1	Cu1	O2	88.2(1)
2	O1	Cu1	N3	93.6(1)
3	O1	Cu1	N6	174.1(1)
4	O2	Cu1	N3	176.7(1)
5	O2	Cu1	N6	93.6(1)
6	N3	Cu1	N6	84.9(1)
7	Cu1	O1	C7	127.1(3)
8	Cu1	O2	C19	127.6(3)
9	C1	N1	C2	115.1(4)
10	C3	N2	C4	115.4(4)
11	Cu1	N3	C1	111.6(3)
12	Cu1	N3	C5	126.7(3)
13	C1	N3	C5	121.7(4)
14	Cu1	N6	C4	111.7(3)
15	Cu1	N6	C17	126.7(3)
16	C4	N6	C17	121.6(4)
17	C24	N7	C25	125.0(5)
18	C24	N7	C28	126.4(5)
19	C25	N7	C28	108.6(5)
20	C25	N8	C26	125.2(7)
21	C25	N8	C27	108.6(6)
22	C26	N8	C27	126.2(7)
23	N1	C1	N3	121.4(4)
24	N1	C1	C4	122.9(4)
25	N3	C1	C4	115.7(4)
26	N1	C2	H2	118.8
27	N1	C2	C3	122.5(4)
28	H2	C2	C3	118.7
29	N2	C3	C2	122.2(4)
30	N2	C3	H3	118.9
31	C2	C3	H3	118.9
32	N2	C4	N6	121.9(4)

33	N2	C4	C1	122.0(4)
34	N6	C4	C1	116.1(4)
35	N3	C5	H5	117.8
36	N3	C5	C6	124.4(4)
37	H5	C5	C6	117.8
38	C5	C6	C7	123.1(4)
39	C5	C6	C11	117.6(4)
40	C7	C6	C11	119.2(4)
41	O1	C7	C6	125.0(4)
42	O1	C7	C8	117.9(4)
43	C6	C7	C8	117.2(4)
44	C7	C8	H8	119
45	C7	C8	C9	122.1(4)
46	H8	C8	C9	118.9
47	C8	C9	H9	119.6
48	C8	C9	C10	120.9(4)
49	H9	C9	C10	119.6
50	C9	C10	C11	118.6(4)
51	C9	C10	C12	120.2(4)
52	C11	C10	C12	121.2(4)
53	C6	C11	C10	122.0(4)
54	C6	C11	H11	119
55	C10	C11	H11	119
56	C10	C12	H12C	109.8
57	C10	C12	H12D	109.7
58	C10	C12	H12A	107.7
59	C10	C12	H12B	107.7
60	C10	C12	N4A	109.5(5)
61	C10	C12	N4	118.6(8)
62	H12C	C12	H12D	108.2
63	H12C	C12	H12A	2.3
64	H12C	C12	H12B	105.3
65	H12C	C12	N4A	109.7
66	H12C	C12	N4	107.1
67	H12D	C12	H12A	110.1
68	H12D	C12	H12B	4.92
69	H12D	C12	N4A	109.8
70	H12D	C12	N4	102.9
71	H12A	C12	H12B	107.1
72	H12A	C12	N4A	109.9

73	H12A	C12	N4	107.7
74	H12B	C12	N4A	114.7
75	H12B	C12	N4	107.7
76	N4A	C12	N4	9.4(7)
77	C13A	C14A	N5A	108.0(8)
78	C14A	C13A	H13A	125.9
79	C14A	C13A	N4A	107.9(8)
80	H13A	C13A	N4A	126.1
81	C12	N4A	C13A	131.3(7)
82	C12	N4A	C16A	119.2(7)
83	C13A	N4A	C16A	108.0(7)
84	N4A	C16A	H16A	126
85	N4A	C16A	N5A	108.0(7)
86	H16A	C16A	N5A	126
87	C14A	N5A	C16A	108.0(8)
88	C14A	N5A	C15A	131.2(9)
89	C16A	N5A	C15A	120.8(9)
90	H15A	C15	H15B	109
91	H15A	C15	H15C	109
92	H15A	C15	N5	109
93	H15B	C15	H15C	110
94	H15B	C15	N5	109
95	H15C	C15	N5	110
96	N5A	C15A	H15D	109
97	N5A	C15A	H15E	110
98	N5A	C15A	H15F	109
99	H15D	C15A	H15E	110
100	H15D	C15A	H15F	109
101	H15E	C15A	H15F	110
102	H16	C16	N4	126
103	H16	C16	N5	126
104	N4	C16	N5	108(1)
105	C12	N4	C16	123(1)
106	C12	N4	C13	123(1)
107	C16	N4	C13	108(1)
108	N4	C13	H13	126
109	N4	C13	C14	108(1)
110	H13	C13	C14	126
111	C13	C14	H14	126
112	C13	C14	N5	108(2)

113	H14	C14	N5	126
114	C15	N5	C16	107(2)
115	C15	N5	C14	142(2)
116	C16	N5	C14	108(2)
117	N6	C17	H17	118.1
118	N6	C17	C18	123.8(4)
119	H17	C17	C18	118.1
120	C17	C18	C19	123.4(4)
121	C17	C18	C23	117.4(4)
122	C19	C18	C23	119.1(4)
123	O2	C19	C18	124.8(4)
124	O2	C19	C20	118.5(4)
125	C18	C19	C20	116.7(4)
126	C19	C20	H20	118.7
127	C19	C20	C21	122.5(4)
128	H20	C20	C21	118.7
129	C20	C21	H21	119.4
130	C20	C21	C22	121.1(5)
131	H21	C21	C22	119.5
132	C21	C22	C23	118.7(4)
133	C21	C22	C24	120.0(4)
134	C23	C22	C24	121.3(4)
135	C18	C23	C22	121.8(4)
136	C18	C23	H23	119.1
137	C22	C23	H23	119.1
138	N7	C24	C22	112.0(4)
139	N7	C24	H24A	109.2
140	N7	C24	H24B	109.2
141	C22	C24	H24A	109.2
142	C22	C24	H24B	109.2
143	H24A	C24	H24B	107.9
144	N7	C25	N8	107.7(5)
145	N7	C25	H25	126.1
146	N8	C25	H25	126.1
147	N8	C26	H26A	109.5
148	N8	C26	H26B	109.4
149	N8	C26	H26C	109.4
150	H26A	C26	H26B	110
151	H26A	C26	H26C	109
152	H26B	C26	H26C	109

153	N8	C27	H27	126.3
154	N8	C27	C28	107.6(7)
155	H27	C27	C28	126.2
156	N7	C28	C27	107.5(7)
157	N7	C28	H28	126.2
158	C27	C28	H28	126.2
159	O3	Cu2	O4	89.0(1)
160	O3	Cu2	N11	93.6(1)
161	O3	Cu2	N12	174.4(1)
162	O4	Cu2	N11	174.8(1)
163	O4	Cu2	N12	93.3(1)
164	N11	Cu2	N12	84.5(1)
165	Cu2	O3	C35	127.2(3)
166	Cu2	O4	C46	127.7(3)
167	C29	N9	C30	115.8(4)
168	C31	N10	C32	115.1(4)
169	Cu2	N11	C29	112.4(3)
170	Cu2	N11	C33	126.0(3)
171	C29	N11	C33	121.5(4)
172	Cu2	N12	C32	112.1(3)
173	Cu2	N12	C45	126.8(3)
174	C32	N12	C45	121.0(4)
175	C52	N15	C53	126.2(5)
176	C52	N15	C56	126.9(5)
177	C53	N15	C56	106.9(6)
178	C54	N16	C55	124.2(8)
179	C54	N16	C56	107.8(7)
180	C55	N16	C56	127.9(8)
181	N9	C29	N11	122.3(4)
182	N9	C29	C32	122.2(4)
183	N11	C29	C32	115.4(4)
184	N9	C30	H30	119.1
185	N9	C30	C31	121.9(4)
186	H30	C30	C31	119
187	N10	C31	C30	122.6(4)
188	N10	C31	H31	118.7
189	C30	C31	H31	118.7
190	N10	C32	N12	122.1(4)
191	N10	C32	C29	122.4(4)
192	N12	C32	C29	115.5(4)

193	N11	C33	H33	117.5
194	N11	C33	C34	125.0(4)
195	H33	C33	C34	117.5
196	C33	C34	C35	123.0(4)
197	C33	C34	C39	117.3(4)
198	C35	C34	C39	119.7(4)
199	O3	C35	C34	125.1(4)
200	O3	C35	C36	118.0(4)
201	C34	C35	C36	116.9(4)
202	C35	C36	H36	119.4
203	C35	C36	C37	121.2(5)
204	H36	C36	C37	119.4
205	C36	C37	H37	118.8
206	C36	C37	C38	122.3(5)
207	H37	C37	C38	118.9
208	C37	C38	C39	118.1(5)
209	C37	C38	C40	121.4(5)
210	C39	C38	C40	120.5(5)
211	C34	C39	C38	121.8(4)
212	C34	C39	H39	119
213	C38	C39	H39	119.1
214	C38	C40	H40A	109
215	C38	C40	H40B	109
216	C38	C40	N14	113.1(5)
217	H40A	C40	H40B	107.8
218	H40A	C40	N14	109
219	H40B	C40	N14	108.9
220	H41	C41	N14	123
221	H41	C41	N13	123
222	N14	C41	N13	113.9(8)
223	H41A	C41A	N14	126
224	H41A	C41A	N13	126
225	N14	C41A	N13	108.0(4)
226	C40	N14	C41	126.4(6)
227	C40	N14	C41A	125.9(5)
228	C40	N14	C44A	124.5(5)
229	C40	N14	C44	124.1(7)
230	C41	N14	C41A	21.5(5)
231	C41	N14	C44A	98.6(6)
232	C41	N14	C44	106.9(8)

233	C41A	N14	C44A	108.0(4)
234	C41A	N14	C44	97.2(6)
235	C44A	N14	C44	51.6(6)
236	N14	C44A	H44A	126
237	N14	C44A	C43A	108.0(5)
238	H44A	C44A	C43A	126
239	C44A	C43A	H43A	126
240	C44A	C43A	N13	108.0(6)
241	H43A	C43A	N13	126
242	C41	N13	C41A	21.5(5)
243	C41	N13	C43A	98.6(6)
244	C41	N13	C42	130.1(7)
245	C41	N13	C43	102.4(8)
246	C41A	N13	C43A	108.0(5)
247	C41A	N13	C42	128.8(6)
248	C41A	N13	C43	94.1(6)
249	C43A	N13	C42	122.3(6)
250	C43A	N13	C43	48.3(6)
251	C42	N13	C43	125.5(8)
252	N13	C42	H42A	109.5
253	N13	C42	H42B	109.5
254	N13	C42	H42C	109.5
255	H42A	C42	H42B	110
256	H42A	C42	H42C	109
257	H42B	C42	H42C	109
258	N13	C43	H43	125
259	N13	C43	C44	110(1)
260	H43	C43	C44	125
261	N14	C44	C43	104(1)
262	N14	C44	H44	128
263	C43	C44	H44	128
264	N12	C45	H45	118
265	N12	C45	C51	123.9(4)
266	H45	C45	C51	118.1
267	O4	C46	C47	118.4(4)
268	O4	C46	C51	124.7(4)
269	C47	C46	C51	116.8(4)
270	C46	C47	H47	119
271	C46	C47	C48	122.0(4)
272	H47	C47	C48	119

273	C47	C48	H48	119.4
274	C47	C48	C49	121.2(4)
275	H48	C48	C49	119.4
276	C48	C49	C50	118.4(4)
277	C48	C49	C52	120.6(4)
278	C50	C49	C52	121.0(4)
279	C49	C50	H50	118.8
280	C49	C50	C51	122.4(4)
281	H50	C50	C51	118.8
282	C45	C51	C46	123.3(4)
283	C45	C51	C50	117.5(4)
284	C46	C51	C50	119.2(4)
285	N15	C52	C49	112.1(4)
286	N15	C52	H52A	109.2
287	N15	C52	H52B	109.2
288	C49	C52	H52A	109.2
289	C49	C52	H52B	109.2
290	H52A	C52	H52B	107.9
291	N15	C53	H53	126.7
292	N15	C53	C54	106.7(6)
293	H53	C53	C54	126.6
294	N16	C54	C53	107.4(7)
295	N16	C54	H54	126.3
296	C53	C54	H54	126.3
297	N16	C55	H55A	109
298	N16	C55	H55B	110
299	N16	C55	H55C	110
300	H55A	C55	H55B	109
301	H55A	C55	H55C	109
302	H55B	C55	H55C	110
303	N15	C56	N16	111.2(7)
304	N15	C56	H56	124.4
305	N16	C56	H56	124.4
306	F1	P1	F2	90.0(2)
307	F1	P1	F3	90.0(2)
308	F1	P1	F4	178.7(2)
309	F1	P1	F5	90.1(2)
310	F1	P1	F6	90.1(2)
311	F2	P1	F3	88.5(2)
312	F2	P1	F4	89.7(2)

313	F2	P1	F5	90.2(2)
314	F2	P1	F6	178.8(2)
315	F3	P1	F4	88.7(2)
316	F3	P1	F5	178.7(3)
317	F3	P1	F6	90.4(2)
318	F4	P1	F5	91.1(2)
319	F4	P1	F6	90.2(2)
320	F5	P1	F6	91.0(3)
321	F7	P2	F8	84.6(4)
322	F7	P2	F9	90.9(5)
323	F7	P2	F10	178.4(4)
324	F7	P2	F11	95.6(4)
325	F7	P2	F12	89.3(4)
326	F8	P2	F9	90.5(5)
327	F8	P2	F10	93.9(4)
328	F8	P2	F11	178.9(4)
329	F8	P2	F12	91.8(4)
330	F9	P2	F10	88.7(4)
331	F9	P2	F11	90.6(5)
332	F9	P2	F12	177.7(4)
333	F10	P2	F11	85.9(4)
334	F10	P2	F12	91.1(3)
335	F11	P2	F12	87.1(4)
336	F13	P3	F14	89.1(3)
337	F13	P3	F15	92.7(4)
338	F13	P3	F16	173.6(3)
339	F13	P3	F23	89.9(3)
340	F13	P3	F24	87.3(3)
341	F14	P3	F15	88.7(4)
342	F14	P3	F16	88.8(3)
343	F14	P3	F23	178.4(3)
344	F14	P3	F24	89.6(3)
345	F15	P3	F16	93.2(4)
346	F15	P3	F23	92.6(4)
347	F15	P3	F24	178.3(4)
348	F16	P3	F23	92.1(3)
349	F16	P3	F24	86.7(3)
350	F23	P3	F24	89.1(3)
351	F17	P4	F18	92(1)
352	F17	P4	F19	90.9(9)

353	F17	P4	F20	178(1)
354	F17	P4	F21	88(1)
355	F17	P4	F22	90.1(9)
356	F18	P4	F19	90(1)
357	F18	P4	F20	90(1)
358	F18	P4	F21	88(1)
359	F18	P4	F22	178(1)
360	F19	P4	F20	90.5(9)
361	F19	P4	F21	177(1)
362	F19	P4	F22	90.8(9)
363	F20	P4	F21	91(1)
364	F20	P4	F22	88.0(9)
365	F21	P4	F22	92(1)
366	F17A	P4A	F18A	91(1)
367	F17A	P4A	F19A	90(1)
368	F17A	P4A	F20A	177(1)
369	F17A	P4A	F21A	89(1)
370	F17A	P4A	F22A	89(1)
371	F18A	P4A	F19A	89(1)
372	F18A	P4A	F20A	90.3(8)
373	F18A	P4A	F21A	179.2(9)
374	F18A	P4A	F22A	87.9(8)
375	F19A	P4A	F20A	92.4(9)
376	F19A	P4A	F21A	91.7(9)
377	F19A	P4A	F22A	177(1)
378	F20A	P4A	F21A	89.1(7)
379	F20A	P4A	F22A	88.4(7)
380	F21A	P4A	F22A	91.6(7)
381	N17	C57	C58	177.9(7)
382	C57	C58	H58A	109.5
383	C57	C58	H58B	109.5
384	C57	C58	H58C	109.5
385	H58A	C58	H58B	109.5
386	H58A	C58	H58C	109.5
387	H58B	C58	H58C	109.5

^1H NMR (400 MHz, Chloroform-*d*) δ 7.99 – 7.81 (m, 2H), 7.54 – 7.47 (m, 1H), 7.44 – 7.29 (m, 2H), 2.55 (s, 3H).

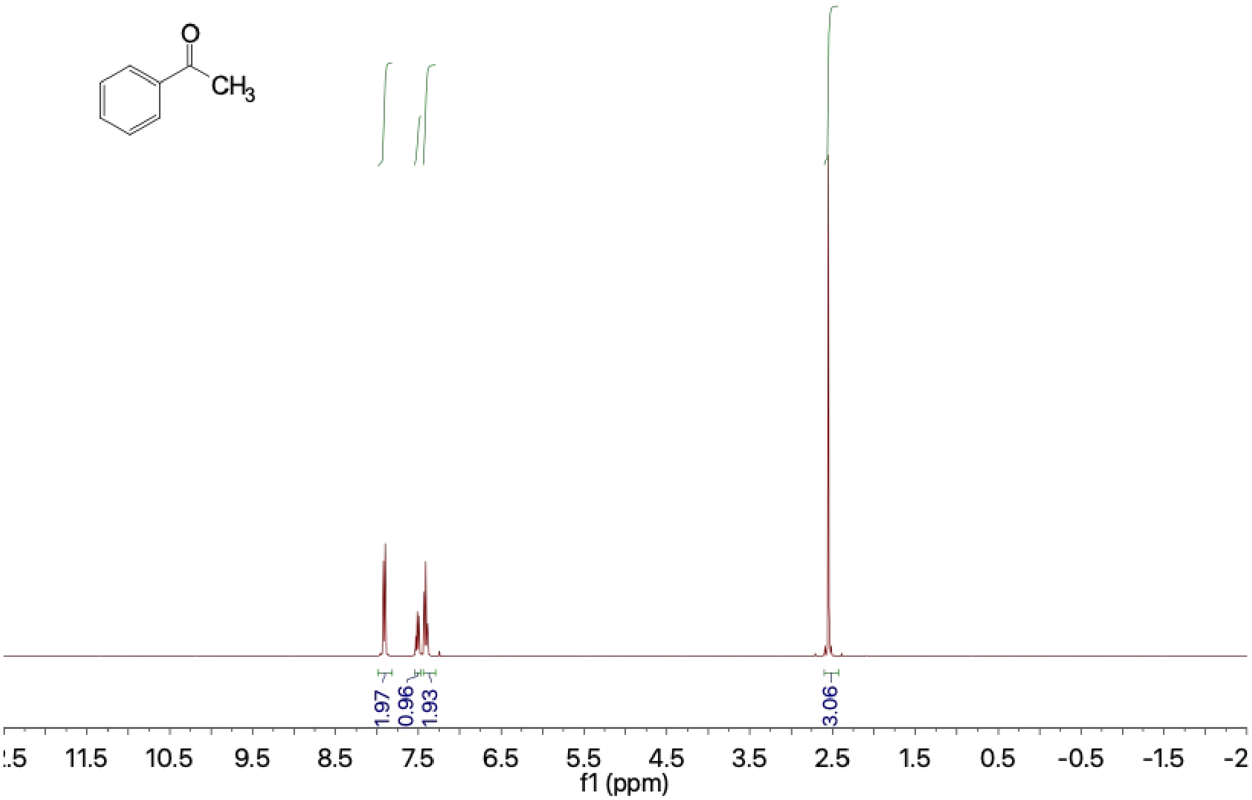

^{1}H NMR (400 MHz, Chloroform-*d*) δ 7.87 – 7.73 (m, 4H), 7.66 – 7.52 (m, 2H), 7.56 – 7.40 (m, 4H).

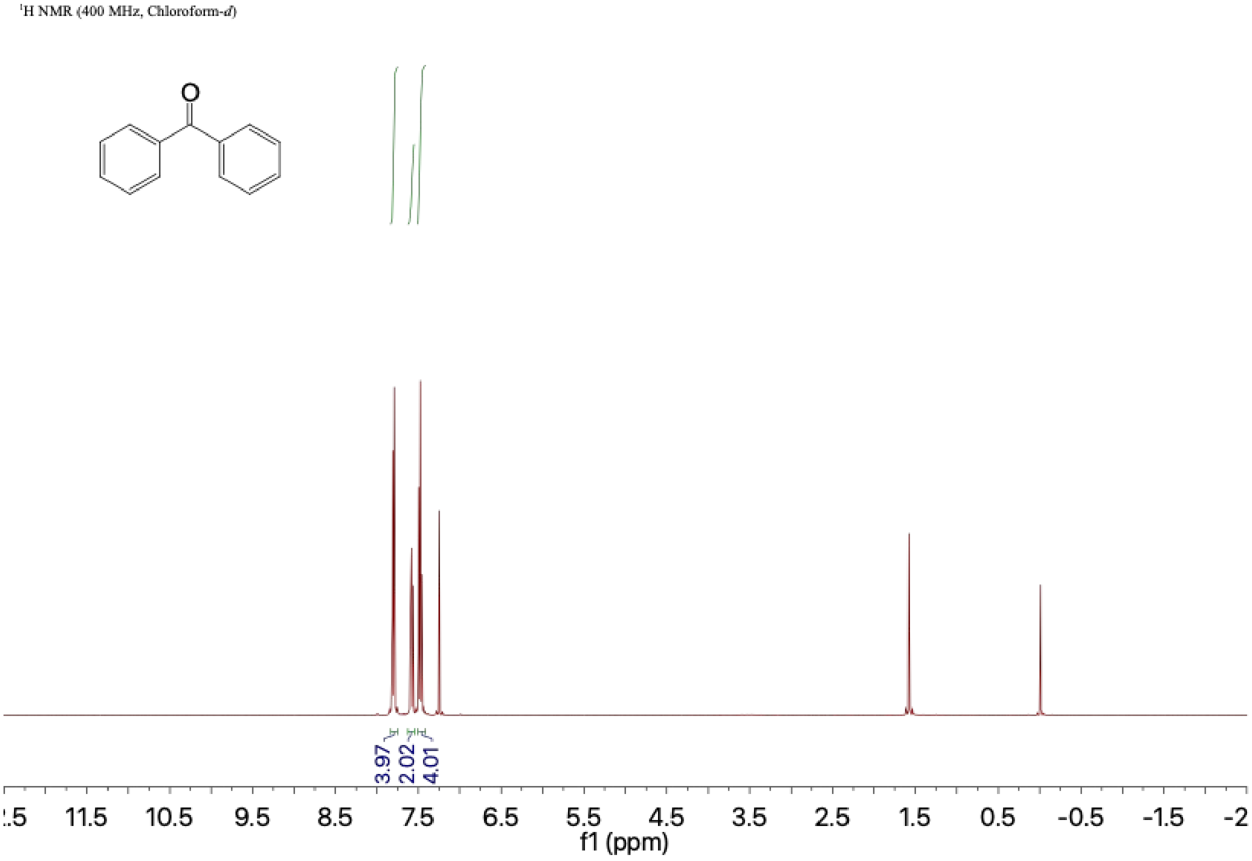

^{1}H NMR (400 MHz, Chloroform-*d*) δ 8.33 (ddd, *J* = 8.0, 1.8, 0.9 Hz, 2H), 7.80 – 7.64 (m, 2H), 7.56 – 7.42 (m, 2H), 7.37 (ddt, *J* = 8.1, 7.1, 1.0 Hz, 2H).

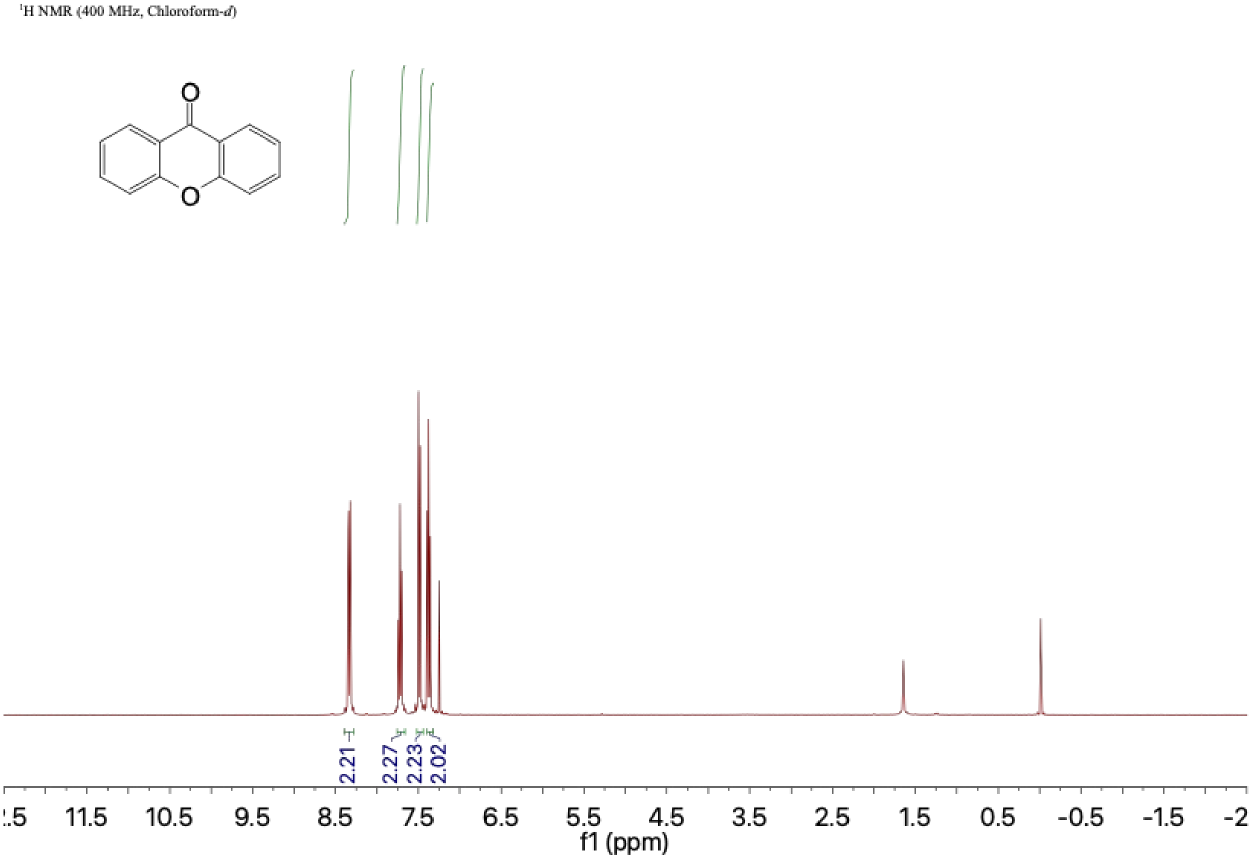

^1H NMR (400 MHz, Chloroform-*d*) δ 10.01 (s, 1H), 7.95 – 7.80 (m, 2H), 7.63 (ddt, *J* = 8.5, 6.8, 1.4 Hz, 1H), 7.59 – 7.42 (m, 2H).

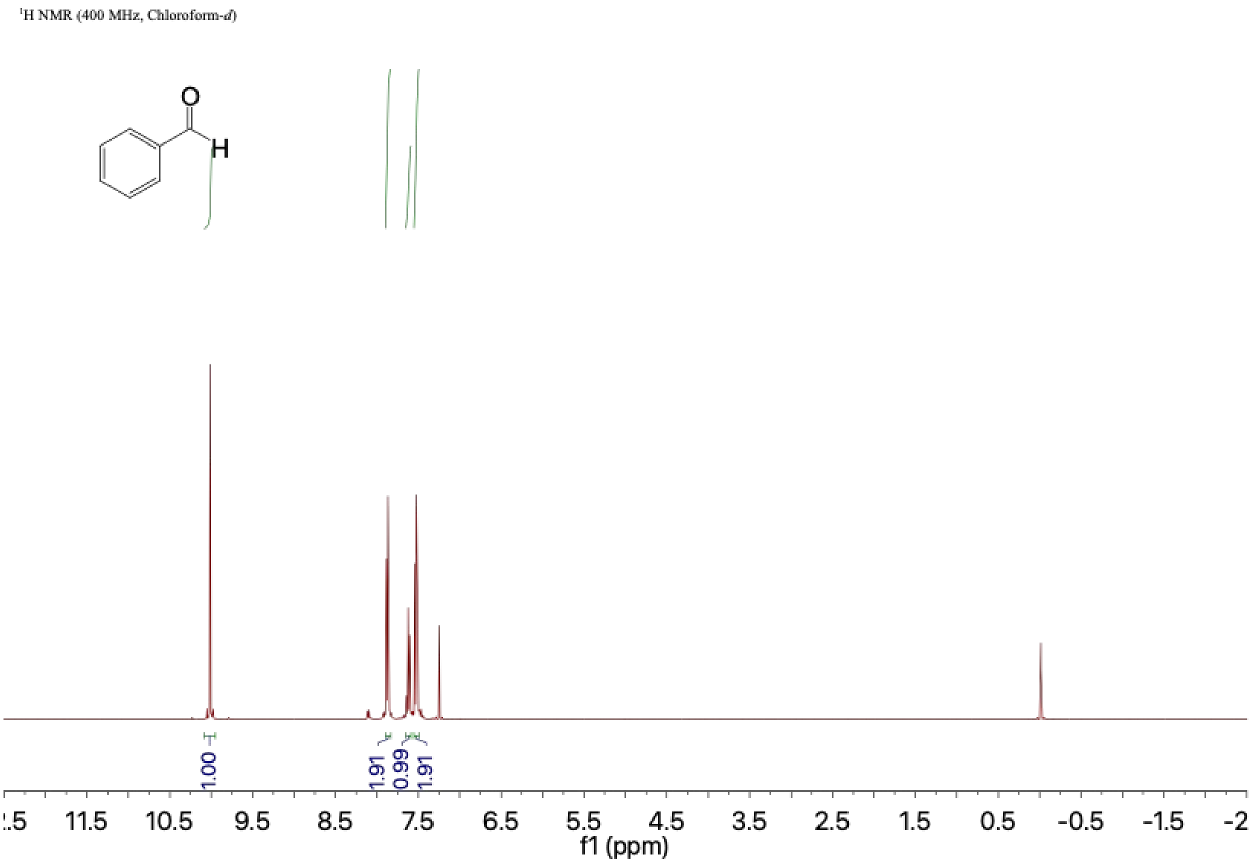

^{1}H NMR (400 MHz, Chloroform-*d*) δ 8.50 (s, 1H), 8.06 – 7.83 (m, 4H), 7.62 – 7.52 (m, 2H), 2.73 (s, 3H).

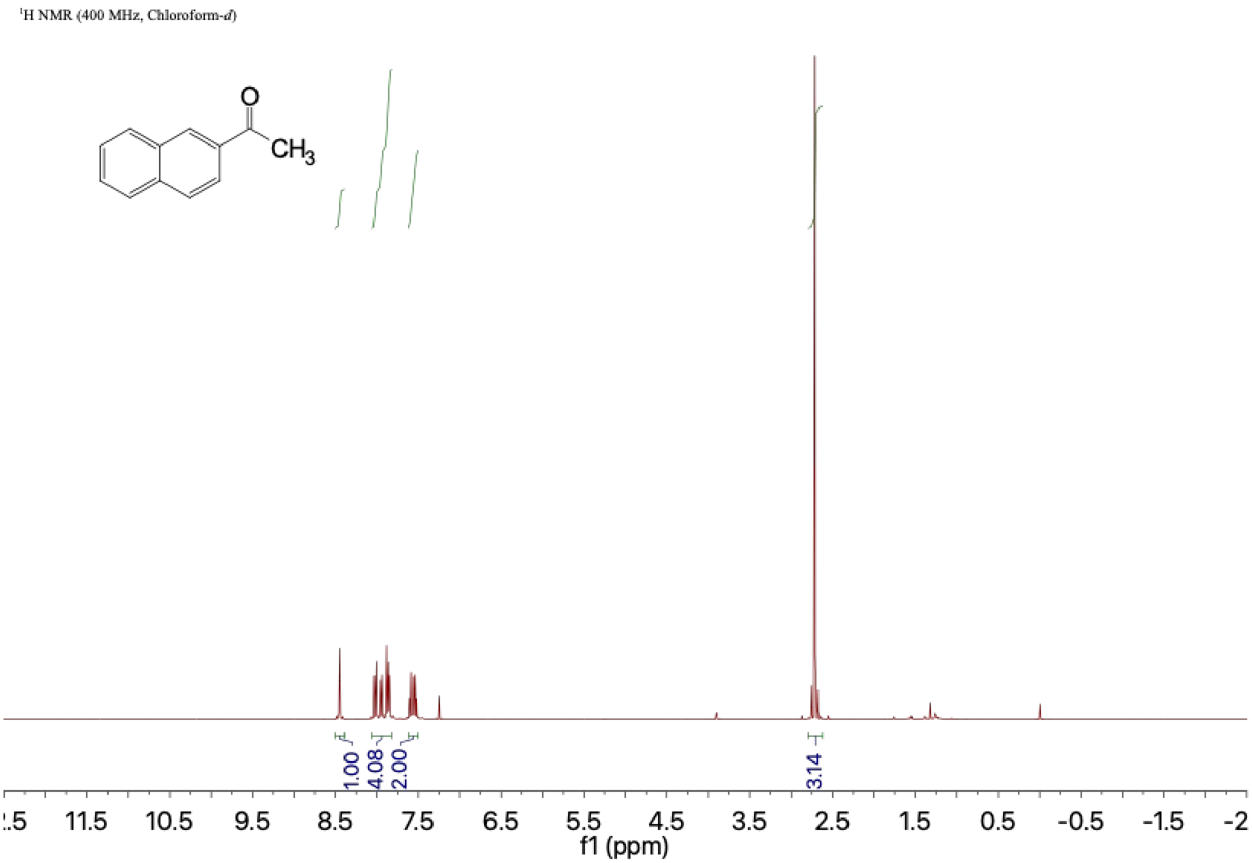

^{1}H NMR (400 MHz, Chloroform-*d*) δ 7.87 – 7.72 (m, 2H), 7.70 – 7.56 (m, 2H), 2.55 (s, 3H).

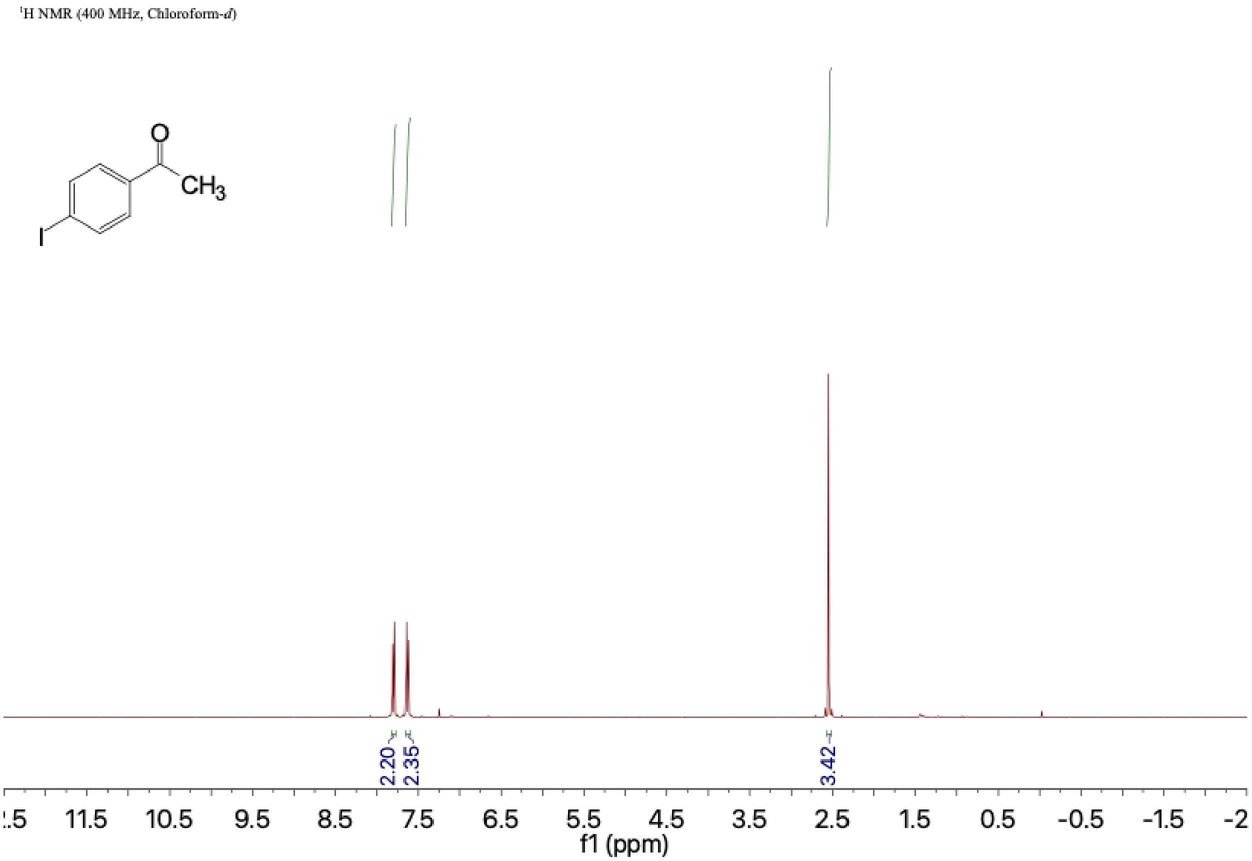

^1H NMR (400 MHz, Chloroform-*d*) 7.81 – 7.69 (m, 2H), 7.62 – 7.46 (m, 2H), 2.53 (s, 3H).

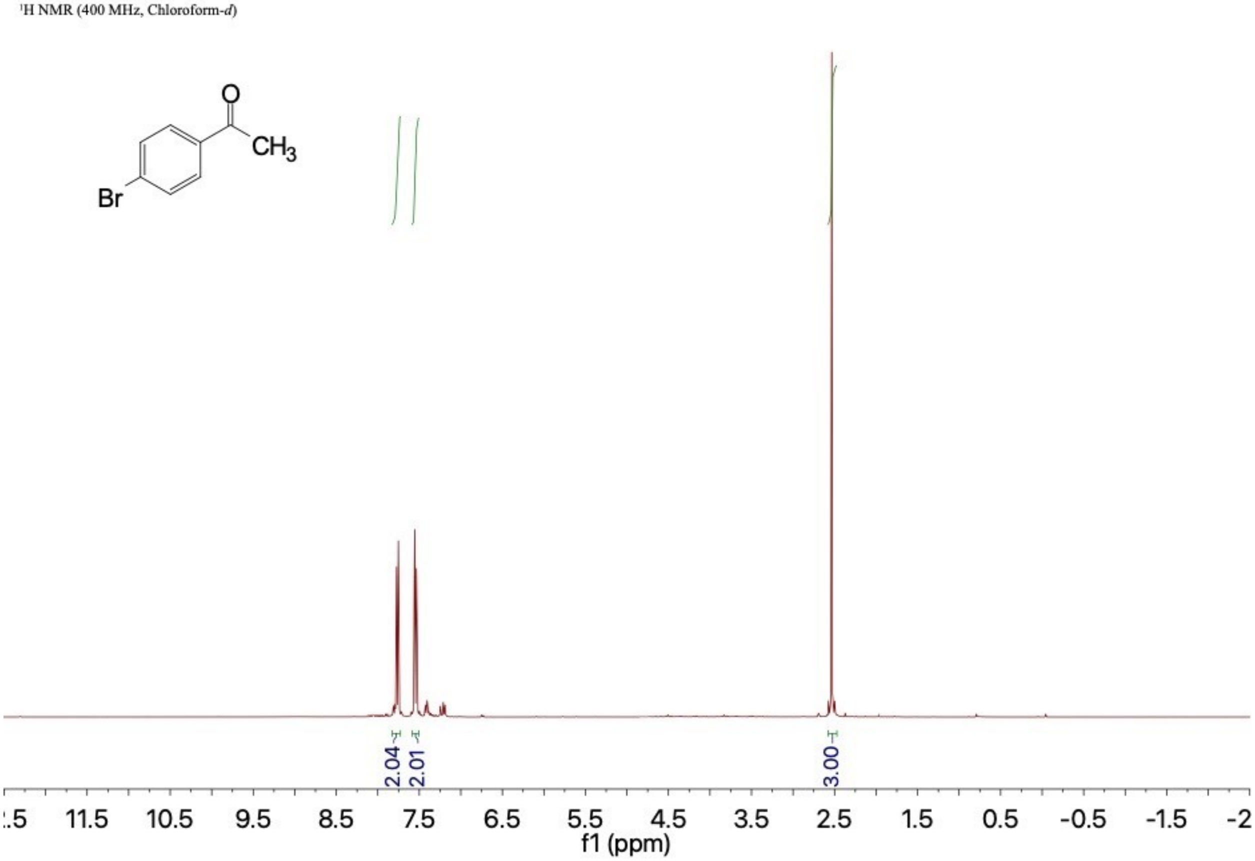

^{1}H NMR (400 MHz, Chloroform-*d*) 7.92 – 7.79 (m, 2H), 7.39 (m, 2H), 2.53 (s, 3H).

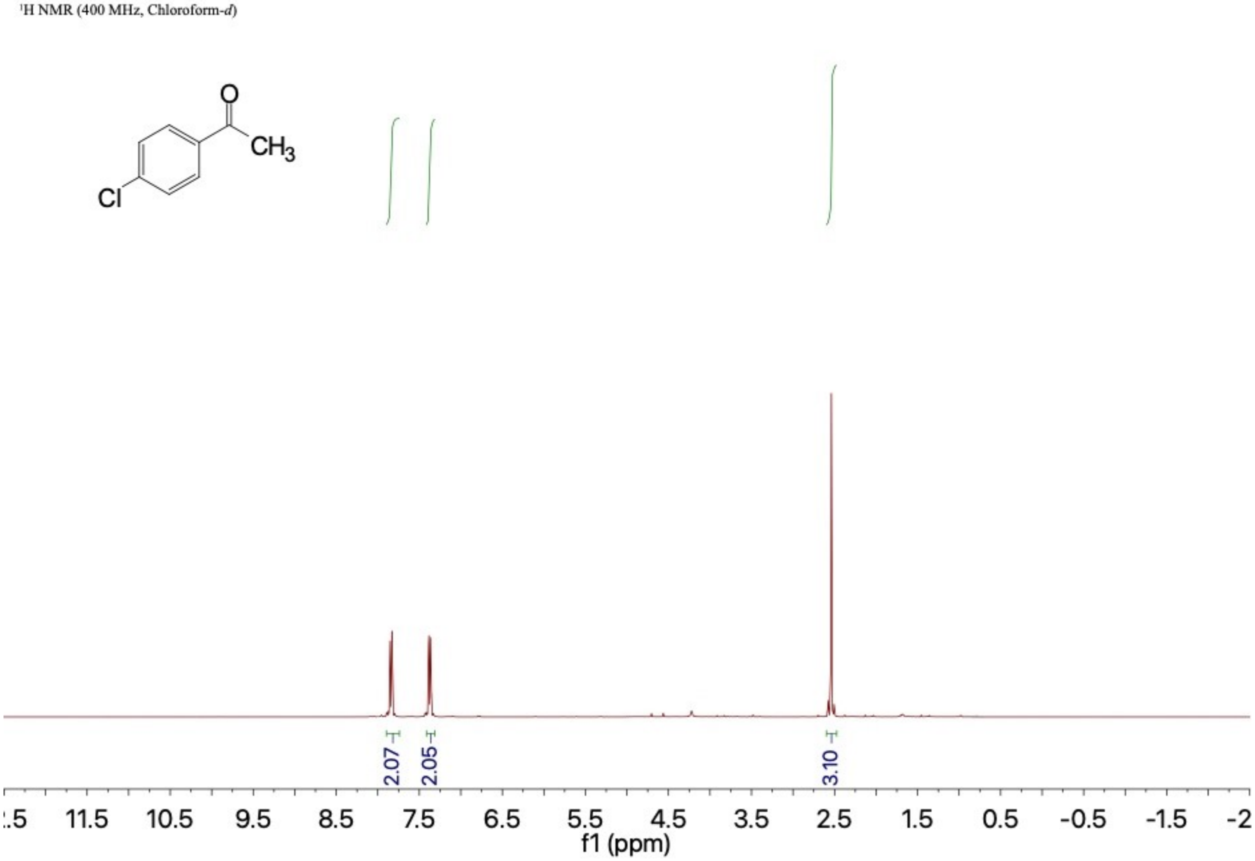

Following completion of the reaction (1 mmol scale), the mixtures were diluted to 3 mL with acetonitrile. A 50 µL portion of this solution was then diluted with 1.5 mL of acetonitrile and subsequently analyzed by GC–MS. GC/MS data presented is not intended to highlight yield, more so show evidence of formation of products.

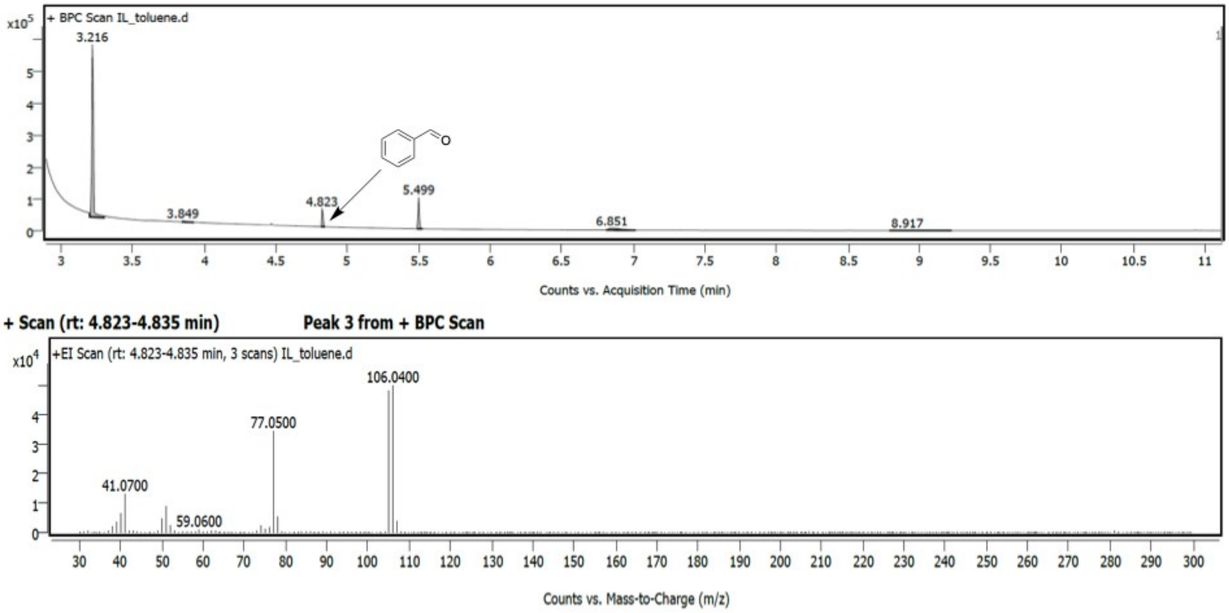

GC/MS data from Table 3 Entry 1. Representative of triplicate measurements. No starting material present.

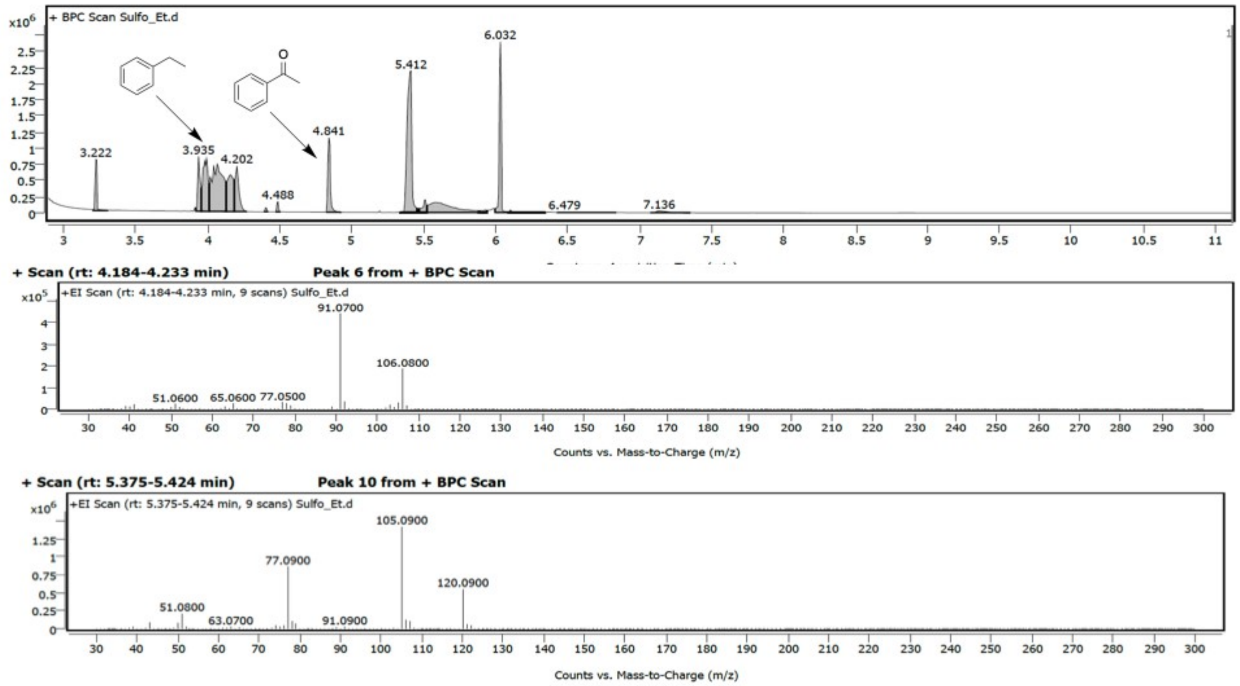

GC/MS data from Table 3 Entry 2. Representative of triplicate measurements.

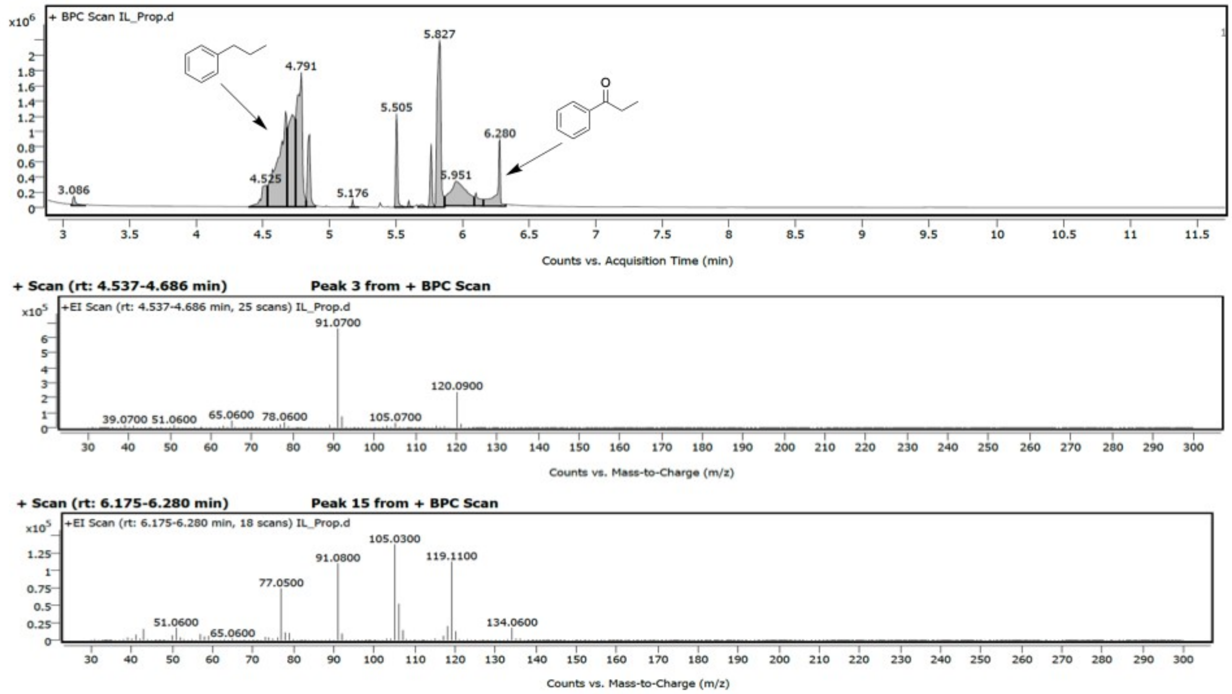

GC/MS data from Table 3 Entry 3. Representative of triplicate measurements.

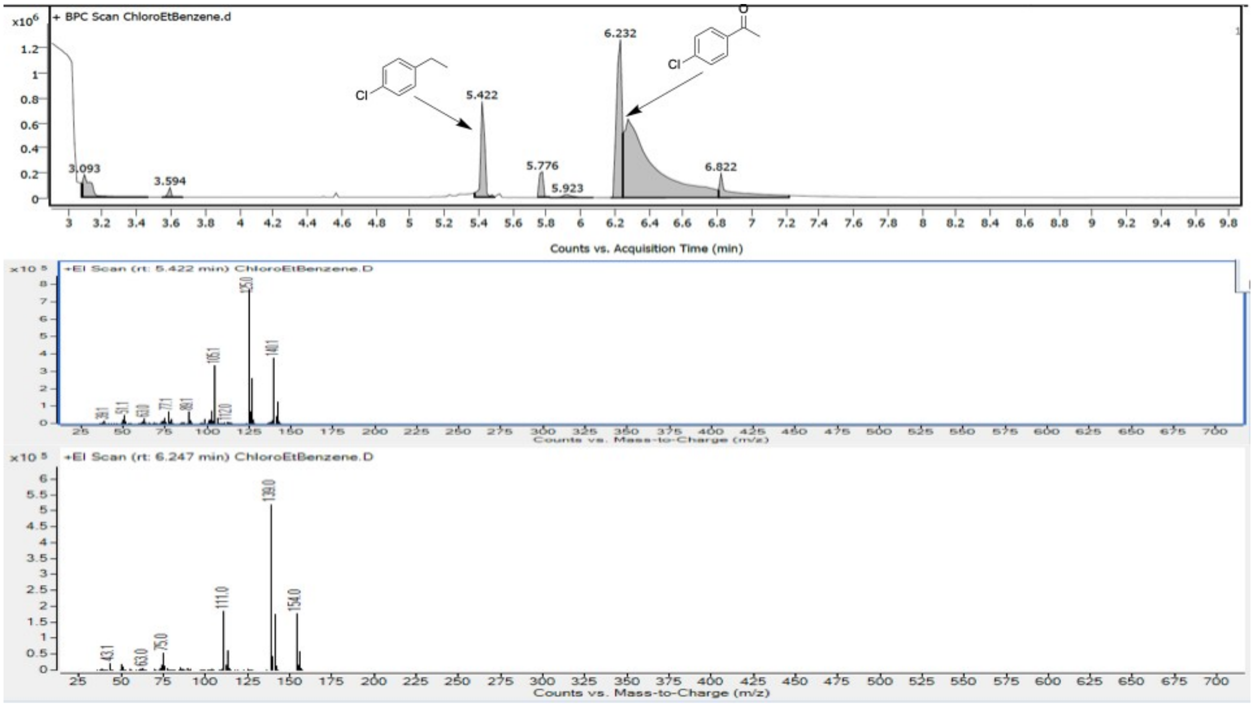

GC/MS data from Table 3 Entry 4. Representative of triplicate measurements.

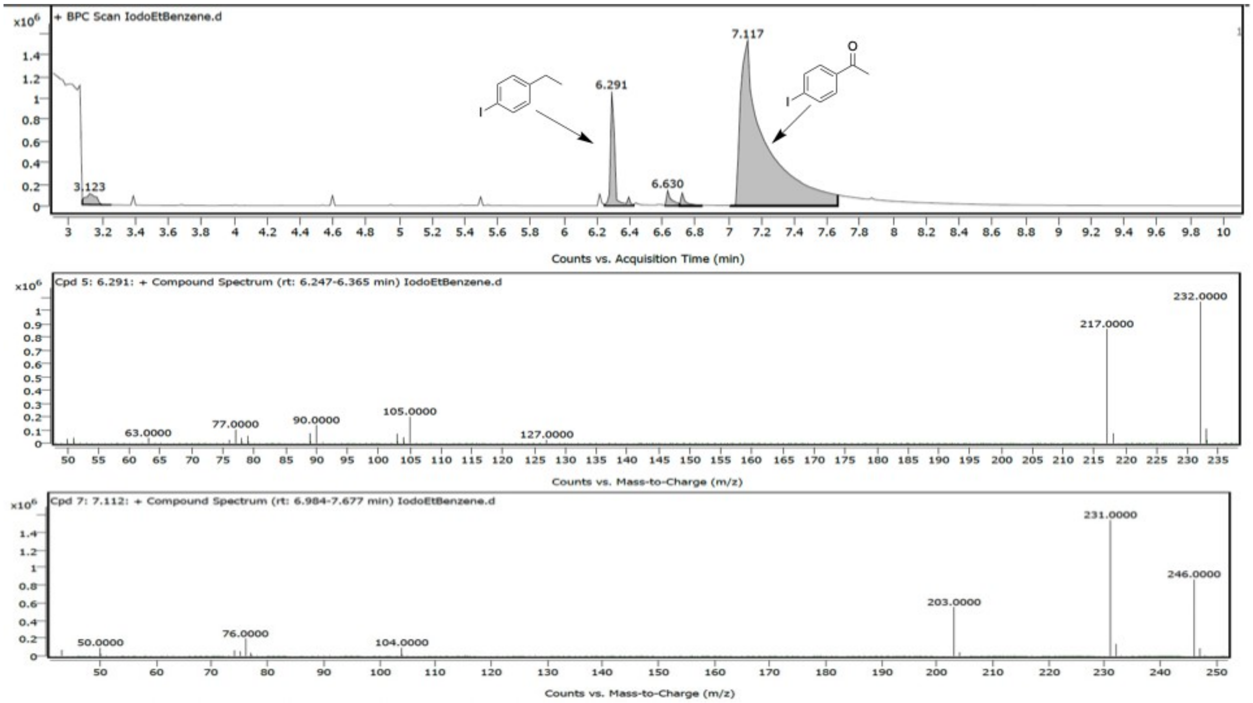

GC/MS data from Table 3 Entry 5. Representative of triplicate measurements.

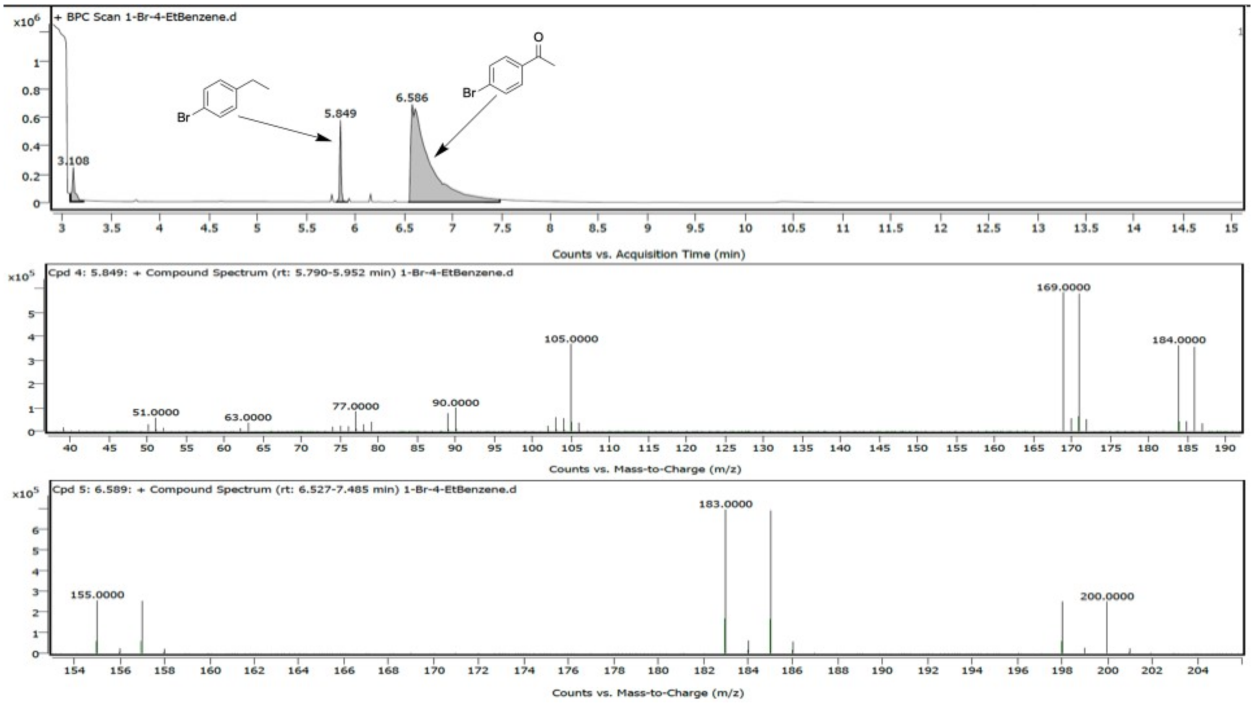

GC/MS data from Table 3 Entry 6. Representative of triplicate measurements.

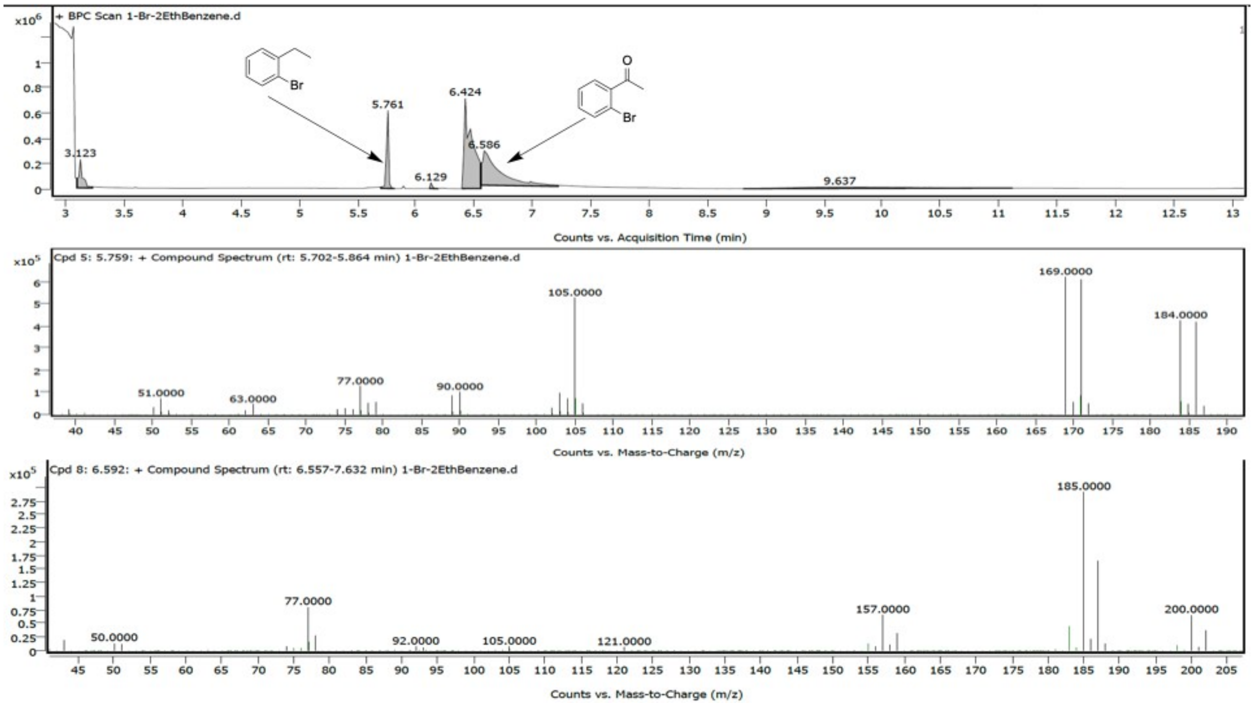

GC/MS data from Table 3 Entry 7. Representative of triplicate measurements.

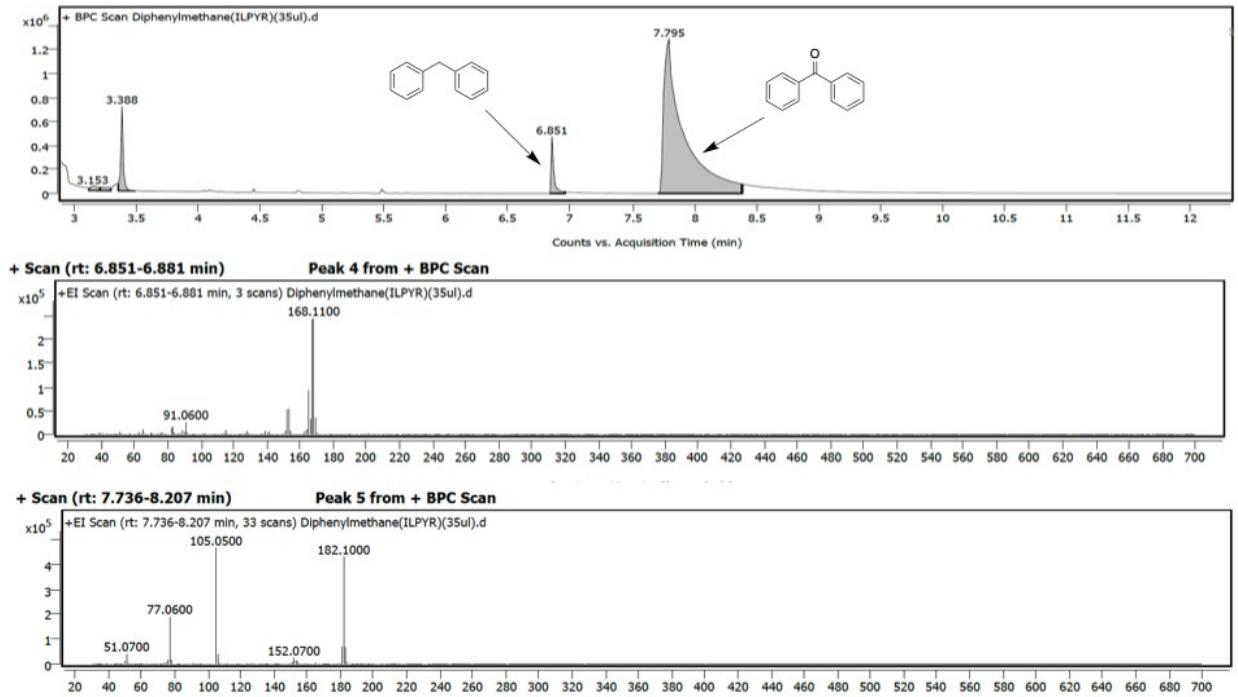

GC/MS data from Table 3 Entry 8. Representative of triplicate measurements.

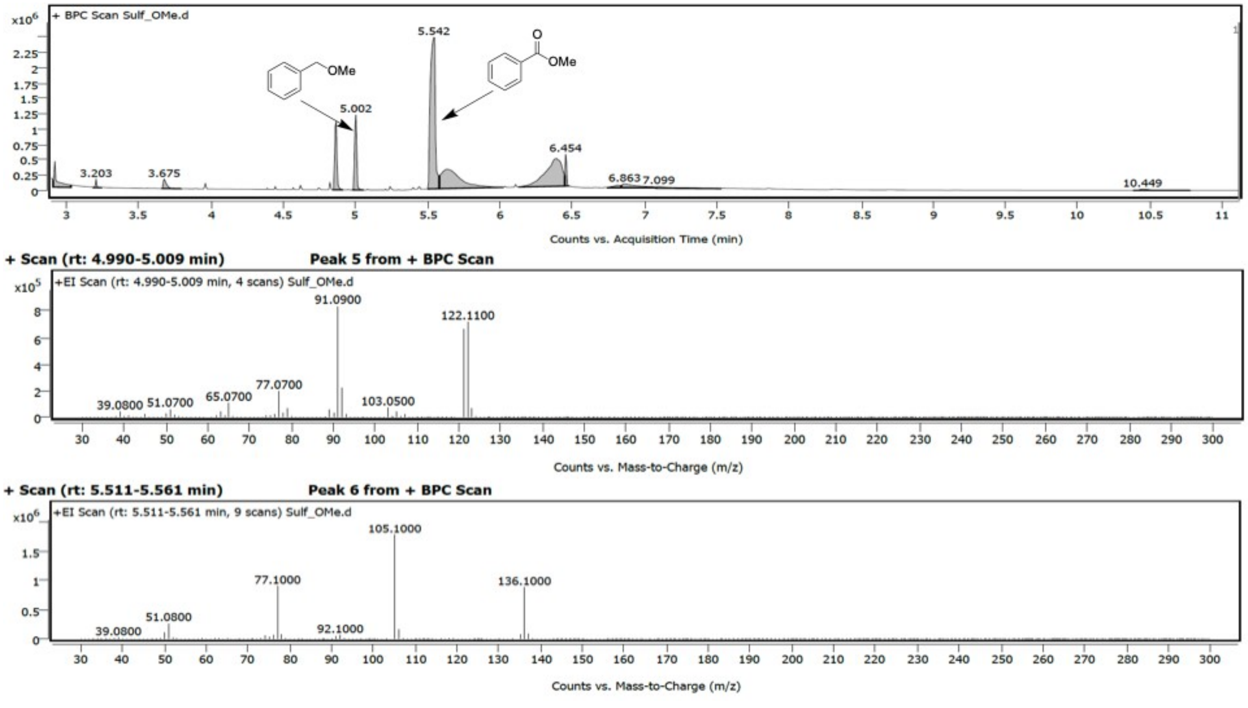

GC/MS data from Table 3 Entry 9. Representative of triplicate measurements.

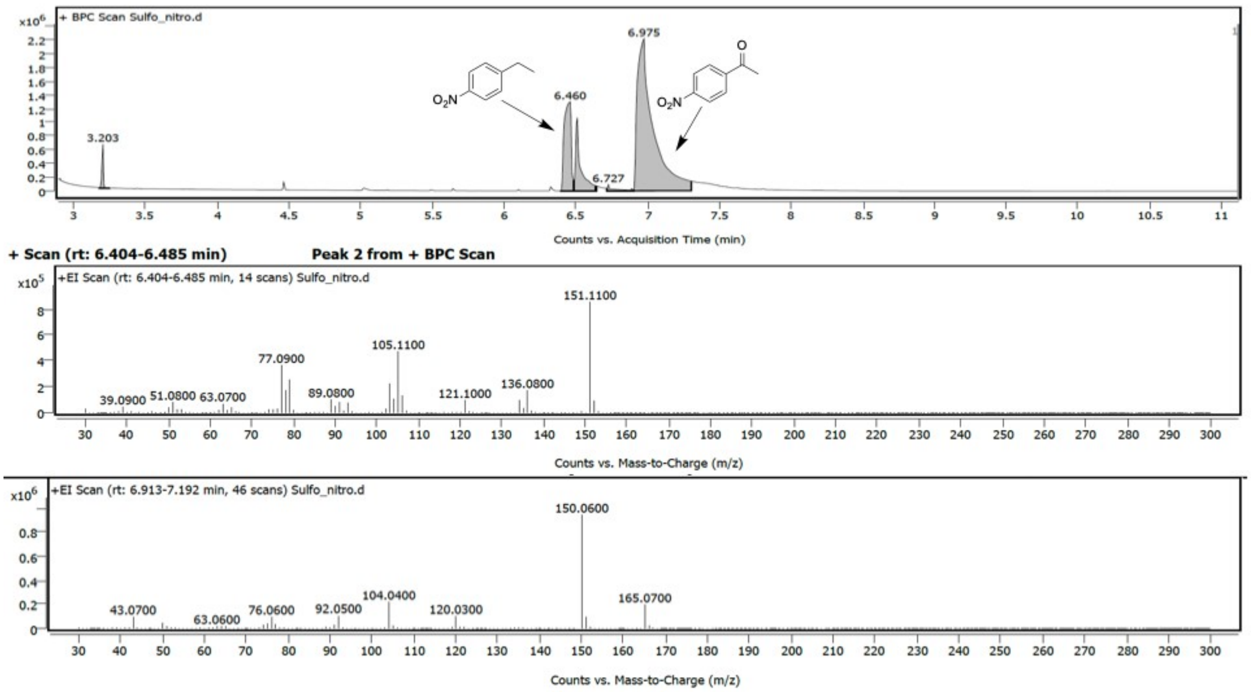

GC/MS data from Table 3 Entry 10. Representative of triplicate measurements.

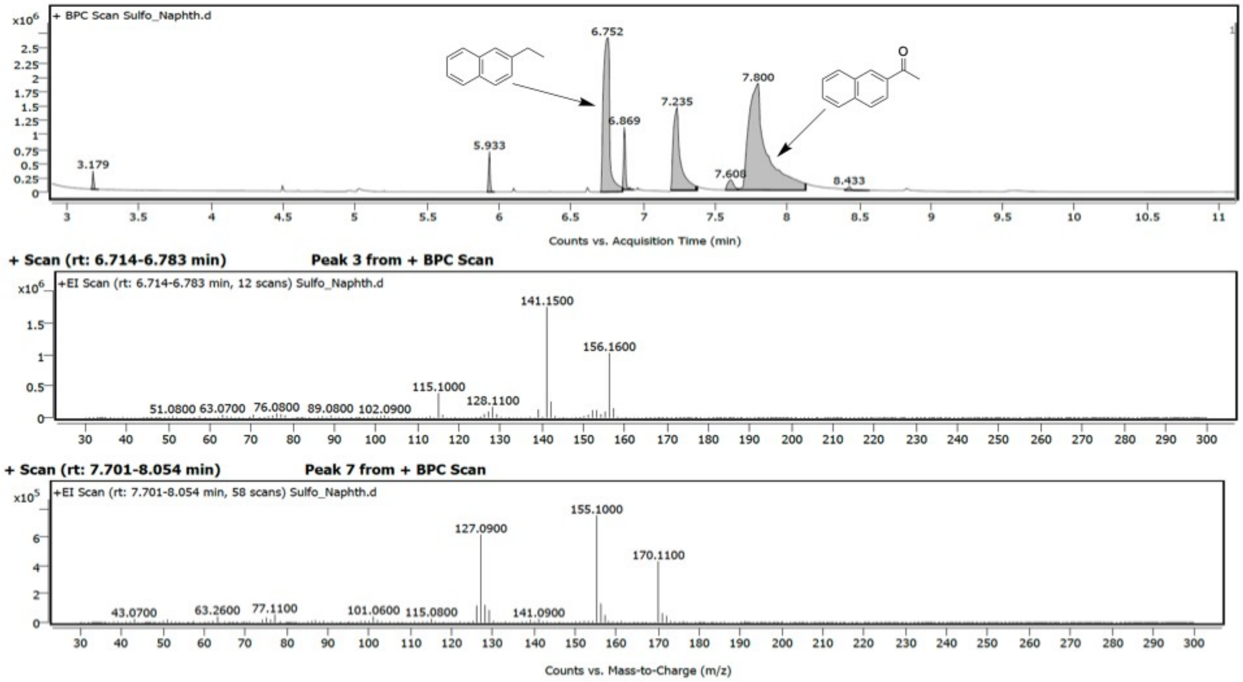

GC/MS data from Table 3 Entry 11. Representative of triplicate measurements.

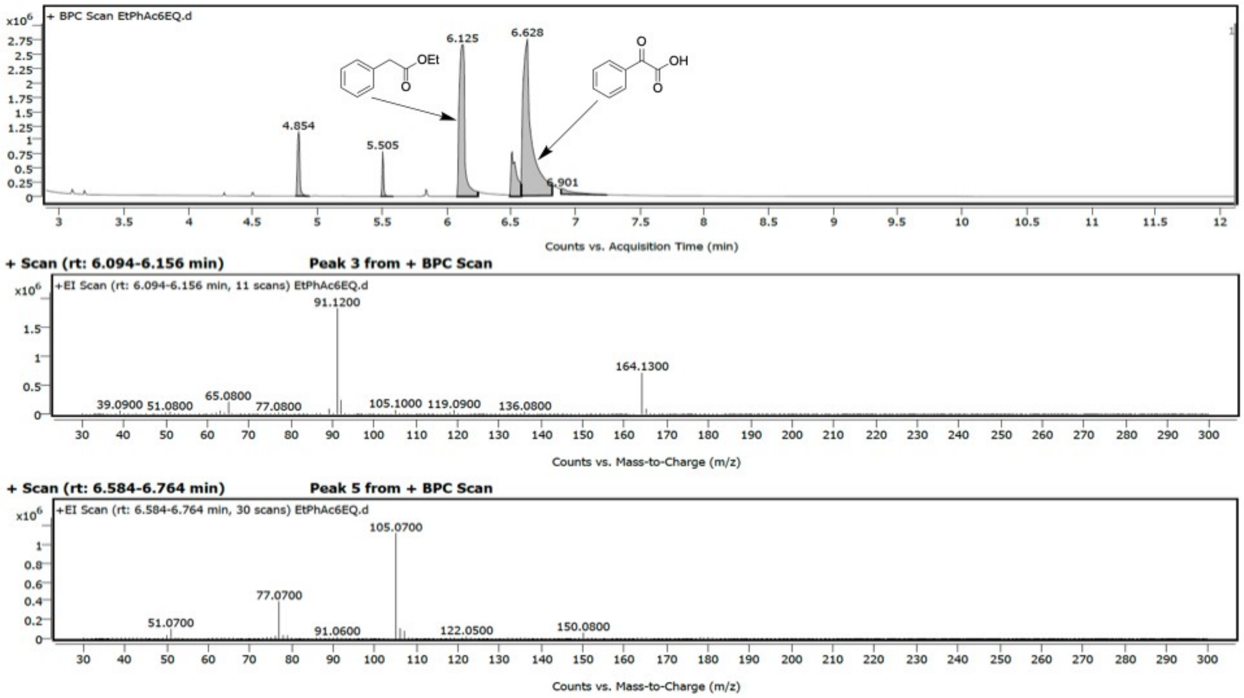

GC/MS data from Table 3 Entry 12. Representative of triplicate measurements.

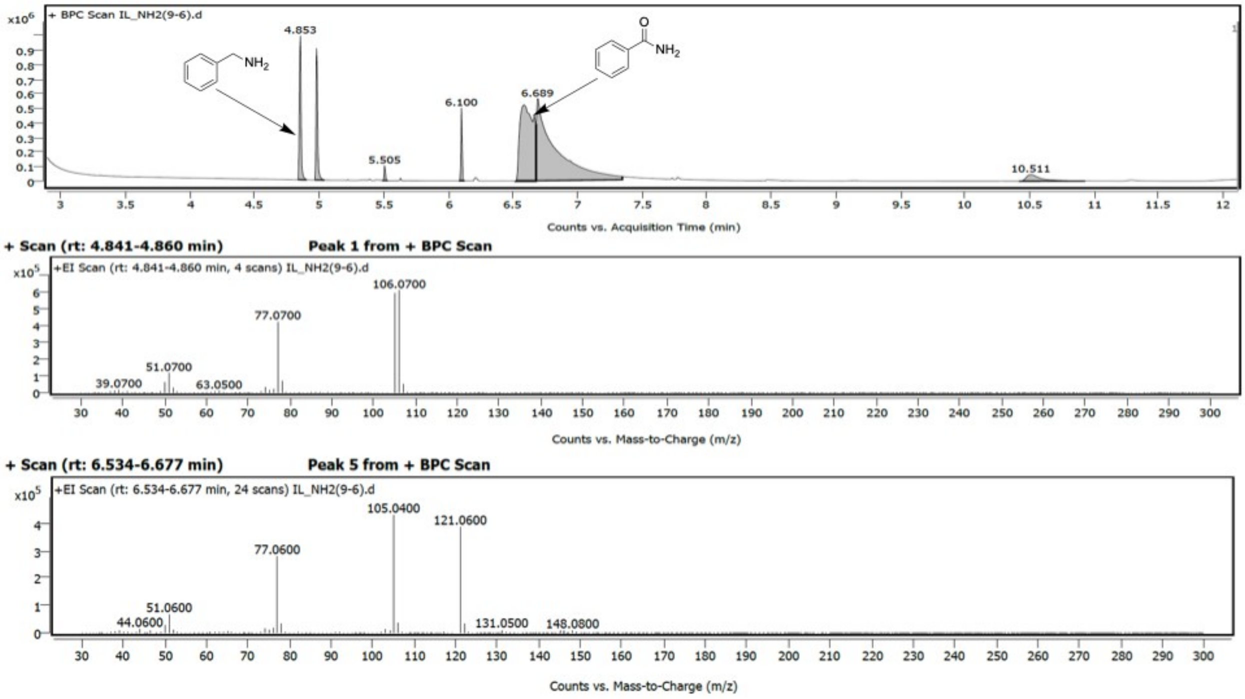

GC/MS data from Table 3 Entry 13. Representative of triplicate measurements.

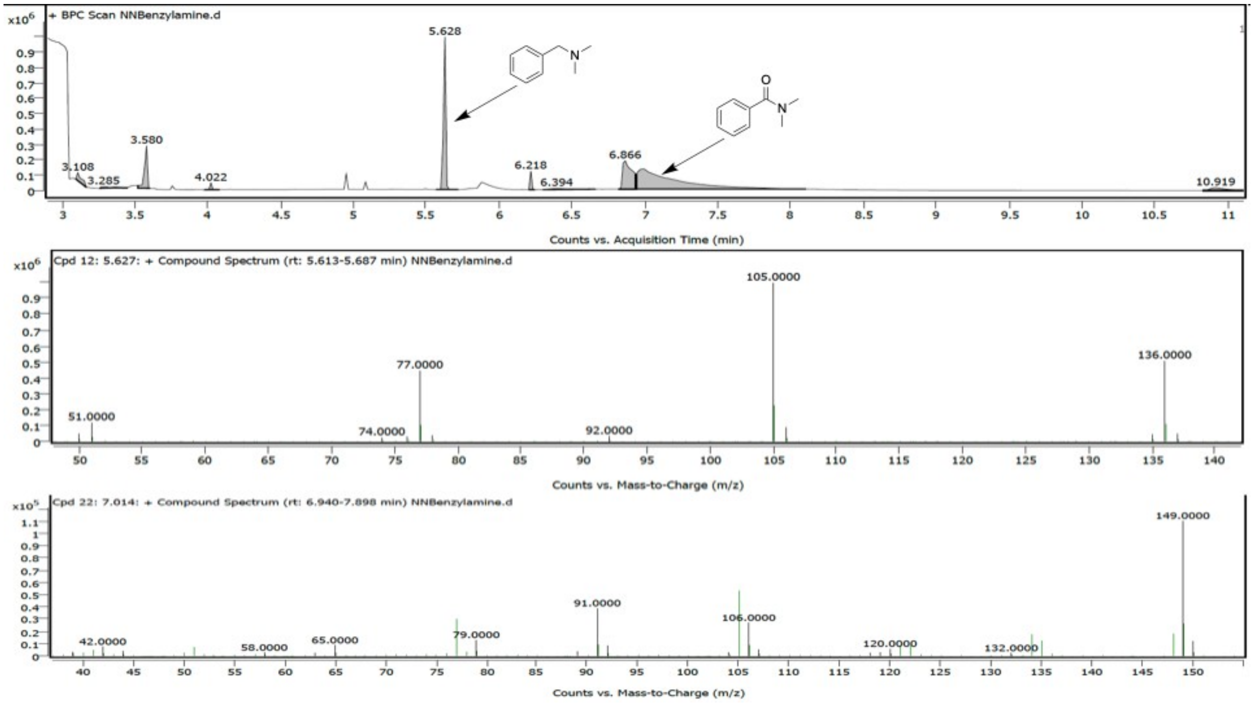

GC/MS data from Table 3 Entry 14. Representative of triplicate measurements.

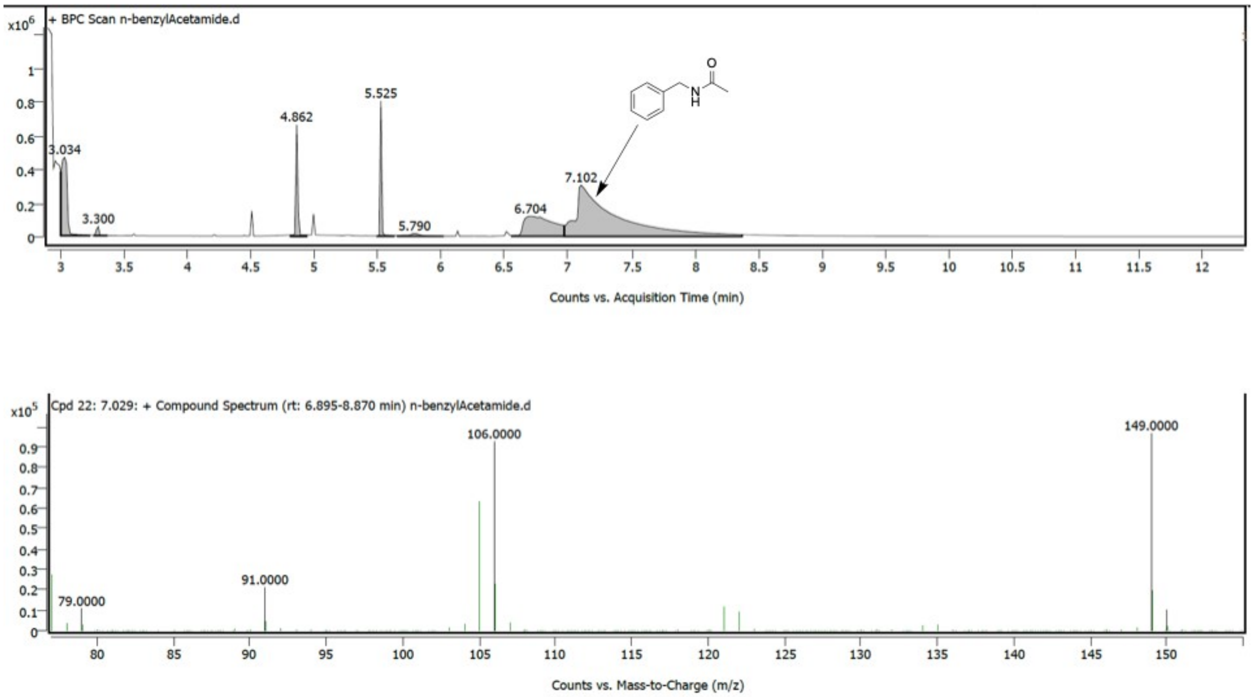

GC/MS data from Table 3 Entry 15. Representative of triplicate measurements. No formation of intended product.

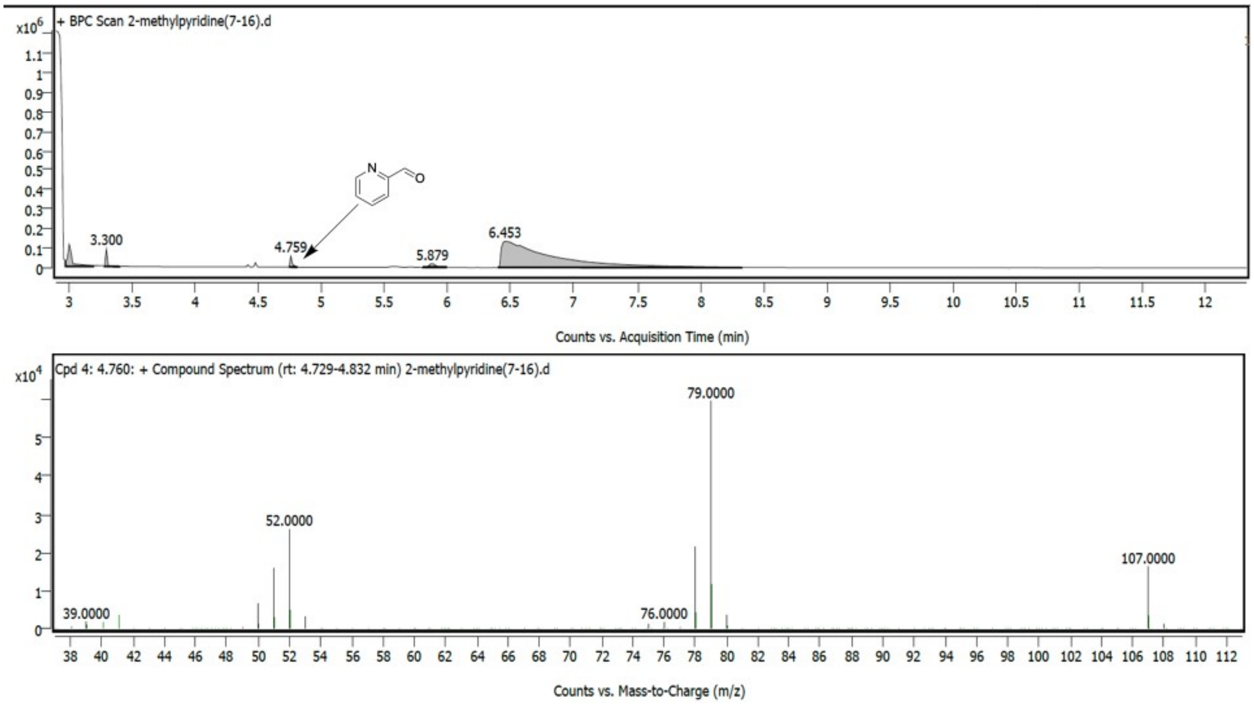

GC/MS data from Table 3 Entry 16. Representative of triplicate measurements. No evidence of starting material present

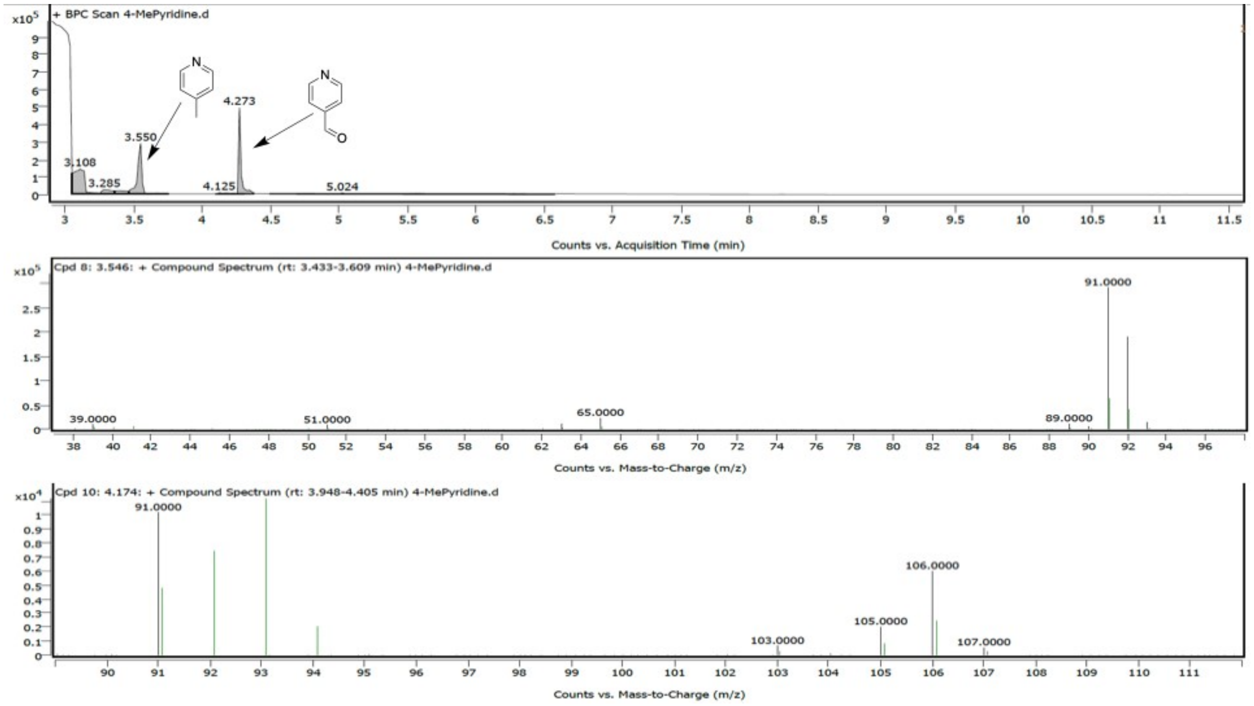

GC/MS data from Table 3 Entry 18. Representative of triplicate measurements.

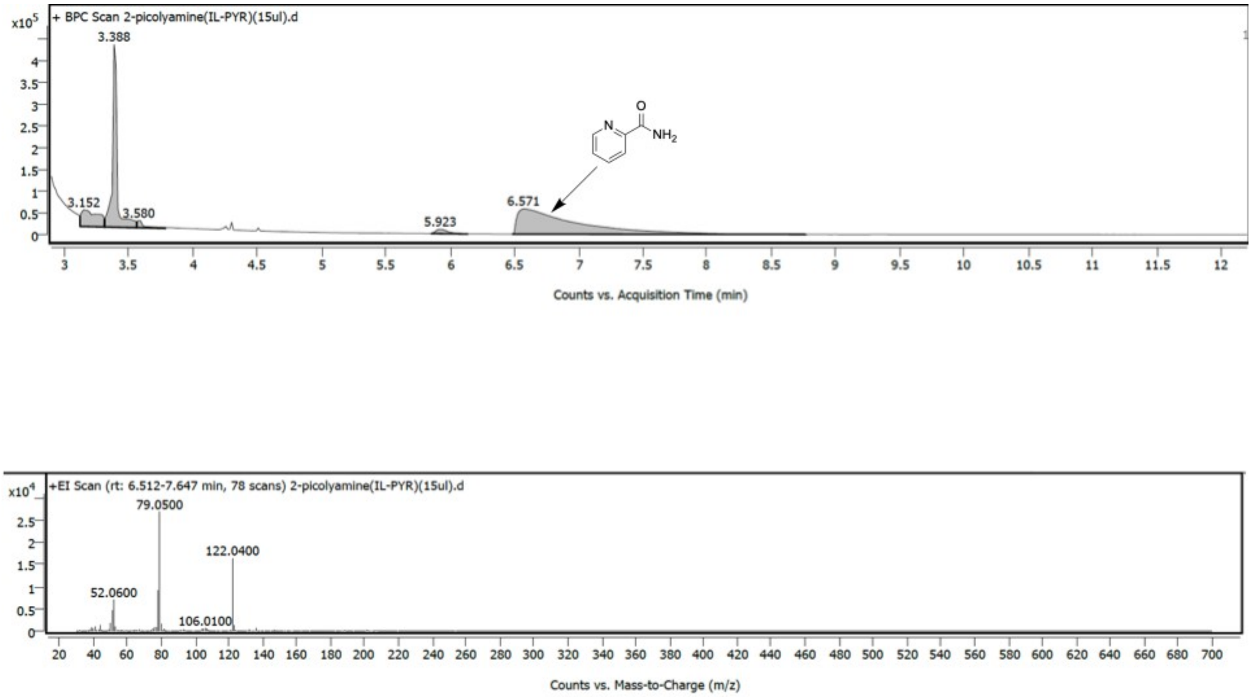

GC/MS data from Table 3 Entry 19. Representative of triplicate measurements. No evidence of starting material present.

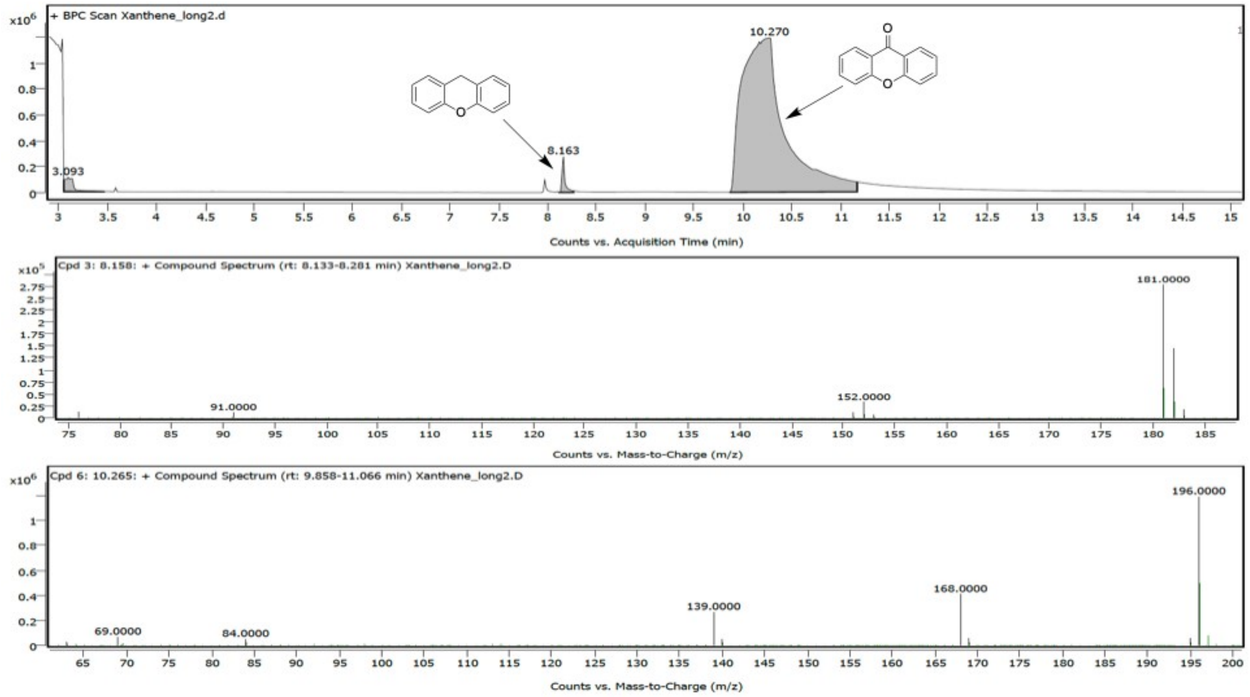

GC/MS data from Table 3 Entry 21. Representative of triplicate measurements.

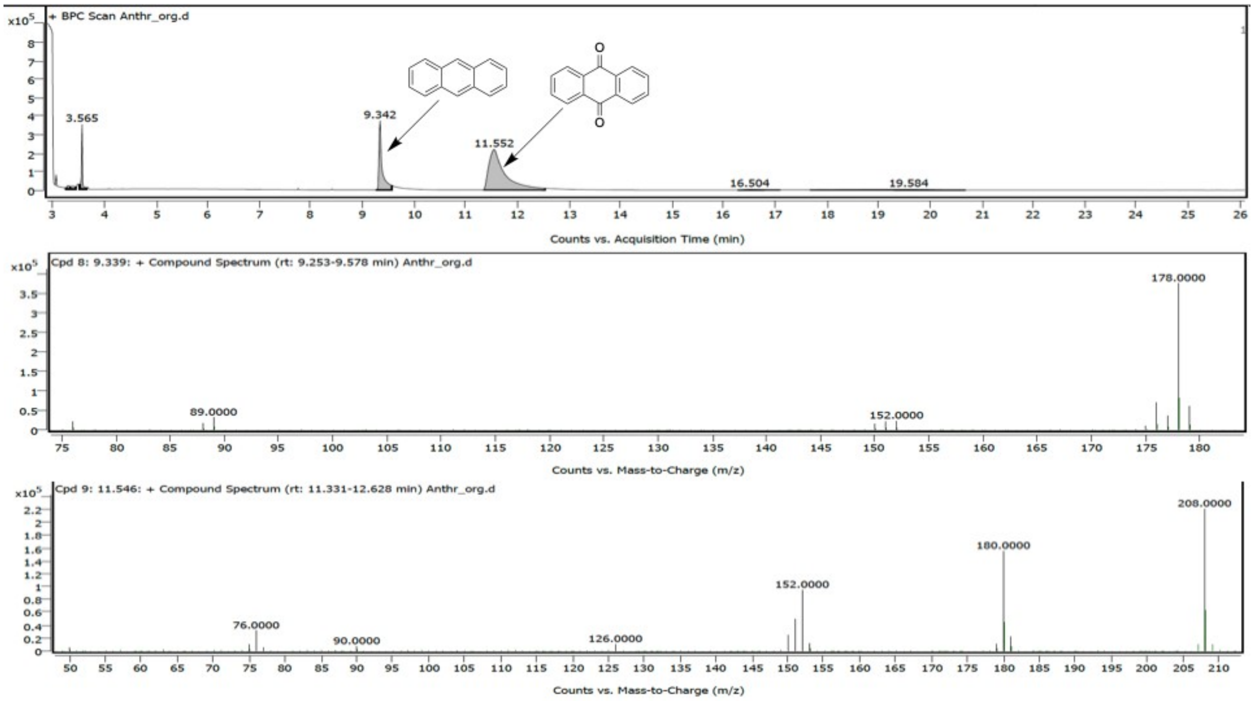

GC/MS data from Table 3 Entry 22. Representative of triplicate measurements.

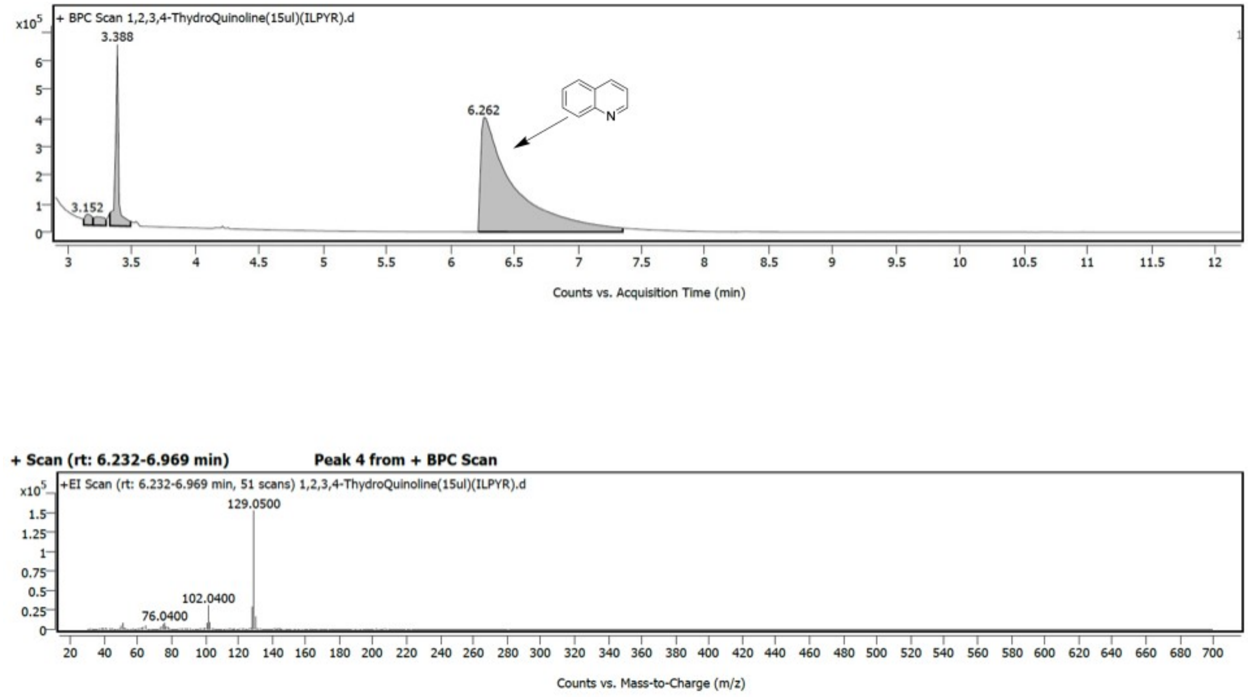

GC/MS data from Table 3 Entry 23. Representative of triplicate measurements. No evidence of starting material present.

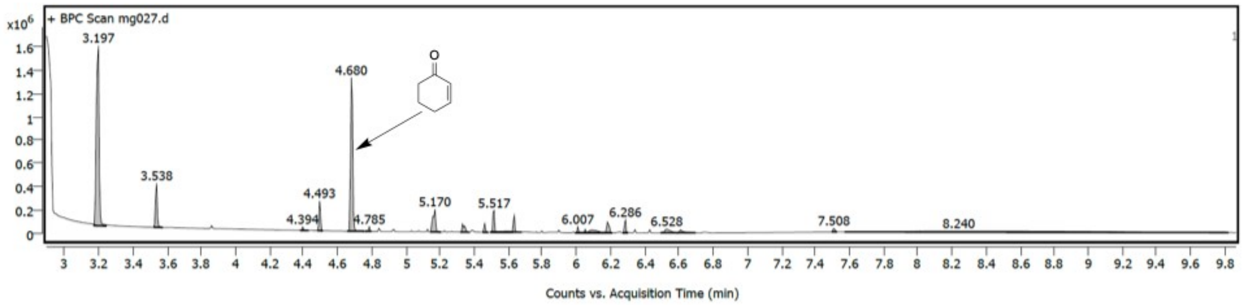

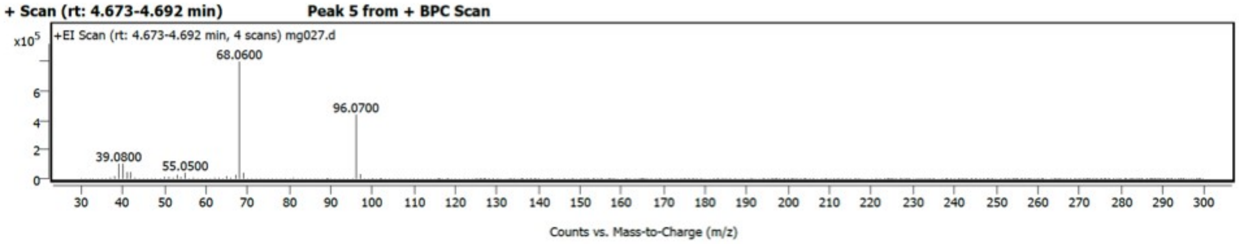

GC/MS data from Table 2 Entry 1. Representative of triplicate measurements. No evidence of starting material present.

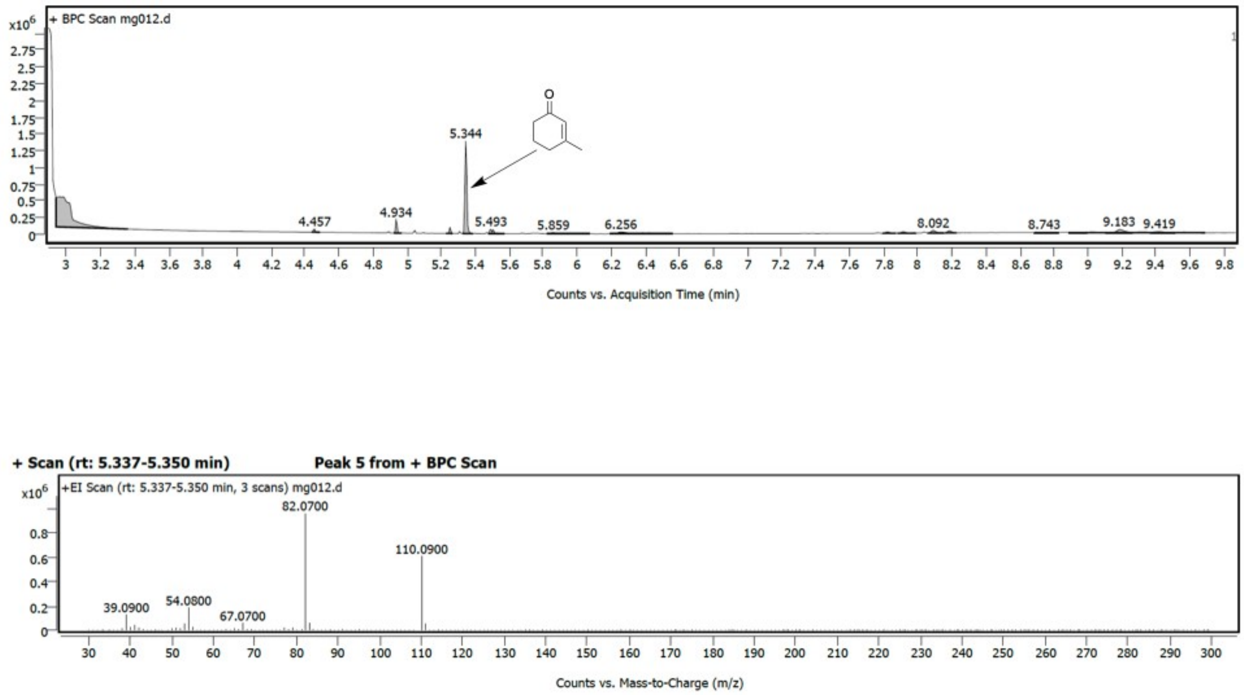

GC/MS data from Table 2 Entry 2. Representative of triplicate measurements. No evidence of starting material present.

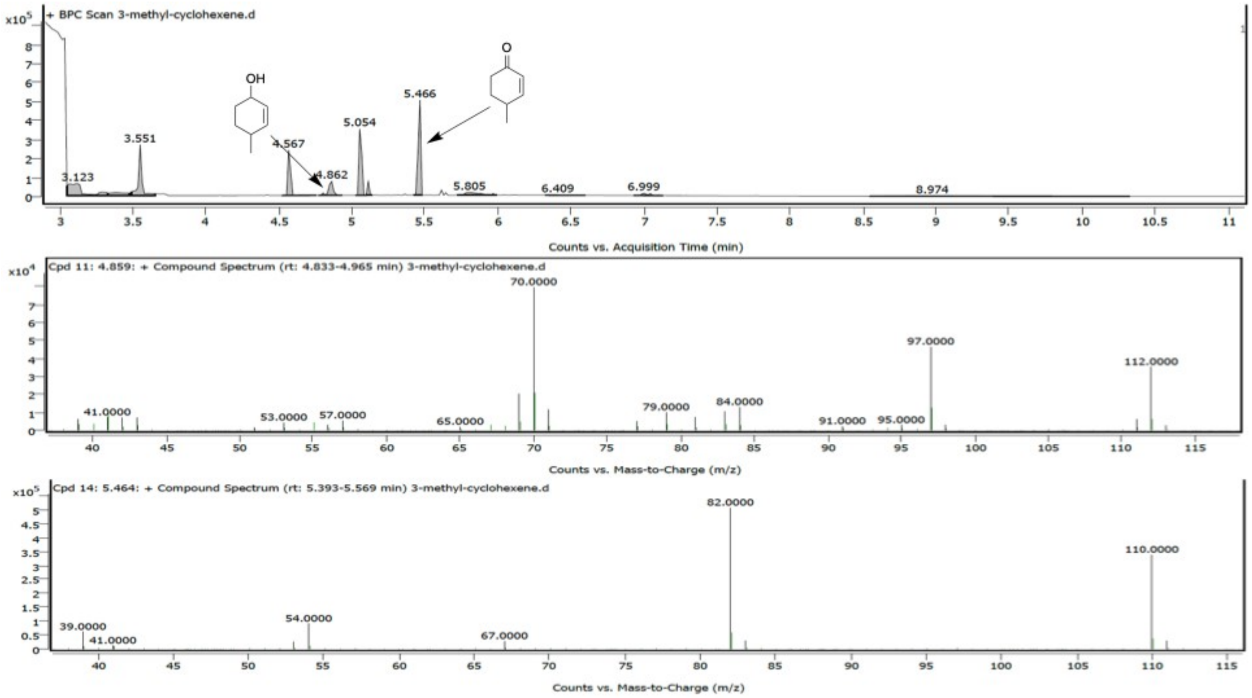

GC/MS data from Table 2 Entry 3. Representative of triplicate measurements.

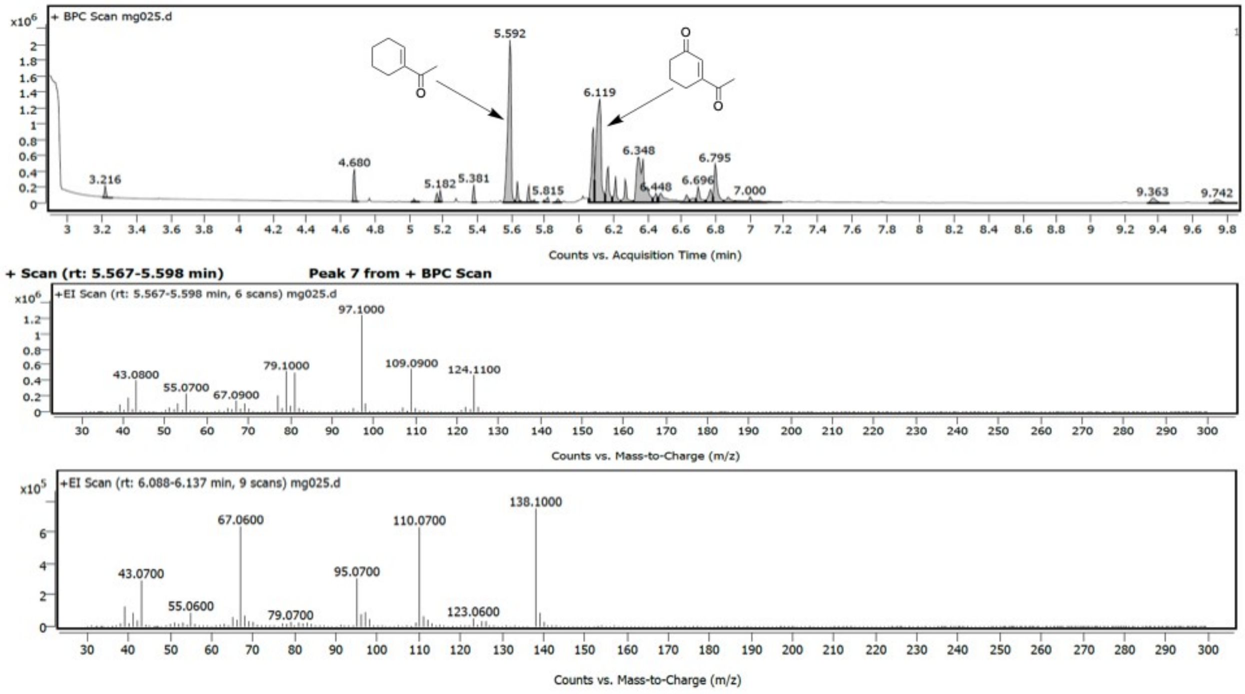

GC/MS data from Table 2 Entry 4. Representative of triplicate measurements.

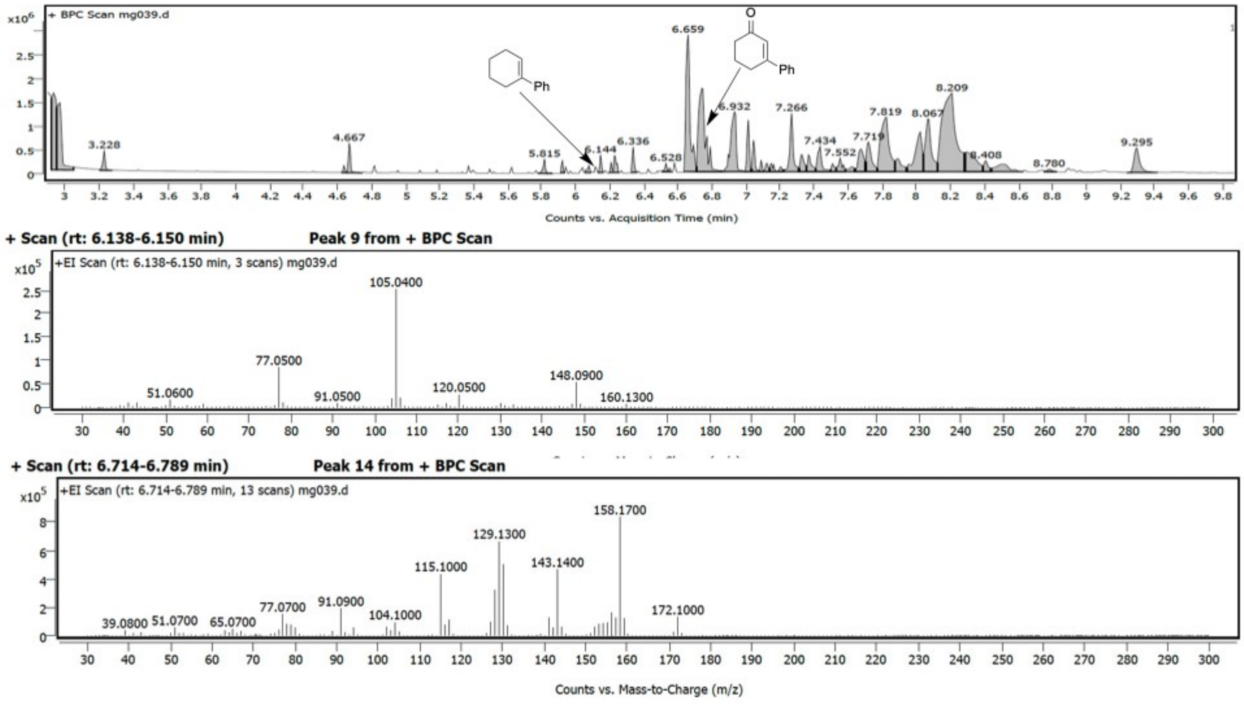

GC/MS data from Table 2 Entry 5. Representative of triplicate measurements.

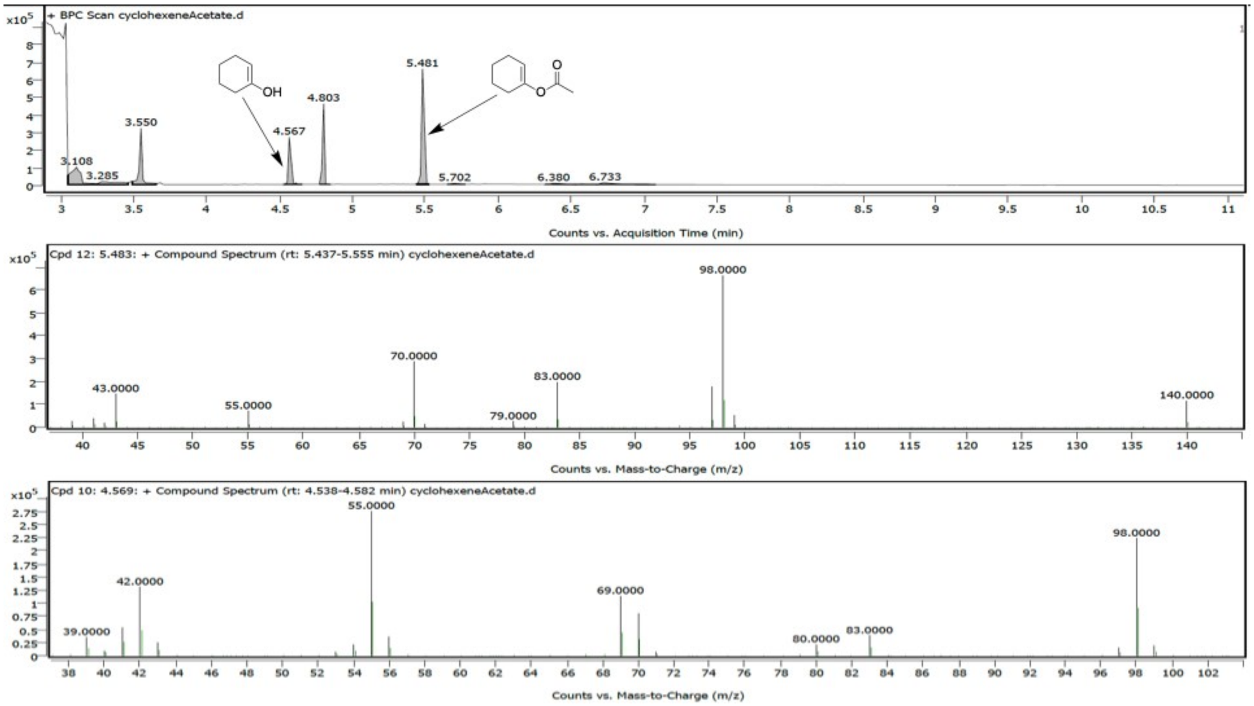

GC/MS data from Table 2 Entry 6. Representative of triplicate measurements.

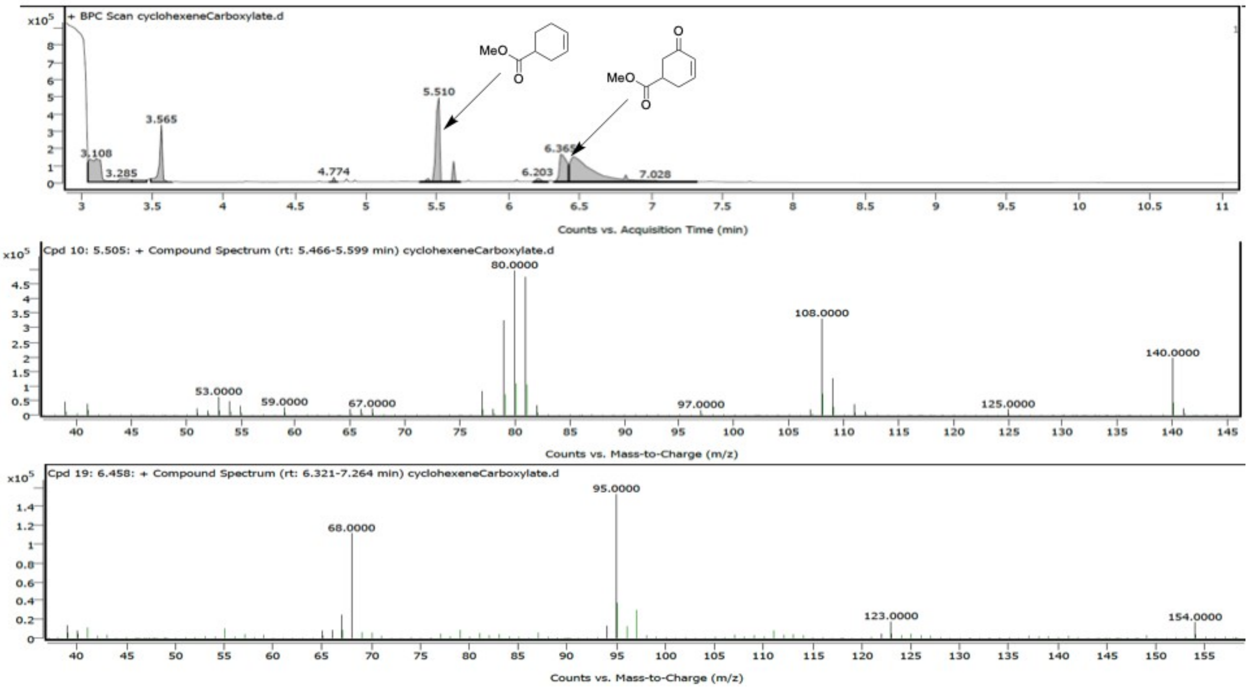

GC/MS data from Table 2 Entry 7. Representative of triplicate measurements.

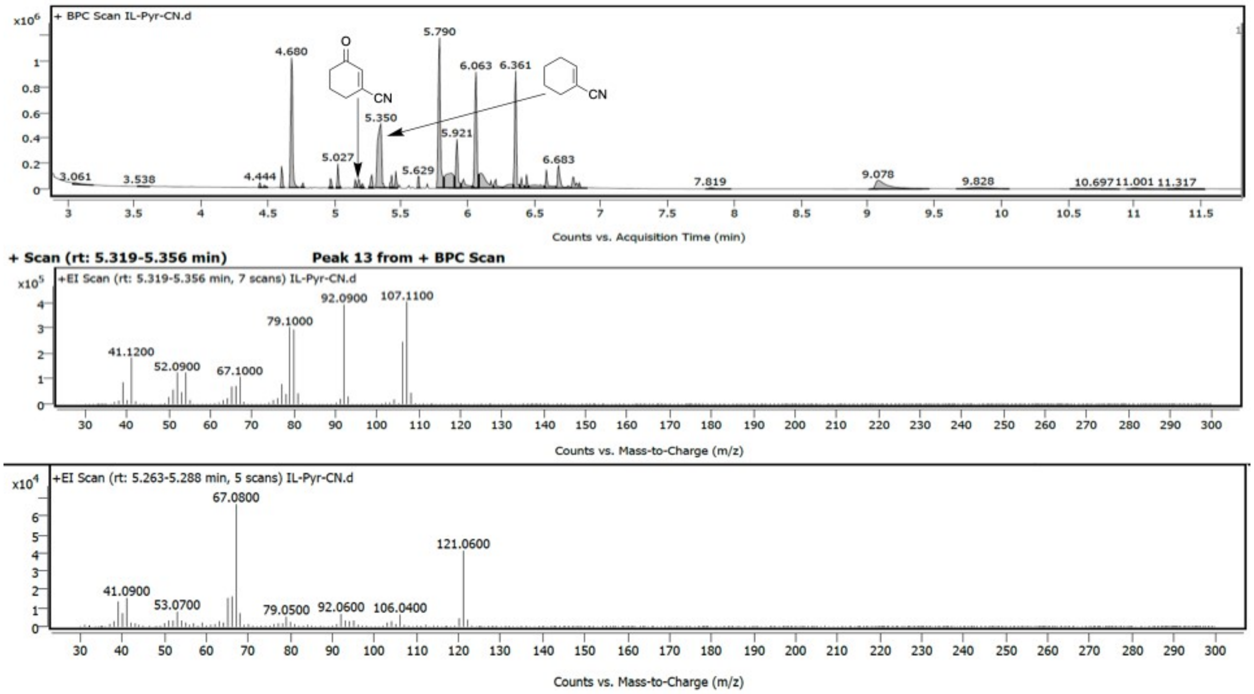

GC/MS data from Table 2 Entry 8. Representative of triplicate measurements.

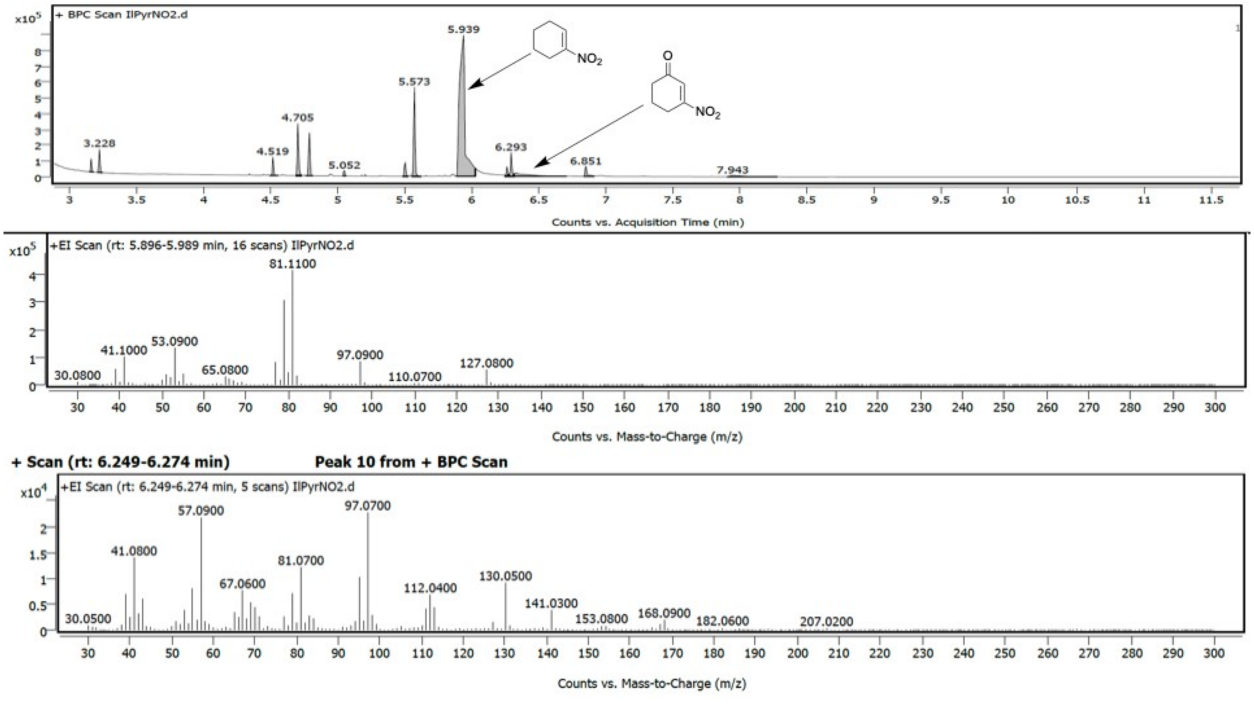

GC/MS data from Table 2 Entry 9. Representative of triplicate measurements.

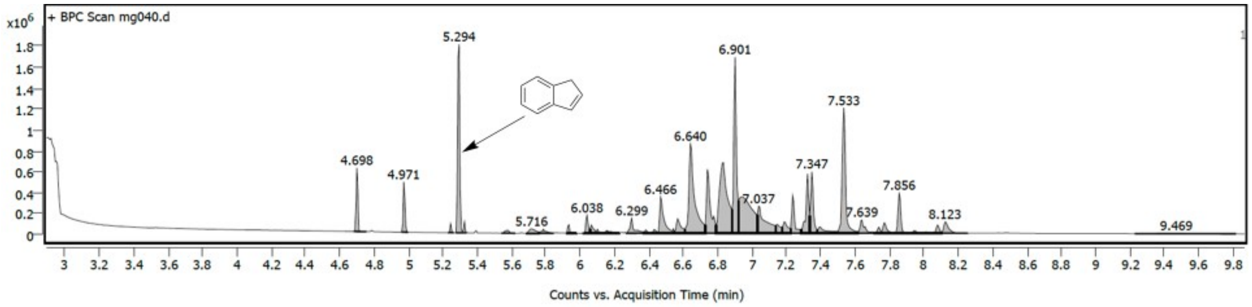

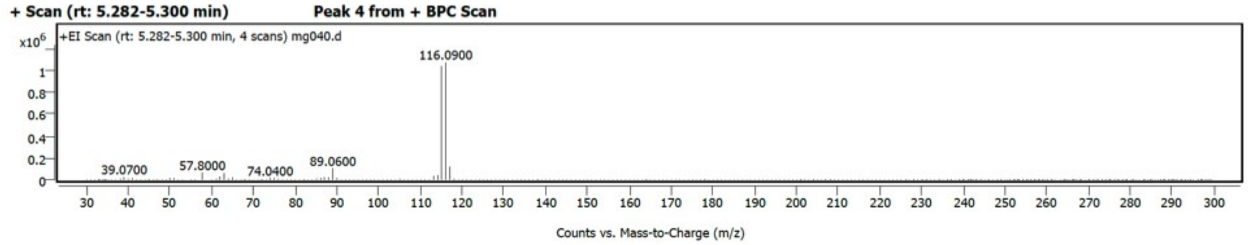

GC/MS data from Table 2 Entry 10. Representative of triplicate measurements. No product formation present

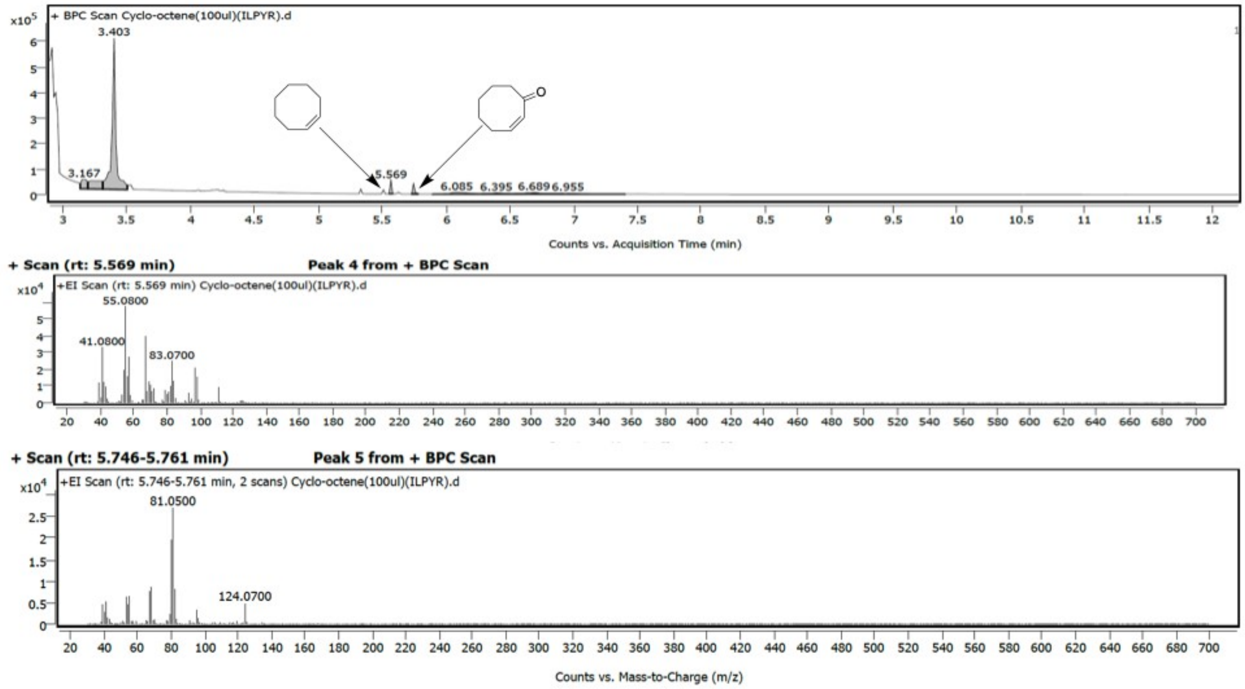

GC/MS data from Table 2 Entry 11. Representative of triplicate measurements.

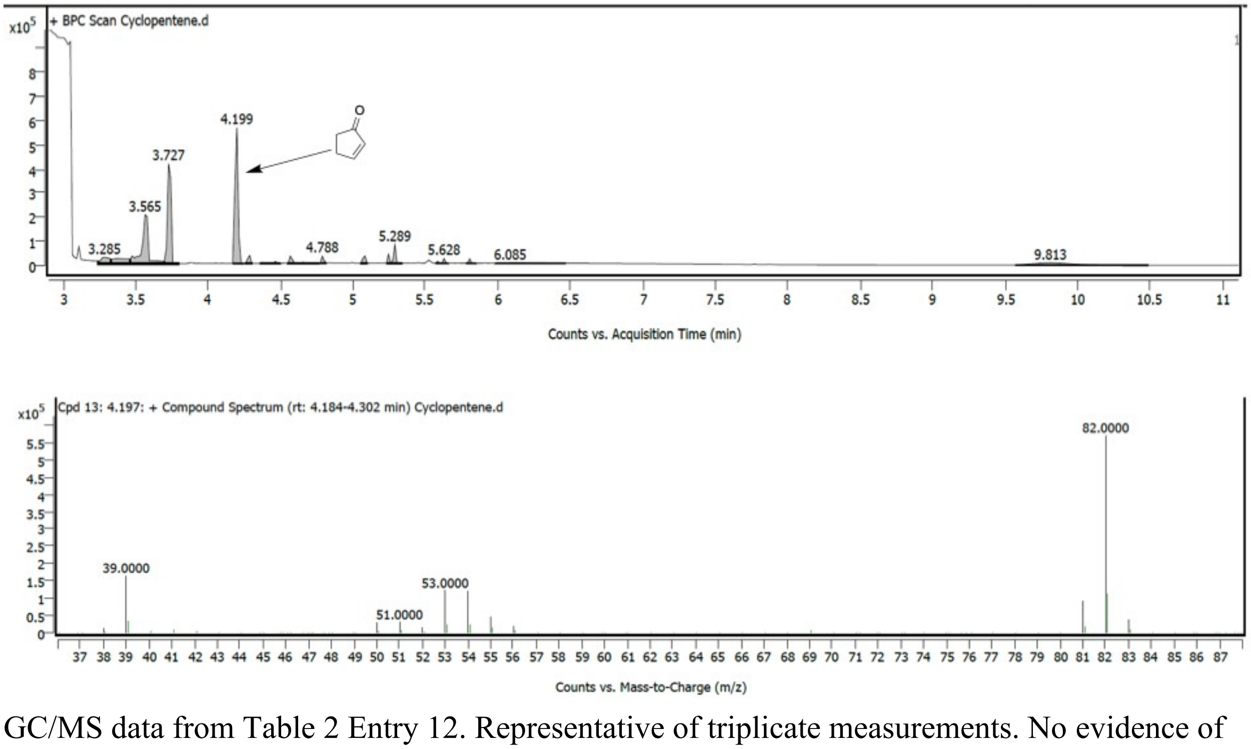

GC/MS data from Table 2 Entry 12. Representative of triplicate measurements. No evidence of starting material present.

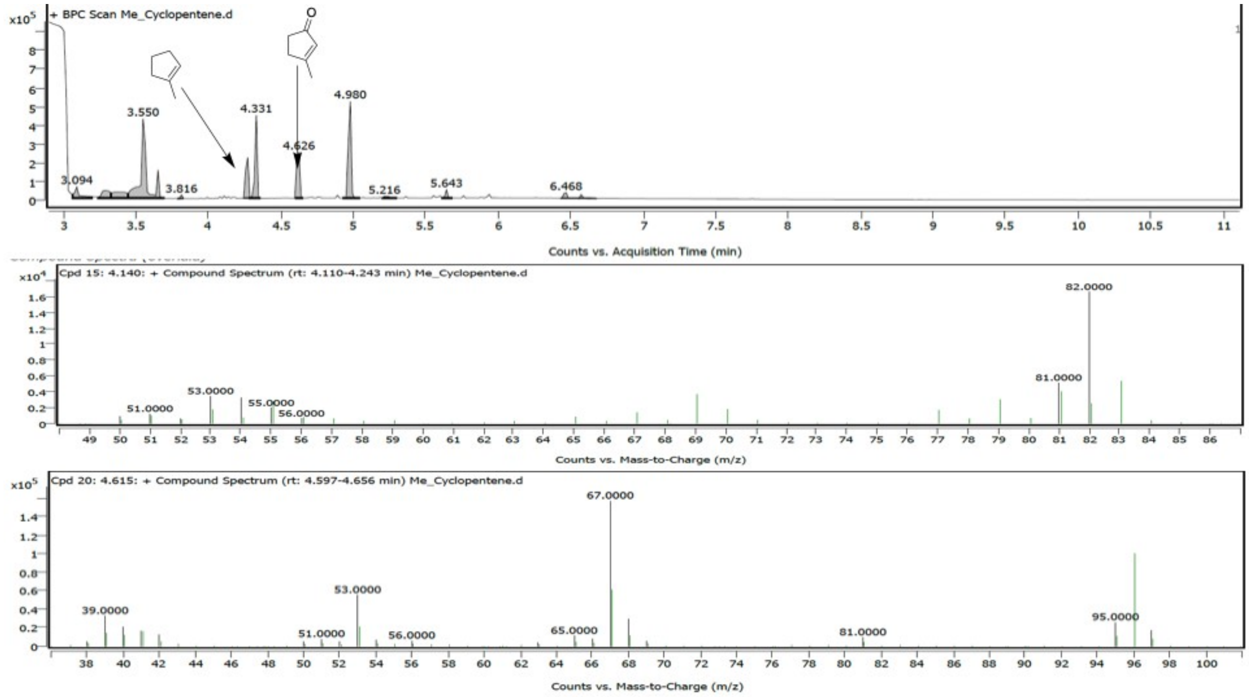

GC/MS data from Table 2 Entry 13. Representative of triplicate measurements.
